# Electromyography (EMG) Signal Processing to Evaluate Low-Frequency Tremors

**DOI:** 10.3390/s26010157

**Published:** 2025-12-25

**Authors:** Samantha O’Sullivan, Mark Daly, Niall Murray, Thiago Braga Rodrigues

**Affiliations:** Department of Engineering & Technology, The Technological University of the Shannon: Midlands Midwest, N37 HD68 Athlone, Ireland; mark.daly@tus.ie (M.D.); niall.murray@tus.ie (N.M.); thiago.braga@tus.ie (T.B.R.)

**Keywords:** movement disorders, tremors, electromyography, MDS-UPDRS, healthy subjects, task based, low frequency signals, signal processing

## Abstract

Objective quantification of tremor remains a challenge in Parkinson’s disease (PD) assessment, with current clinical assessments relying largely on subjective scale ratings. This study evaluates the feasibility and signal behaviour of integrating surface electromyography (sEMG) with MDS-UPDRS-aligned tasks in a healthy adult cohort, with the aim of establishing normative low-frequency muscle activation profiles. Thirty-two healthy participants (mean age 27.6 ± 5.3 years) completed seven upper-limb tasks derived from the MDS-UPDRS while sEMG data were recorded from antagonistic forearm muscles. Signals were normalised using maximum voluntary contraction, filtered at 14 Hz, and analysed using frequency-domain (FFT) and time-frequency (STFT) methods. Significant task-dependent differences were observed in both frequency occurrence and magnitude (*p* < 0.05), particularly within the 3.5–9 Hz range. Finger tapping elicited increased low-frequency activity compared to baseline, while pronation–supination produced the most stable and consistent muscle activation across participants. Frequencies above 12 Hz showed minimal task discrimination. These findings demonstrate that low-frequency tremor-like activity can occur during specific MDS-UPDRS tasks in healthy individuals and may require further validation before being considered suitable for PD staging. This work establishes normative sEMG benchmarks to support future clinical validation and PD cohort comparisons.

## 1. Introduction

Parkinson’s disease (PD) is a progressive neurodegenerative disease characterised by a range of motor and non-motor symptoms, including bradykinesia, rigidity, postural instability, and tremor [1]. Tremor is often among the earliest and most recognisable motor manifestations and is typically described as an involuntary, rhythmic oscillation arising from alternating or synchronous muscle activation [2,3]. Despite its clinical relevance, tremor remains challenging to assess objectively, particularly in early or mild disease stages, where symptom presentation may be subtle and variable.

Diagnosis and management of PD is based on history and comprehensive assessments, using scales such as the MDS-UPDRS [4,5]. During these assessments, neurologists observe the patient during the performance of specific tasks and assigns scores based on standardised rating scales (e.g., 0–4 ratings). These scales evaluate diverse areas of PD, analysing disease stage, quality of life, activities of daily living, impairment, disability, and many other aspects [6].

Although computational tools have been introduced in clinical settings, their adoption remains inconsistent, and subjective scoring persists as the clinical gold standard. Inter-rate variability persists among different clinicians or medical centres conducting assessments [4,7]. With the development of sensing technologies, a variety of sensor-based devices have been used to assess PD. Among these, inertial measuring units (IMU), electromyography (EMG) [8], accelerometers [9], gyroscopes [10], and force sensors have been used frequently [11]. These sensors contain hardware and software to gather critical data (e.g., movement speed, rotation, muscle activity), which can be used to complement diagnosis or therapy activities. They overcome some of the limitations associated with the clinical evaluation scales in terms of objectivity, sensitivity, and accuracy [12]. However, challenges in accuracy may stem from task complexity or limitations in wearable sensor detection of low-frequency signals, and the focus on task-specific accuracy [13,14,15,16,17].

To bridge the substantial gap between subjective clinical assessments and objective quantification in PD diagnosis, this study introduces a novel application of surface EMG (sEMG) for monitoring hand movements during specific tasks outlined in the MDS-UPDRS. Quantitative tremor parameters have been explored in recent studies but are not yet standardised in clinical practice, highlighting the need for systematic low-frequency analysis [18,19,20].

This study represents a foundational stage in the development of an objective, sensor-driven PD assessment by integrating sEMG with the task structure of the MDS-UPDRS. The primary aim is to evaluate the feasibility, repeatability, and signal behaviour of sEMG during selected upper-limb tasks in a healthy adult cohort. By analysing task-dependent frequency and magnitude characteristics across predefined tremor-relevant bands, this work establishes a normative baseline that will support subsequent validation in clinically diagnosed PD populations. Importantly, no diagnostic or staging claims are made; rather, the focus is on methodological evaluation and baseline characterisation to inform future research.

## 2. Methodology

### 2.1. Study Design and Ethical Approval

This study employed a within-subject experimental design to evaluate task-dependent sEMG behaviour during MDS-UPDRS-aligned upper-limb tasks in a healthy adult cohort. Ethical approval was granted by the Technological University of the Shannon (TUS). All participants provided written informed consent prior to participation, and all data were anonymised prior to analysis.

### 2.2. Participants

Thirty-two healthy adults (16 female, 16 males, mean age 27.60 ± 5.34) were recruited using convenience sampling. Four subjects were removed during data cleansing. Gender balance guidelines were applied as recommended by the Parkinsons Foundations gender balance in research and care [21]. Participants experienced all tasks in a within-group design. The inclusion criteria included individuals aged 18 to 34 who were generally healthy. Exclusion criteria were based on related literature and included factors such as anxiety, insufficient sleep, alcohol and caffeine consumption, medication use, pregnancy or pregnancy symptoms, and involuntary shaking or tremor [22,23,24].

### 2.3. Instrumentation

EMG is a detection method of biomedical signals from electrical activity of muscle contractions [25]. It was chosen as EMG signals are often used to differentiate tremor in PD from other tremors, demonstrating its precision [26].

The wearable technology used was the Shimmer 3 EMG Unit [27]. It is designed for measuring physiological signals related to muscle contractions. The Shimmer EMG was chosen as it has a maximum sampling rate of 2048 Hz, is capable of capturing tremors within the 1 Hz to 10 Hz range, which is the frequency for PD, and has communication via Bluetooth for easy connectivity [28].

**Figure 1:**
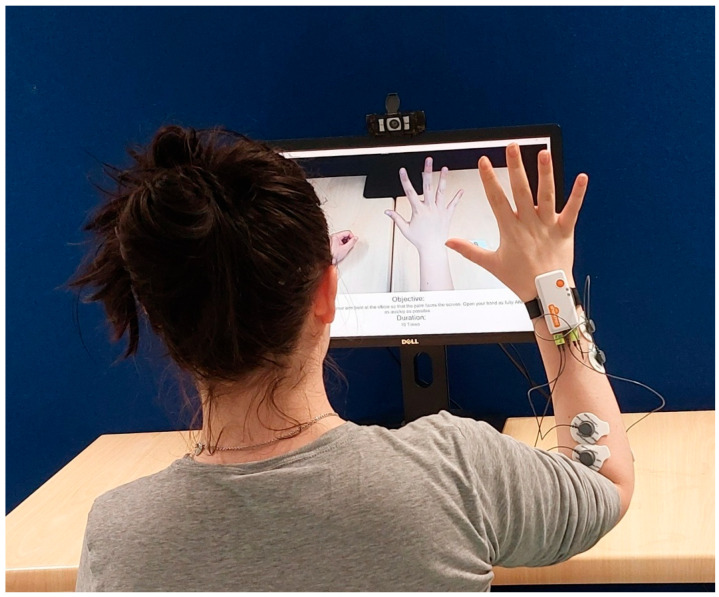
Shimmer placement on dominant hand.

The Shimmer device was placed on the back of the dominant hand of the participant to capture movement data, and the EMG electrodes were placed on the antagonistic muscles of the participants arm to record muscle activity during tasks as shown in Figure 1.

**Figure 2:**
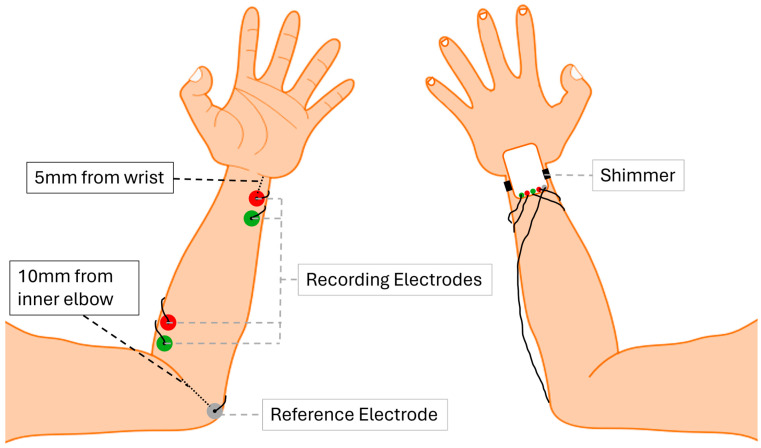
Sensor and electrode placement on forearm, frontal view of hand on the left, back view of the hand on the right.

The EMG electrodes utilised, Kendall H92SG [29], are single-use, medical-grade disposable pads (long-term use beyond 8 h not recommended). Each participant received a new set to prevent cross-contamination. The subject’s skin was carefully cleaned with a cotton alcohol wipe before electrode placement. Electrodes were placed on the extensor carpi ulnaris, roughly 5 mm above the wrist bone, and on the extensor carpi radialis roughly 10 mm from the inner elbow. The reference electrodes were placed 10 mm laterally from the recording electrodes. Placements of electrodes are illustrated in Figure 2. Accuracy was verified through Nyquist sampling validation, sensor calibration, repeat-trial reliability (intra-subject consistency), and comparison with known tremor frequency bands (3–12 Hz).

### 2.4. Task Protocol

**Figure 3:**
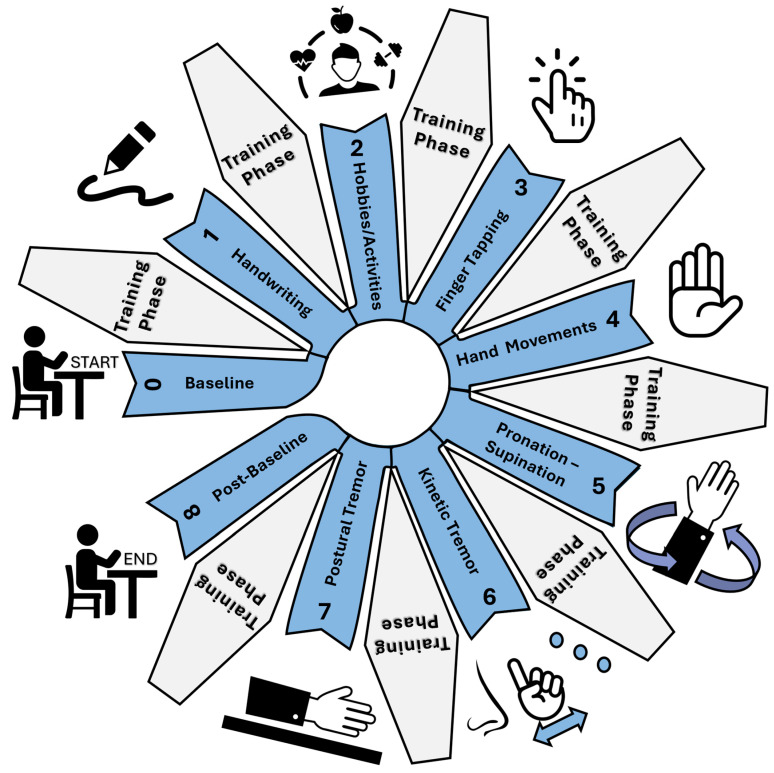
Training and testing phases.

Participants performed a series of seven upper-limb tasks adapted from Parts II and III of the MDS-UPDRS, selected based on their relevance to hand movement, tremor manifestation, and motor control. Each task, shown in Figure 3, was selected not merely for replication of the MDS-UPDRS, but for its potential to elicit specific motor patterns (e.g., tremor, slowness, or amplitude reduction) in a controlled setting.

- Handwriting: Adapted from MDS-UPDRS (Task 2.7). In the scale, this question is assessed through the participants’ experience over the past week of people having difficulties reading their handwriting. The choice to include this question as a quantitative task was based on the literature [30,31] regarding improving this evaluation as a motor task rather than a subjective question.
- Hobbies/activities: Adapted from MDS-UPDRS Part III (Task 3.3). To aim is to examine rigidity during movement. The participant was required to move an object from a position on their left-hand side, to a position on their right-hand side.
- Finger tapping: A combination of elements from MDS-UPDRS Part III (Task 3.3 and 3.4). Task 3.3’s objective was to examine rigidity, which is a slow passive movement of major joints, an activation manoeuvre that is recommended if no rigidity is detected, which is where Task 3.4 is integrated. Task 3.4 is where the participant taps their index finger on their thumb 10 times as quickly and as fully as possible.
- Hand movements: A replication of MDS-UPDRS Part III (Task 3.5). The participant is instructed to make a tight fist with the arm bent at the elbow so that the palm faces the examiner. The participant will open the hand 10 times as fully and as quickly as possible.
- Pronation–supination: Task 5 is a replication of Task 3.6 from Part III. The participant should extend their arm out in front of their body with the palms facing down, then turn the palm up and down alternately 10 times as fast and fully as possible.
- Kinetic tremor: Task 6 is adapted from Task 3.16 from Part III. This is tested by a finger-to-nose manoeuvre. With the arm starting in an outstretched position, the participant should perform at least 3 finger-to-nose manoeuvres. To minimise variability with the tasks during testing, a static object with three points horizontally placed was used in place of an examiner’s finger. The participant was to touch finger to nose from left to right.
- Postural tremor: Task 7 is a replication of Task 3.15 from Part III. The participant should stretch the arms out in front of the body with palms facing down, the wrist should be straight, and fingers comfortably spread.
- Baseline and post-baseline: Both baselines are adapted from Task 3.17 and 3.18 of the MDS-UPDRS. The participant should sit quietly in a chair with hands placed on the arm of the chair, and their feet comfortably supported on the floor for 10 s with no other directives.

Task 3.18 from Part III of the MDS-UPDRS, which focuses on the constancy of rest tremors during the examination period, was continuously assessed while other tasks were being performed.

All tasks were performed using a standardised protocol delivered through a custom software interface incorporating synchronised video and text instructions. This approach ensured consistent task execution timing, minimised examiner influence, and reduced inter-participant variability. Rest/training periods were incorporated between tasks to minimise fatigue effects.

### 2.5. Signal Pre-Processing

**Figure 4:**
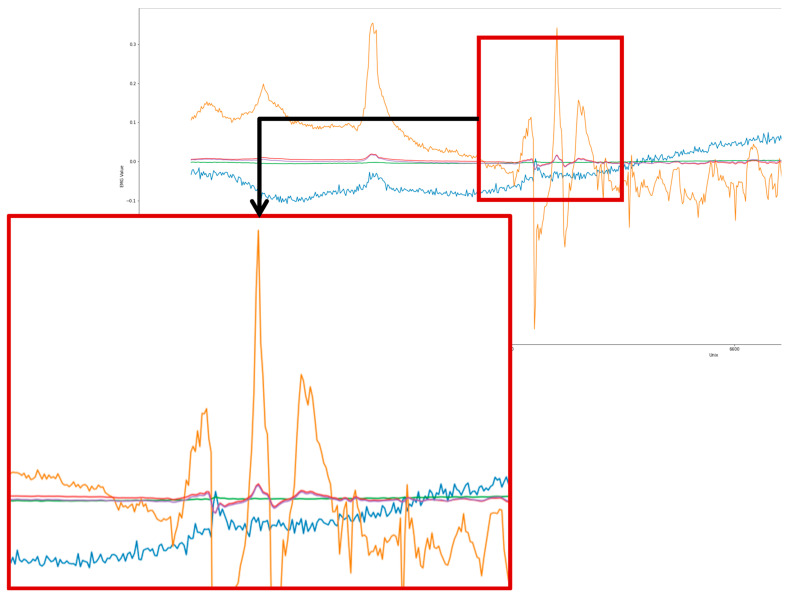
SEMG signals; The blue and orange lines represent the raw EMG signal; the red and green lines represent the filtered EMG signals. The purple line is the combined signals filtered.

sEMG signals were pre-processed in several stages. Firstly, all data were split based on task type, excluding data collected between tasks. Next, both baseline stages were trimmed to 3 min, removing the initial and final segments for a more accurate capture with less noise [32]. Thirdly, the sEMG raw signals (Figure 4) for all tasks were normalised using the maximum voluntary contraction (MVC); the value for this was taken from Task 4 (hand movement—gripping an object) data for each participant. A 10th order Butterworth filter [33] with a cut-off frequency of 14 Hz was applied to retain low-frequency signals for analysis. This cut-off aligns with PD tremor types occurring within the 1–12 Hz range, with an additional 2 Hz so that signals were not cut-off [34,35,36,37].

**Figure 5:**
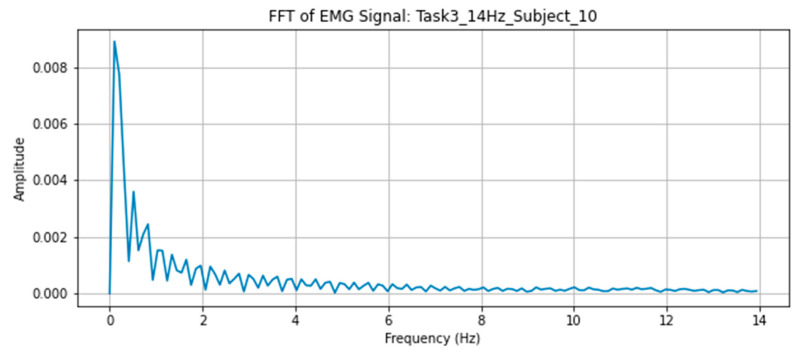
FFT of the EMG signal.

Fast Fourier Transform (FFT) [38] was applied to the EMG signals to extract the amplitude of the frequency components during tasks as shown in Figure 5.

To enhance frequency identification during tasks, we used Short-Time Fourier Transform (STFT) [39]. Although STFT has limitations in time-frequency resolutions for non-stationary signals, the Gaussian window size was adapted to match the task duration; for example, baseline was longer than task duration, meaning the window size was bigger for the baseline. The number of samples per segment (nperseg) influenced the frequency analysis resolution, which was calculated according to (Equation (A1)) in the Appendix A [40].

The STFT data were used to create spectrograms. The spectrograms served multiple purposes, including assessing fatigue, muscle activation patterns, task comparisons, and identifying potential artifacts or noise in the EMG signals.

### 2.6. Statistical Analysis

Data analysis was performed in SPSS (Version 29.0.0) [41]. A Kolmogorov–Smirnov [42] test was performed on the EMG data during the tasks, to test whether the data were normally distributed. Each task came back statistically significant (*p* < 0.05) (Table 1 and Table 2). Non-parametric tests were then applied.

A Kruskal–Wallis Test [43] was then conducted to assess the differences across tasks given the non-parametric nature of the data. A pairwise comparison was conducted on the EMG data, revealing a statistically significant difference (*p* < 0.05) and was associated with small to moderate effect sizes (ε^2^ ≈ 0.05) for each task.

The next step was to split the EMG data per task into frequency groups to determine which frequencies repeated or had a higher occurrence per task. The data were split into five frequency bands, based on the range of frequencies typical for PD groups [44,45]: noise (<3.5 Hz), rest frequencies (RFG) (≥3.5 Hz and ≤5.9 Hz) [46], action frequencies (AFG) (≥6.0 Hz and ≤8.9 Hz) [47], postural frequencies (PFG) (≥9.0 Hz and ≤12.0 Hz) [48], and normal range (NFG) (12.0+ Hz) [49]. While action tremor can be related to other diseases, the combination of this with a prevalence of resting tremor is an indication that it is PD [50]. Postural tremor is much harder to distinguish from other diseases, but is still a tremor type in PD, and the literature suggests there are two types [51]: essential tremor and pure postural tremor. Pure postural tremor (associated with PD) is not associated with dystonic symptoms that are commonly seen with essential tremors. The noise and normal groups were not considered during this study as they behaved as expected based on the literature [52].

A normality test (Kolmogorov–Smirnov) was conducted for each frequency group over both the frequency and the magnitude, in which all groups came back significant (*p* < 0.05), associated with small to moderate effect sizes (ε^2^ ≈ 0.05). Consequently, the Kruskal–Wallis test was applied on the tasks for comparison with the data from the STFTs.

## 3. Results

Pairwise comparisons (Kruskal–Wallis) across the RFG and AFG revealed significant differences in task performances (as detailed in Table 1 and Table 2). Where results per frequency group are presented, significant differences were identified in task pairs with both the Bonferroni-adjusted *p*-value (Adj. Sig) and unadjusted *p*-value (Sig) being less than 0.05. For clarification, due to the repeated occurrence below, Task 0 is baseline, Task 8 is post-baseline, Task 3 is finger tapping, and Task 6 is pronation and supination of the arms.

In the Kruskal–Wallis analysis, the test statistic reflects the difference of means across frequency groups for each task. Specifically, this non-parametric method ranks the data and evaluates whether the median task performance significantly differs between the groups.

### 3.1. Rest Frequency (RFG) (3.5–6 Hz)

Pairwise comparisons of tasks are based on the RFG highlighted notable differences among the tasks. The analysis, shown in Table 1, demonstrates significant differences between Task 0 and Task 8 when compared with Task 3 (*p* < 0.05).

**Figure 6:**
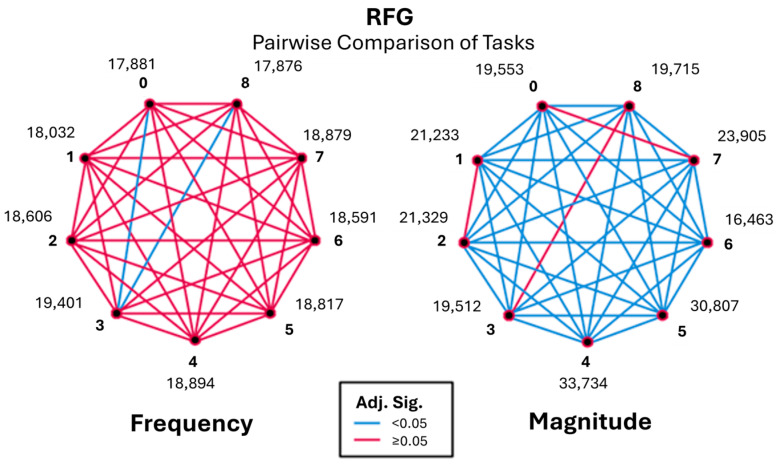
Pairwise visualisation of frequency and magnitude for the rest frequency group (RFG).

These observations support the idea that tasks 0, 3, and 8 are significant in terms of frequency characteristics, therefore in need of further investigation. To address this, the magnitude of all tasks was explored. Figure 6 represents pairwise comparisons between baseline, post-baseline, and all other tasks with the frequency of EMG signals in the range of 3.5–6 Hz.

Pairwise comparisons revealed that finger tapping (Task 3) differed significantly from baseline (Task 0) and post-baseline (Task 8), exhibiting a higher occurrence of low-frequency components during task execution. In contrast, baseline and post-baseline conditions did not differ significantly, demonstrating consistent resting behaviour before and after task performance.

To further investigate these tasks, STFT spectrograms were used. It is important to note at the outset that the inter-subject variations for tasks 0 and 8 are not captured by the test statistic, which primarily measures the difference between the sample average ranks of tasks. This limitation highlights that the test statistic provides a group-level comparison rather than individual-level variability, and subtle differences in muscle activity across subjects may remain unrepresented in statistics alone.

**Figure 7:**
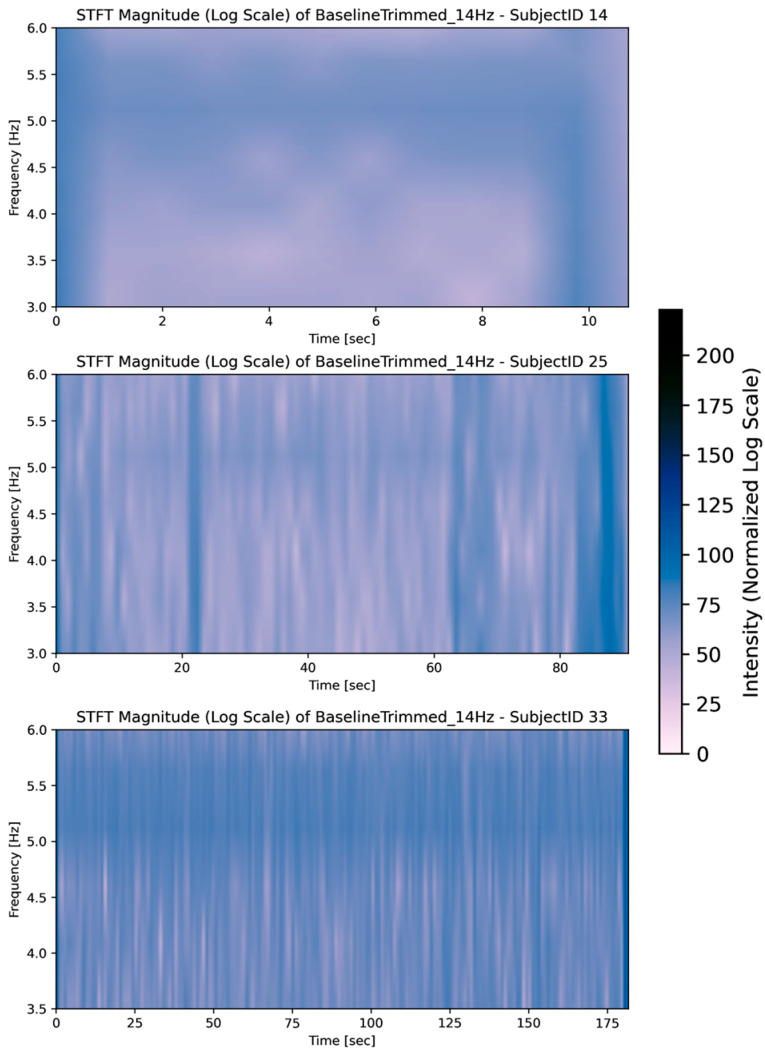
Rest frequencies during Task 0 (baseline).

Figure 7 visualises that Task 0 generally contains low-intensity muscle activity, showing many non-significant differences in the frequency domain, even among varying time snippets of the task (image 1: 10 s, image 2: 90 s, image 3: 180 s). This suggests that muscle activation patterns during Task 0 are relatively consistent across different subjects, which is expected for a task with minimal activity.

The variations in intensity suggest differences in muscle engagement across the group. While baseline patterns appeared relatively consistent, certain tasks exhibited significant variability in magnitude activity, reflecting task-specific responses within the cohort.

**Figure 8:**
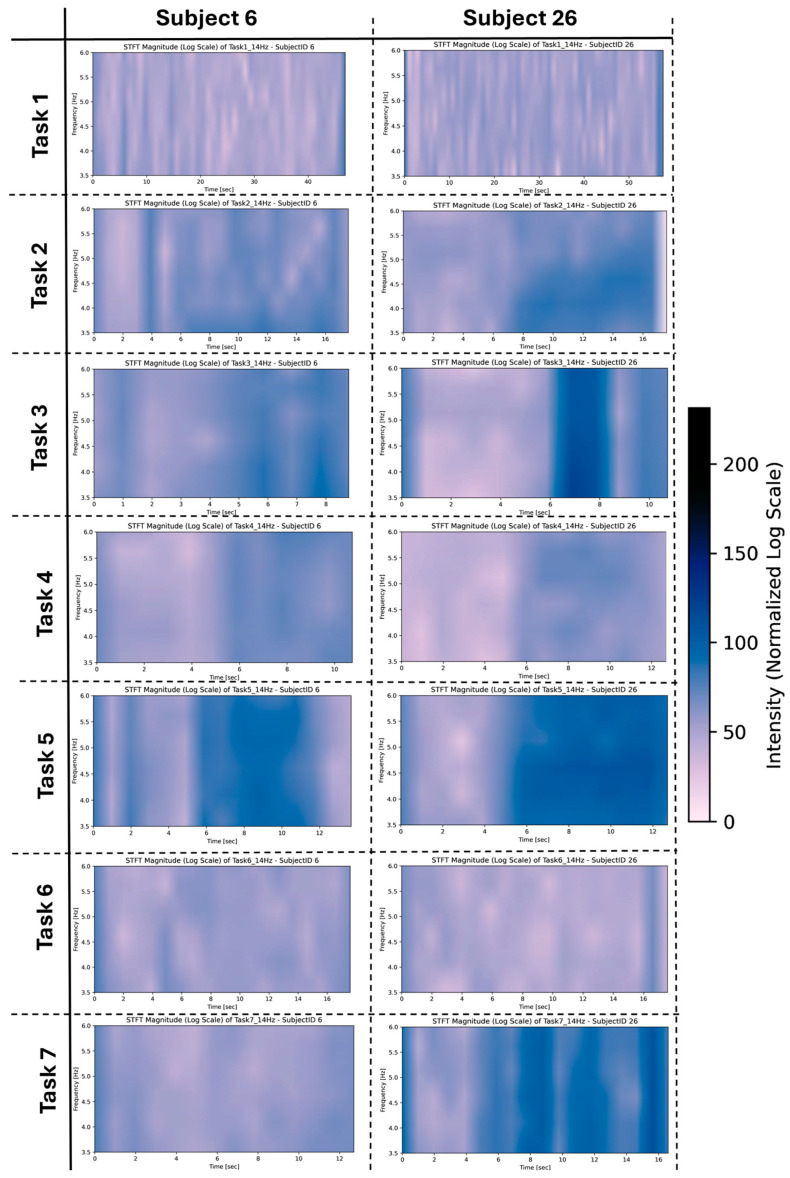
Rest frequencies for subjects 6 and 26 for tasks 1–7.

For example, in Figure 8, the STFTs for tasks 1–7 demonstrate variations in magnitude activity across tasks, such as increased activity in tasks 3, 5, and 7. These fluctuations highlight group-level trends in task-specific responses, contributing to the significant pairwise comparisons observed in Figure 6.

It is important to note that these STFTs represent only a subset of the group’s performance, offering an initial perspective on the data. A broader analysis of all tasks and participants was conducted to identify broader patterns and better understand the observed variability at the group level; these STFTs are available as Supplementary Materials.

**Figure 9:**
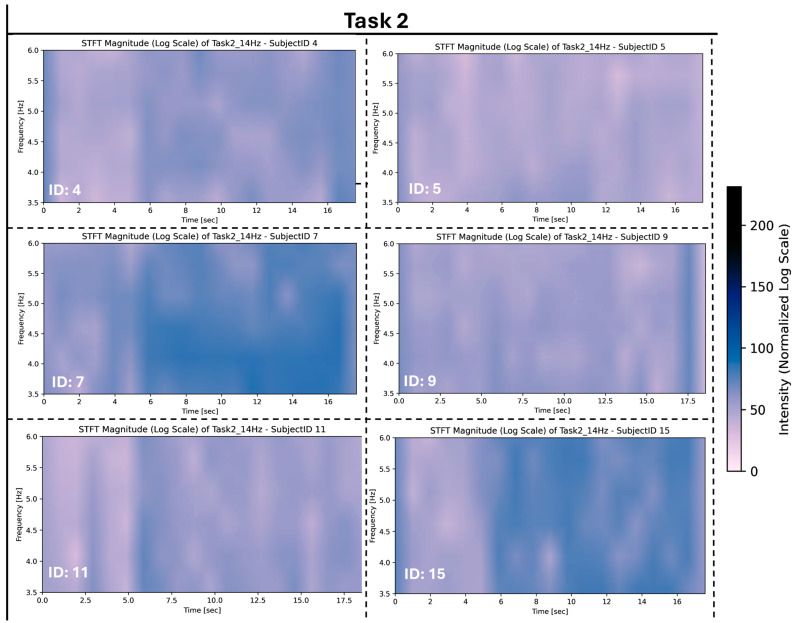
Task 2 STFT rest frequency for varying subjects.

Figure 9 illustrates the group-level variation observed during Task 2, revealing notable differences in frequency intensity and distribution across participants. While certain patterns emerge, such as heightened intensity in the 3.5–6 Hz frequency range, other responses are characterised by more uniform or lower-intensity activity.

These differences highlight variability in the signal characteristics during Task 2, suggesting diverse frequency responses within the group. This variability may reflect underlying physiological differences or variations in task-related engagement patterns across the cohort.

**Figure 10:**
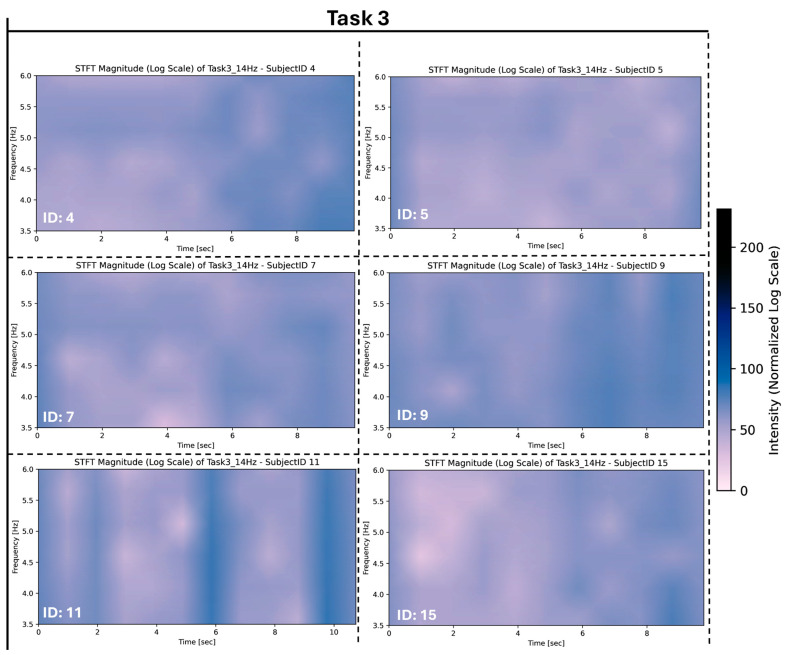
Task 3 STFT rest frequency for varying subjects.

These variations were observed for tasks 1, 2, 4, 5, and 7. These variations were more prominent among subjects for Task 3, as evident in Figure 10 where stronger frequency intensities were measured for each subject for this task compared to the other tasks they performed.

**Figure 11:**
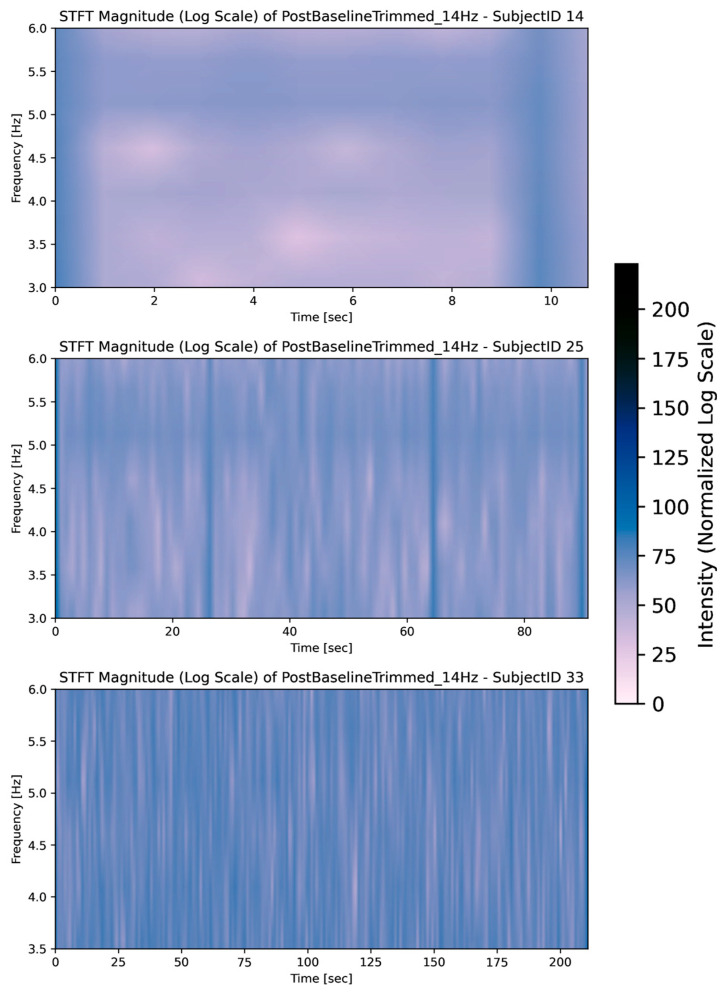
Rest frequencies of Task 8.

The STFTs for Task 8, given in Figure 11, shows similar frequency compared to Task 0, but with increased intensity observed in most subjects, aligning with the pairwise results. The presence of intermittent frequencies and strong magnitudes in certain tasks highlights that healthy muscles can also exhibit complex low-frequency patterns. This means that detecting PD-specific tremors or dyskinesias requires distinguishing these patterns from normal intermittent activation.

Tasks 0, 3, and 8 emerged as distinct in terms of frequency characteristics, with significant differences observed in pairwise comparisons. Task 3 demonstrated the highest frequency of tremors compared to tasks 0 and 8, likely due to task-specific motor demands, while Task 8 exhibited significantly lower magnitudes, reflecting a recovery phase following prior tasks. Task 0, as a baseline, showed the most consistent muscle activity with minimal variability across subjects.

The analysis also revealed variability in task-specific responses within the group. Group-level trends showed that tasks 3, 5, and 7 elicited stronger magnitude activity, whereas Task 6 demonstrated the lowest magnitudes. These variations suggest that both task-specific motor demands and physiological differences contribute to the observed patterns.

In summary, for the 3.5–6 Hz frequency band, Task 3 exhibited notable variations across participants, reflecting diverse engagement patterns. Task 8’s spectrograms aligned with its pairwise results, showing similarities to baseline (Task 0) but with increased intensity potentially due to accumulated fatigue from prior tasks.

### 3.2. Action Frequency (AFG) (6–9 Hz)

The action frequency group was examined to assess task-dependent muscle activity within frequencies commonly associated with voluntary and kinetic movements. Kruskal–Wallis analysis demonstrated significant task-related differences in magnitude (*p* < 0.05), while differences in frequency occurrence were less pronounced. Effect sizes were small to moderate (ε^2^ ≈ 0.05), indicating that task execution contributed meaningfully to observed variability in muscle activation intensity.

**Figure 12:**
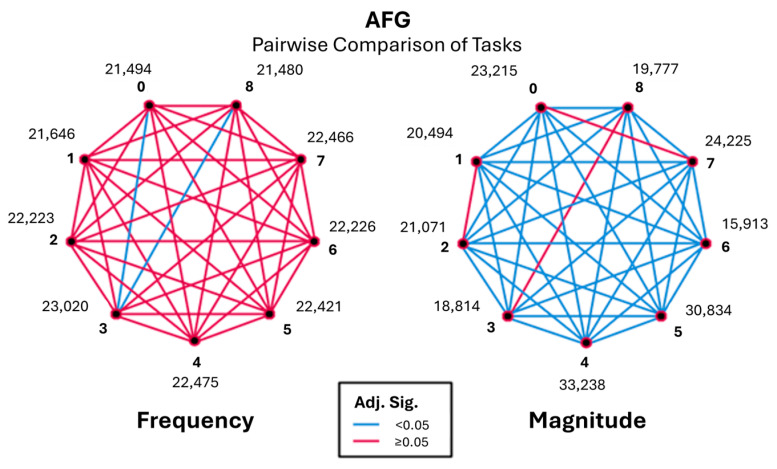
Pairwise visualisation of frequency and magnitude for the action frequency group (AFG).

Pairwise comparisons, as shown in Figure 12, showed that Task 3 again differed significantly from baseline and post-baseline, with increased magnitude values during task performance. Task 6 maintained consistently low magnitudes across comparisons, reinforcing its stability across frequency bands. Baseline and post-baseline conditions remained statistically similar, confirming their suitability as non-active reference conditions.

STFT analysis revealed consistent low-intensity activity during baseline conditions, with similar dynamic temporal behaviour observed during active tasks. Several tasks exhibited progressive increases in frequency toward later portions of task execution.

Overall, the action frequency range exhibited task-dependent patterns that closely mirrored those observed in the rest frequency group, with magnitude-based measures providing clearer differentiation between tasks than frequency occurrence alone.

### 3.3. Postural Frequencies (PFG) (9–12 Hz)

**Figure 13:**
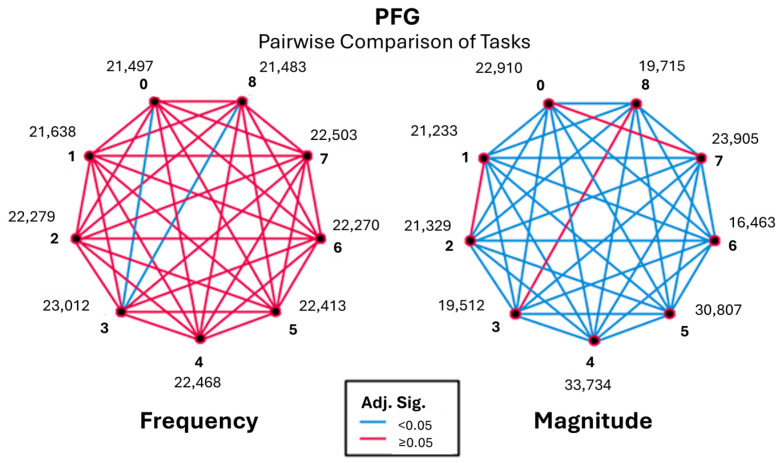
Pairwise visualisation of frequency and magnitude for the postural frequency group (PFG).

Analysis of sEMG signals within the 9–12 Hz range revealed fewer and weaker task-dependent differences compared to the lower frequency groups. Significant differences in frequency occurrence were primarily observed for Task 3 relative to baseline (Task 0) and post-baseline (Task 8), as shown in Figure 13 and Supplementary Materials. Tasks 0 and 8 exhibited nearly identical rank positions, indicating comparable frequency occurrence and consistent behaviour under baseline and recovery conditions.

Task 6 demonstrates significant and varying degrees of difference with nearly all tasks, particularly showing a stronger frequency occurrence when compared to tasks such as Task 0 and Task 4, with very high standard test statistics (18.243 and 33.726, respectively) as shown in Table 1. PFG follows a similar trend of frequencies to those seen in RFG and AFG.

Overall, the postural frequency range exhibited reduced variability and weaker task discrimination compared to the rest and action frequency groups. While task-related differences were detectable, they were modest in magnitude and largely uniform across participants. These findings suggest that the 9–12 Hz range captures steadier physiological processes and contributes less to task differentiation than lower-frequency bands, reinforcing the importance of analysing multiple frequency ranges to fully characterise task-dependent muscle behaviour.

### 3.4. Normal Frequencies (NFG) (12+ Hz)

**Figure 14:**
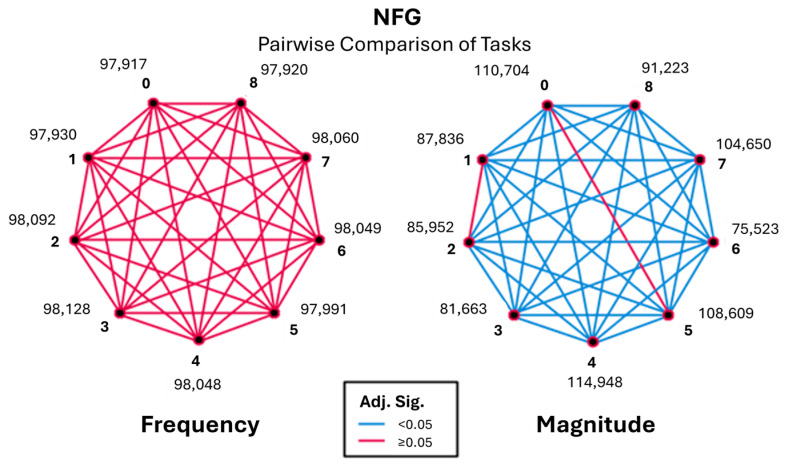
Pairwise visualisation of frequency and magnitude for the normal frequency group (NFG).

Frequencies above 12 Hz demonstrated minimal task discrimination and consistently low magnitudes across all conditions. Neither frequency nor magnitude provided meaningful differentiation between tasks as visible in Figure 14. As a result, this range was considered non-informative for task-based tremor profiling in healthy participants and was excluded from further inferential interpretation.

The findings support the premise that focusing on lower frequency ranges (e.g., 3–9 Hz) is optimal for profiling tremors, as these ranges show more significant variability and muscle activation patterns that are task specific.

Finger tapping consistently elicited increased low-frequency muscle activation compared to baseline conditions, whereas pronation–supination demonstrated the most stable and lowest-magnitude responses across frequency ranges. Baseline and post-baseline conditions exhibited highly consistent behaviour throughout all analyses, supporting their use as reliable reference states.

## 4. Discussion

This study highlights the ability of specific tasks to differentiate muscle activation patterns within various frequency ranges, notably the 3.5–9 Hz range. However, the analysis revealed significant differences in muscle activation during Task 3 (finger-tapping task) when compared to tasks 0 (baseline) and 8 (post-baseline), within a healthy subject population.

The pronounced tremor frequencies elicited by Task 3 in frequencies 3–9 Hz in a healthy population raise concerns regarding its suitability for diagnostic purposes, particularly within the context of subjective use of the MDS-UPDRS. Given this task’s propensity to induce ‘tremor-like’ frequencies among those without PD, relying on it subjectively for clinical assessments could result in ineffective staging of the disease. This suggests a need for cautious interpretation of results derived from Task 3. Integrating more objective measurement tools to complement its use during clinical evaluations or selecting alternative tasks that more accurately reflect pathological muscle activity would be more suitable. This approach could mitigate the risk of misinterpretation and ensure that diagnostic tools are both sensitive and specific to the pathophysiological traits of the disease.

Notably, Task 6 consistently demonstrated the lowest frequency and magnitude of muscle activity, marking it as an ideal metric for evaluating the neuromuscular function of healthy subjects. The task elicits relatively uniform muscle activation, which suggests it effectively mirrors the expected muscle activity in a healthy population. For patients suspected of having neuromuscular disorders, Task 6 could assist in distinguishing pathological tremors or abnormal movements from normal physiological responses.

Establishing normative EMG profiles may enable pathological tremor activity to be distinguished from task-induced physiological tremor, improving the specificity of PD assessment. To further explore and validate the effectiveness of these tasks, objective evaluations involving both healthy populations and clinical populations are essential. Such studies would help indicate the normal ranges of muscle activity for various tasks and establish more definitive baselines for pathological deviations. This approach ensures a more comprehensive understanding of task-specific muscle responses and enhances the diagnostic accuracy of neuromuscular assessments.

### Limitations

Several limitations of this study should be acknowledged. First, the absence of a PD or clinical comparison group prevents any conclusions regarding pathological tremor discrimination or clinical applicability. This study was intentionally designed as a foundational investigation to characterise task-dependent low-frequency sEMG behaviour in healthy individuals. Establishing normative reference patterns is a necessary methodological step prior to clinical validation, as low-frequency activity can arise from voluntary motor execution, physiological tremor, and inter-individual variability. Without a clear understanding of healthy task behaviour, subsequent comparisons with PD cohorts risk misinterpretation of task-induced activity as pathological.

Second, the sample size was relatively modest, which may limit statistical power and generalisability. However, the cohort size was sufficient to identify consistent task-dependent trends and variability patterns across frequency bands, which aligns with the exploratory and baseline-focused aims of this study. Larger cohorts will be required in future work to strengthen statistical robustness and support subgroup analyses.

Third, participants were restricted to a narrow age range (18–34 years). This was a deliberate design choice to capture the healthiest segment of the adult population and minimise the likelihood of undiagnosed neurological conditions, including prodromal Parkinson’s disease. Constraining age-related variability allowed clearer interpretation of task-induced muscle activity and reduced confounding effects associated with ageing-related neuromuscular changes.

Finally, inter-session and test–retest reliability were not assessed. While this limits conclusions regarding longitudinal stability, the single-session design was intentional to ensure naïve task performance. Repeated testing may introduce learning effects, altered motor strategies, or performance optimisation, potentially biasing baseline measurements. Assessment of reliability and repeatability will be addressed in future studies once normative task behaviour has been established and extended to clinical populations.

## 5. Conclusions

This study’s findings demonstrate that while certain tasks—such as finger tapping—can elicit tremor-like low-frequency activity in healthy individuals, others, including pronation and supination, produce stable and consistent muscle activation profiles. These results provide critical insight into which tasks are most suitable for distinguishing normal motor behaviour from potential pathological patterns and highlight the importance of standardising movement conditions when interpreting EMG data. The proposed protocol contributes a reproducible baseline for multimodal fusion and cross-validation across sensor types.

Importantly, this work does not attempt to make clinical inferences about Parkinson’s disease directly but rather lays the methodological groundwork for subsequent validation with diagnosed PD participants. Future research will focus on recruiting experts and clinically diagnosed PD participants to evaluate the system’s discriminative power and potential for longitudinal monitoring across MDS-UPDRS assessments.

By advancing a structured, sensor-integrated approach to MDS-UPDRS task execution, this study contributes to the development of a more objective, data-driven foundation for future PD evaluation. The establishment of normative EMG profiles under controlled conditions represents a critical step toward reducing subjectivity in clinical assessments and improving the reliability of future diagnostic and monitoring tools.

## Figures and Tables

**Table 1:** Significant results from pairwise comparisons of frequency in groups.

Group	Sample 1-Sample 2	Test Statistic	Std. Error	Std. Test Statistic	Sig.	Adj. Sig.
Rest	8-3	5.436	129	0.042	0.966	1.000
Rest	0-3	−1520	427	−3.553	<0.001	0.014
Rest	8-6	714	320	2.227	0.026	0.935
Action	8-3	1540	467	3.297	<0.001	0.035
Action	0-3	−1526	468	−3.258	0.001	0.040
Action	0-6	−733	353	−2.074	0.038	1.000
Action	8-6	746	351	2.123	0.034	1.000
Postural	1-6	−632	379	−1.666	0.096	1.000
Postural	6-2	9.506	476	0.020	0.984	1.000
Postural	0-6	−773	353	−2.188	0.029	1.000
Postural	6-7	−233	505	−0.461	0.645	1.000
Postural	6-5	143	504	0.284	0.776	1.000
Postural	6-4	197	512	0.386	0.699	1.000

**Table 2:** Significant results from pairwise comparisons of magnitude in groups.

Group	Sample 1-Sample 2	Test Statistic	Std. Error	Std. Test Statistic	Sig.	Adj. Sig.
Rest	3-8	−752	426	−1.764	0.078	1.000
Rest	3-0	3737	427	8.735	<0.001	0.000
Rest	6-8	−3651	320	−11.377	<0.001	0.000
Action	3-8	−962	467	−2.059	0.039	1.000
Action	3-0	4400	468	9.390	<0.001	0.000
Action	6-0	7301	353	20.666	<0.001	0.000
Action	6-8	−3863	351	−10.992	<0.001	0.000
Postural	6-1	4769	379	12.567	<0.001	0.000
Postural	6-2	4865	476	10.220	<0.001	0.000
Postural	6-0	6446	353	18.243	<0.001	0.000
Postural	6-7	−7442	505	−14.714	<0.001	0.000
Postural	6-5	14,343	504	28.410	<0.001	0.000
Postural	6-4	17,271	512	33.726	<0.001	0.000

## Data Availability

The original contributions presented in the study are included in the article; further inquiries can be directed to the corresponding author.

## Supplementary Materials

The following supporting information can be downloaded at: https://www.mdpi.com/article/10.3390/s26010157/s1.

## Abbreviations

The following abbreviations are used in this manuscript:

PD	Parkinson’s Disease
MDS-UPDRS	Movement Disorder Society—Unified Parkinson’s Disease Rating Scale
sEMG	Surface Electromyography
IMU	Inertial Measurement Units

## Appendix A

### STFT Equations

(A1)
$$n_{\mathrm{perseg}}=\lfloor fs\cdot \left(time_{\mathrm{per}\;\mathrm{segment}\;\mathrm{ms}}\cdot 0.001\right)$$

The number of samples per segment varied based on whether a baseline or a task was being performed. The sampling frequency was multiplied by the time per task segment. This calculation was 3000 ms for baseline, or 2000 ms per task. The overlap was at 75% for each *n*_perseg_. The STFT was then performed using (Equation (A2)):

(A2) $$STFT\left[k,m\right]=\sum_{n=0}^{n_{\mathrm{perseg}}-1}x\left[n+m\right]\cdot w\left[n\right]\cdot e^{-j2\pi \frac{kn}{n_{\mathrm{perseg}}}}$$

where

*STFT*[*k*,*m*] represents the *STFT* at a specific frequency ‘*k*’ and the timeframe ‘*m*’.

*n*_parseg_ − 1 represents the number of samples in the analysis window minus one. *n*_parseg_ (number of points per segment) defines the size of the window in terms of discrete samples. Subtracting one gives the last index of the window.

x(n+m) ‘*x*’ represents the signal at a sample point of ‘*n* + *m*’, i.e., shifting the signal by ‘*m*’ samples over time.

w[n] is the window function being applied to the signal; it is applied to each sample ‘*n*’.

$e^{-j2\pi \frac{kn}{n_{\mathrm{perseg}}}}$ represents the conversion of the time-domain signal into the frequency domain.
