# Supplementary figures and images for: Electromyography (EMG) Signal Processing to Evaluate Low-Frequency Tremors
Source: Sensors (Basel). 2025 Dec 25;26(1):157. doi: 10.3390/s26010157 (PMC12788120; doi:10.3390/s26010157)

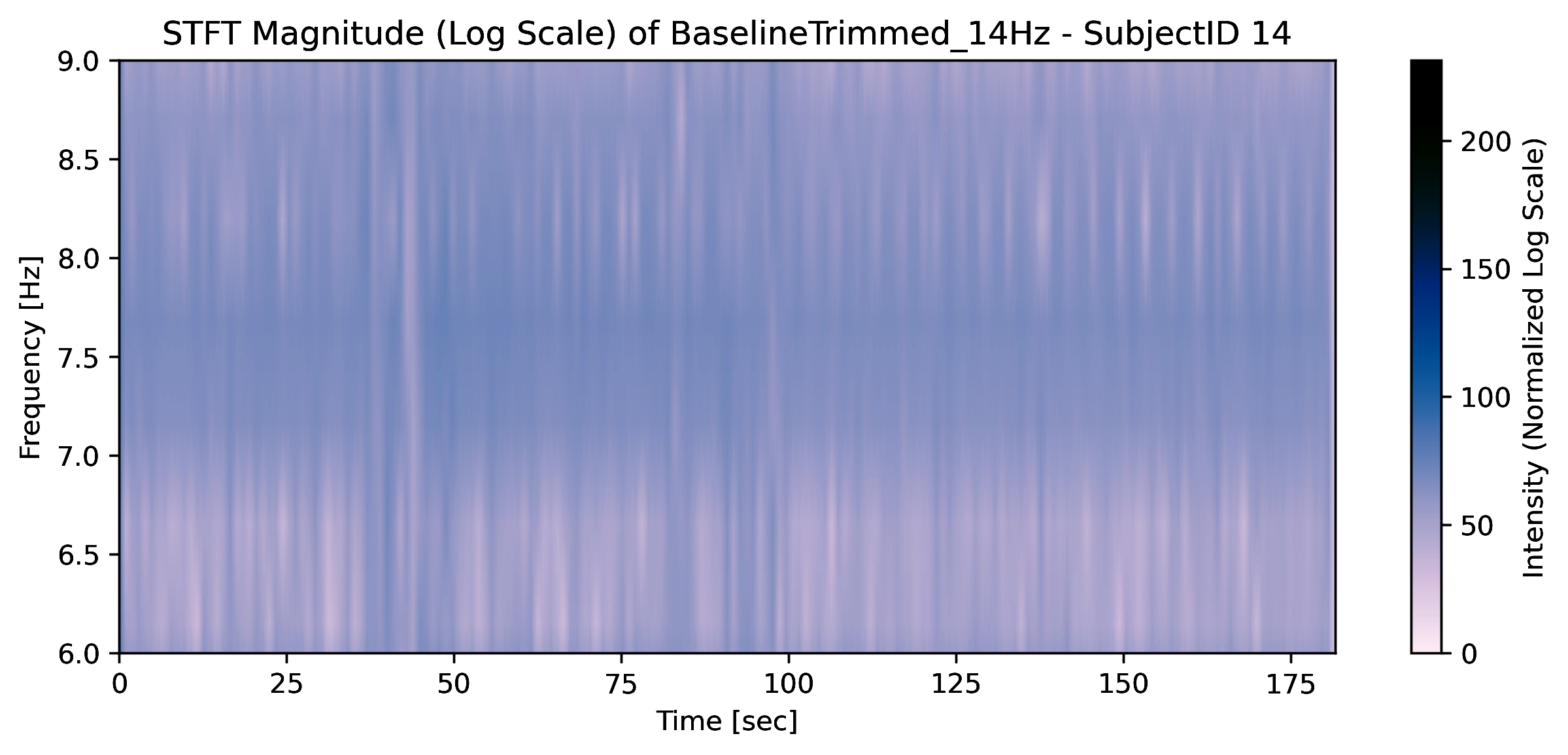

Supplement: Supplementary file 1 [file sensors-26-00157-s001.zip › STFT Images/AFG Images/Baseline Images/S1A Baseline ID_14.png]

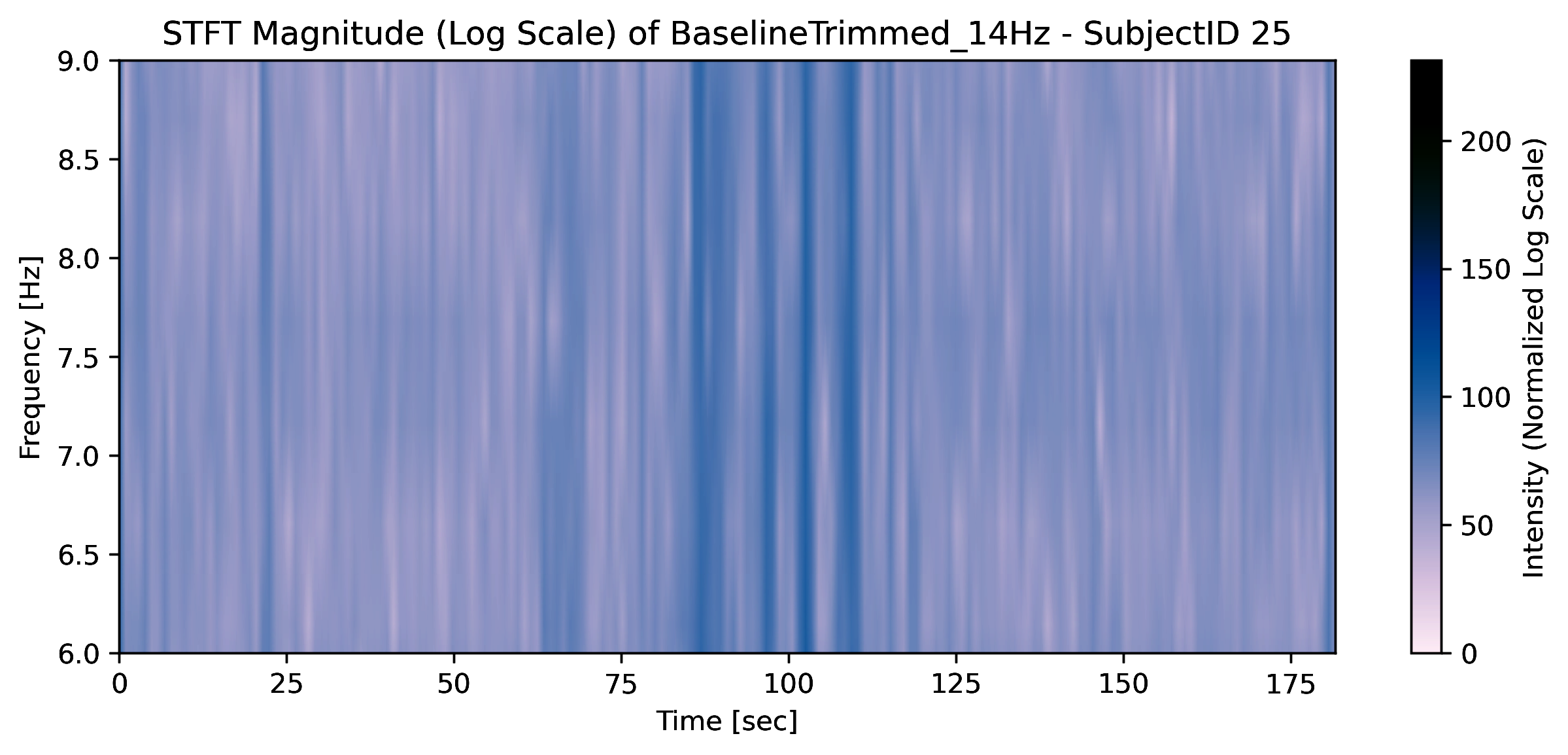

Supplement: Supplementary file 1 [file sensors-26-00157-s001.zip › STFT Images/AFG Images/Baseline Images/S1B Baseline ID_25.png]

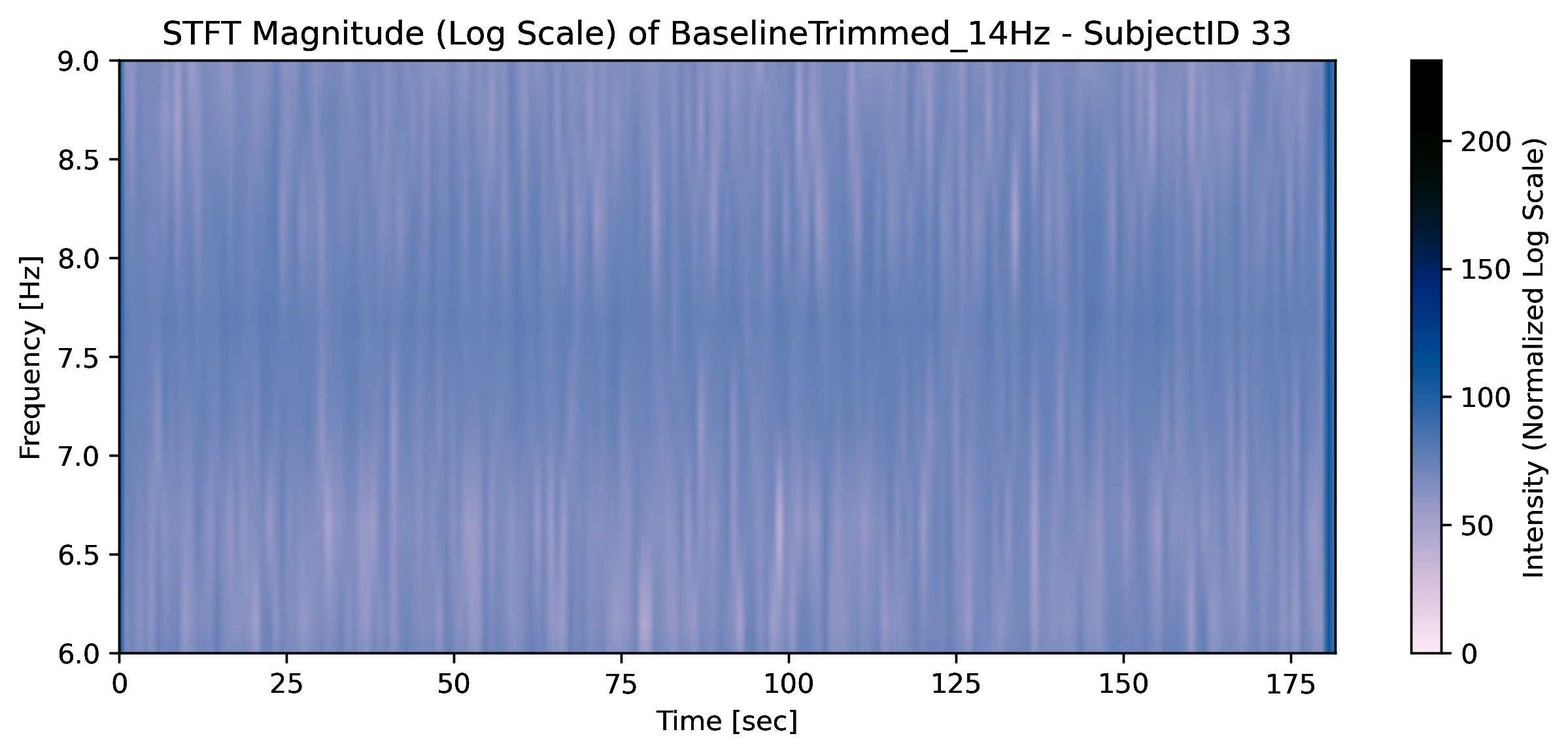

Supplement: Supplementary file 1 [file sensors-26-00157-s001.zip › STFT Images/AFG Images/Baseline Images/S1C Baseline ID_33.png]

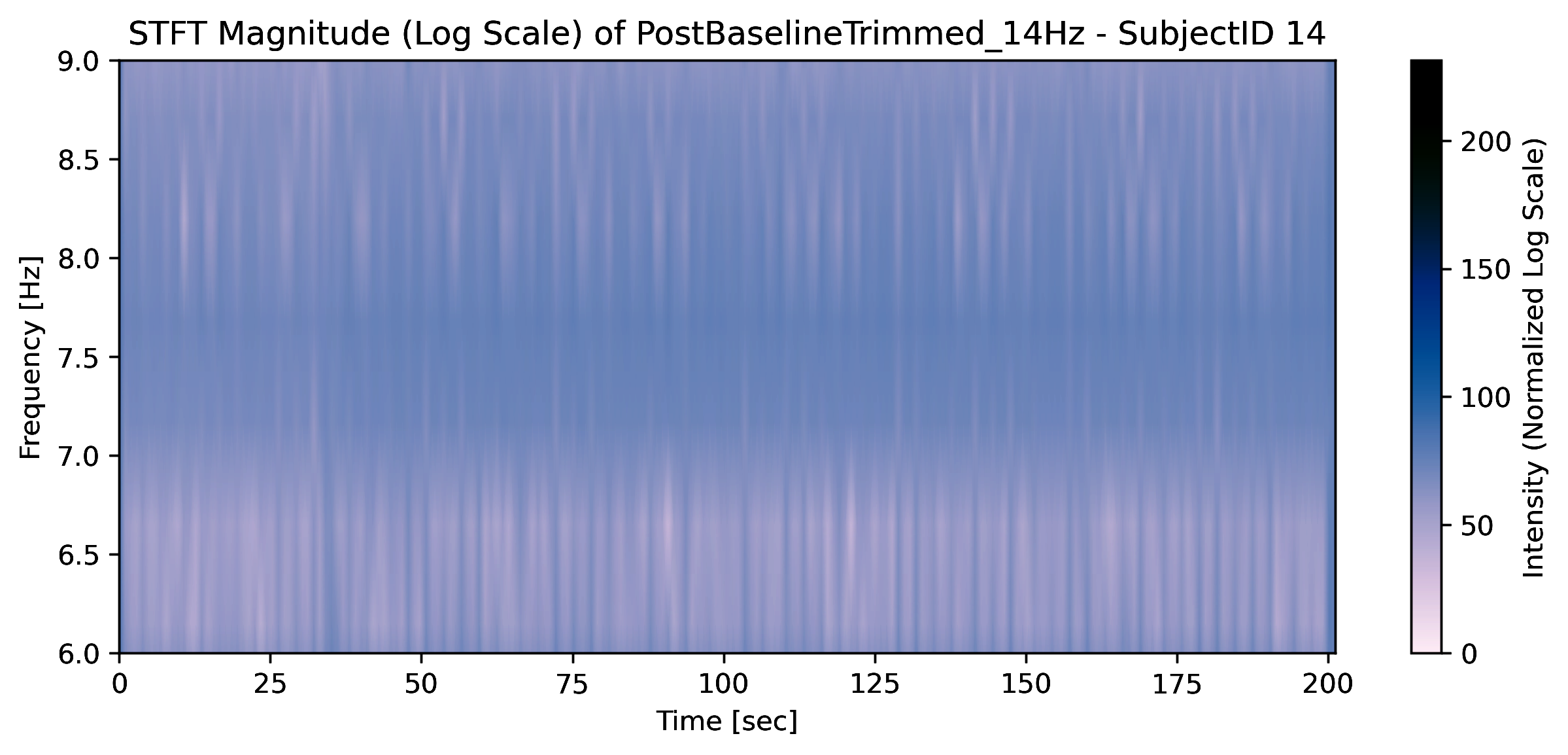

Supplement: Supplementary file 1 [file sensors-26-00157-s001.zip › STFT Images/AFG Images/PostBaseline Images/S2A ID_14_.png]

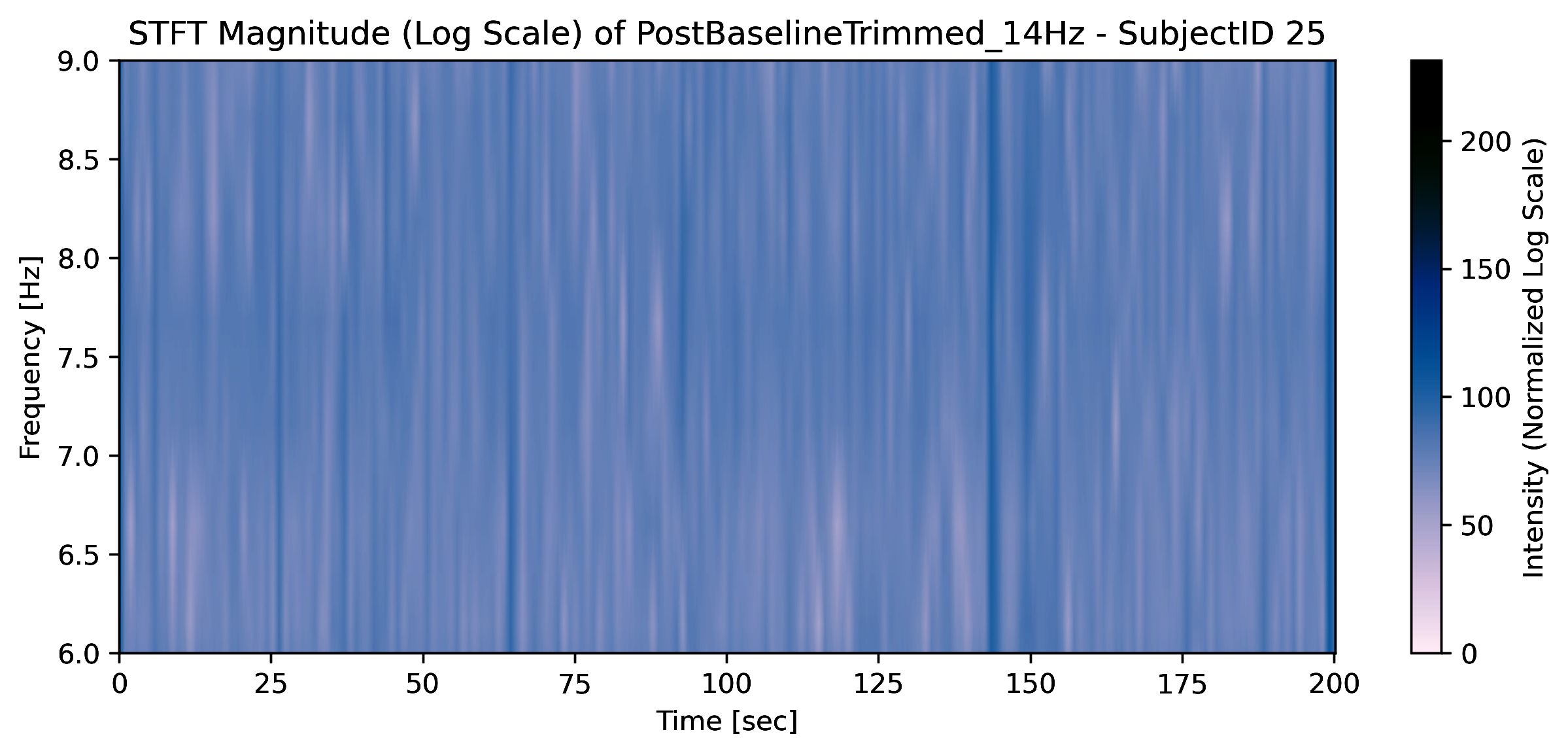

Supplement: Supplementary file 1 [file sensors-26-00157-s001.zip › STFT Images/AFG Images/PostBaseline Images/S2B ID_25.png]

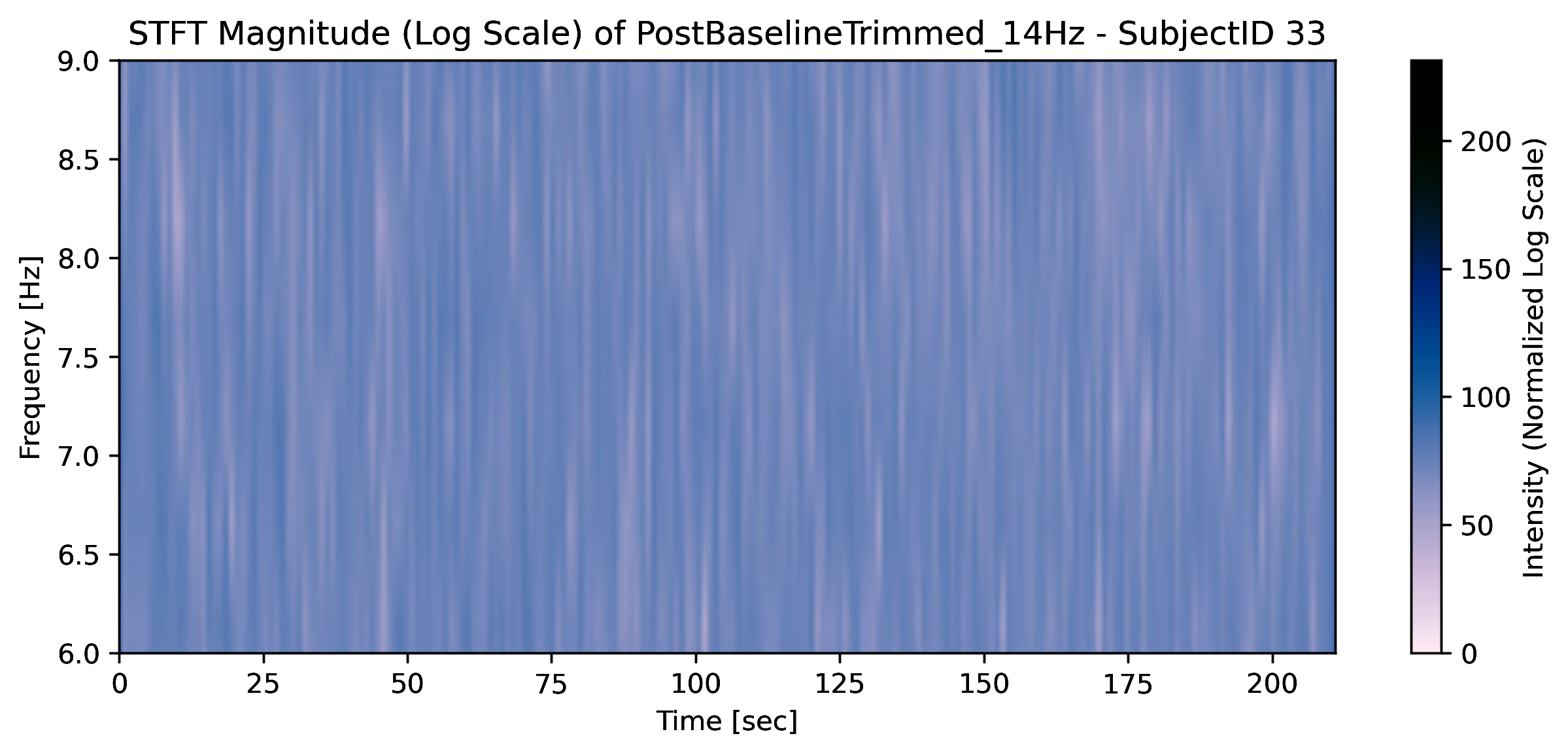

Supplement: Supplementary file 1 [file sensors-26-00157-s001.zip › STFT Images/AFG Images/PostBaseline Images/S2C ID_33.png]

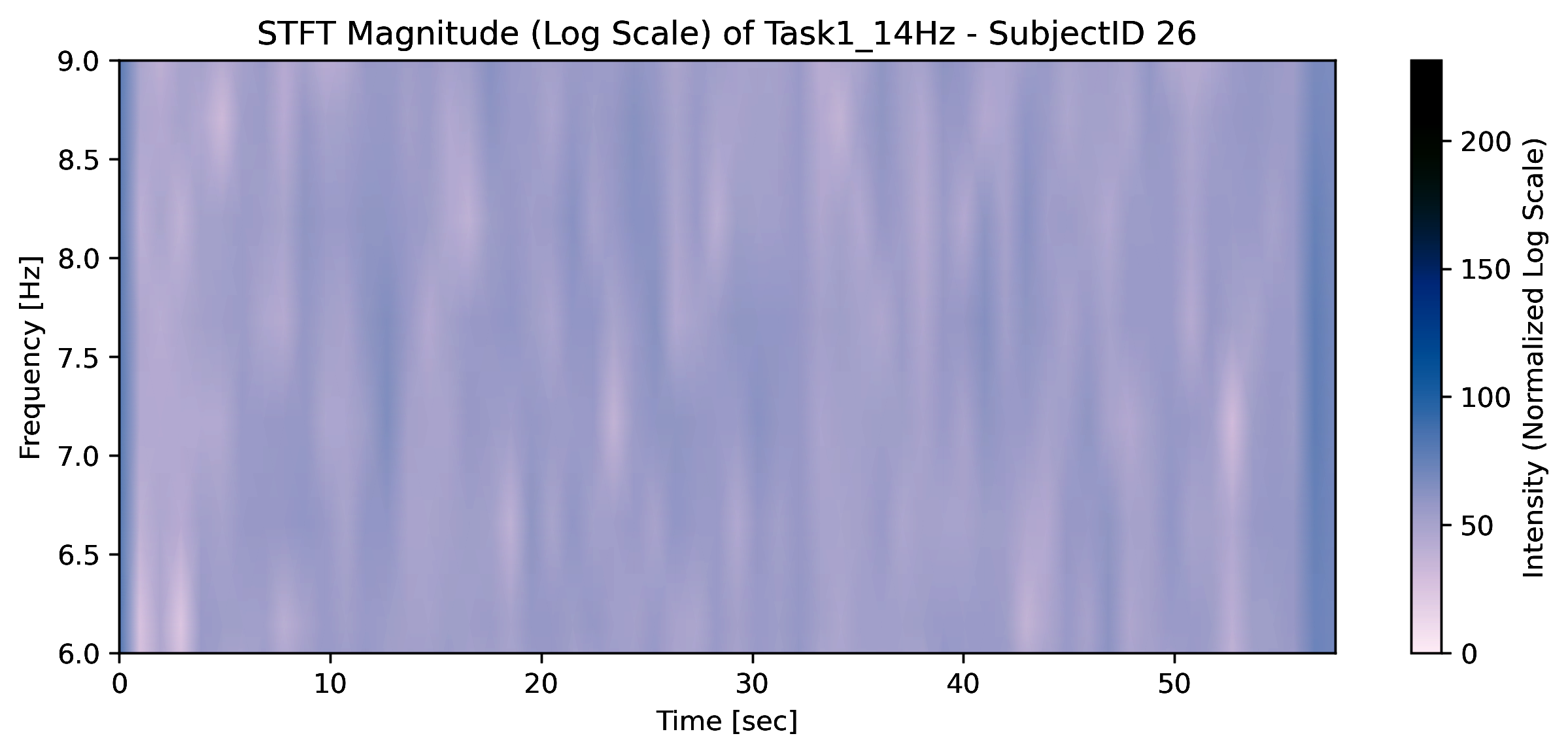

Supplement: Supplementary file 1 [file sensors-26-00157-s001.zip › STFT Images/AFG Images/Task 1-7 Images/S5 Task 1 D_26.png]

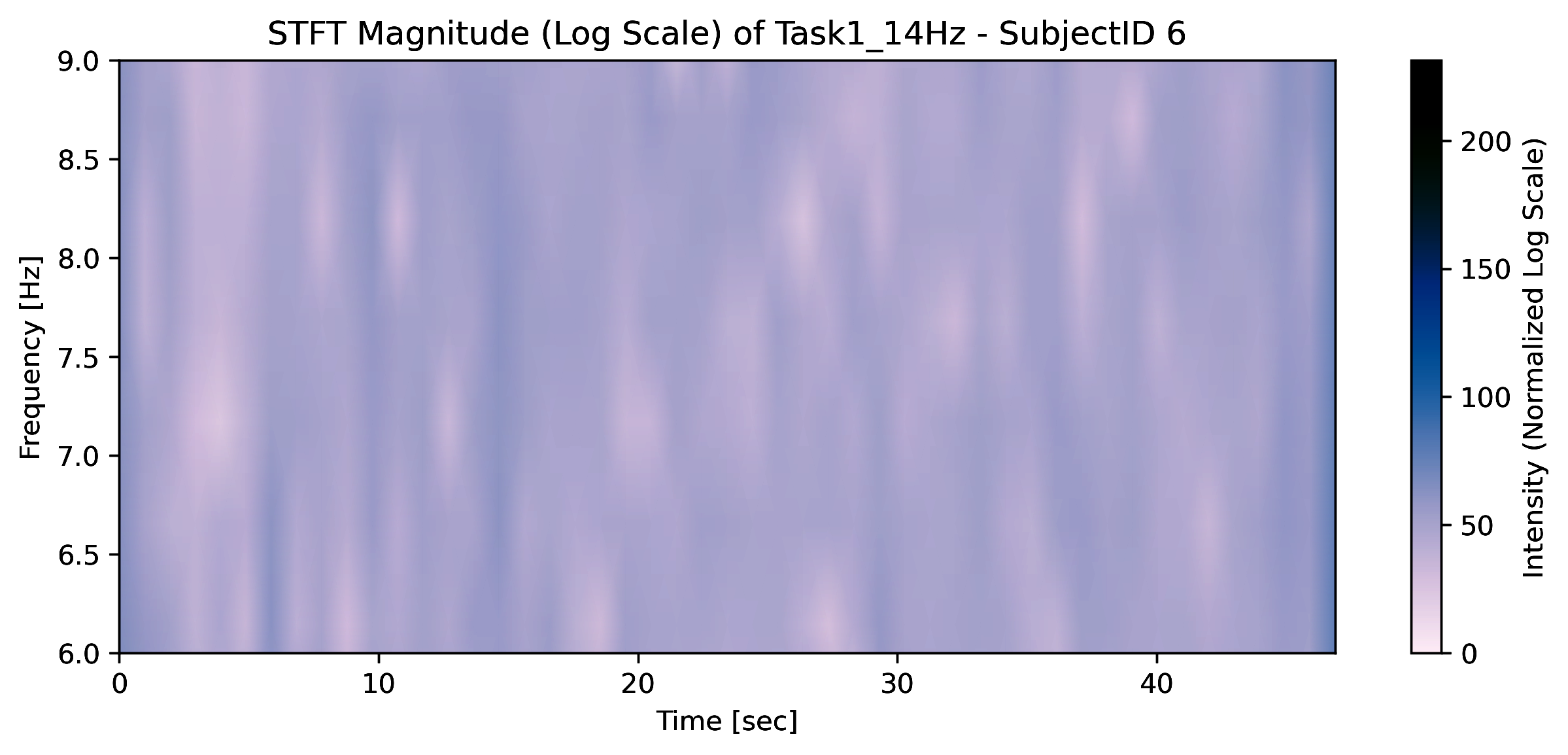

Supplement: Supplementary file 1 [file sensors-26-00157-s001.zip › STFT Images/AFG Images/Task 1-7 Images/S5 Task 1 ID_6.png]

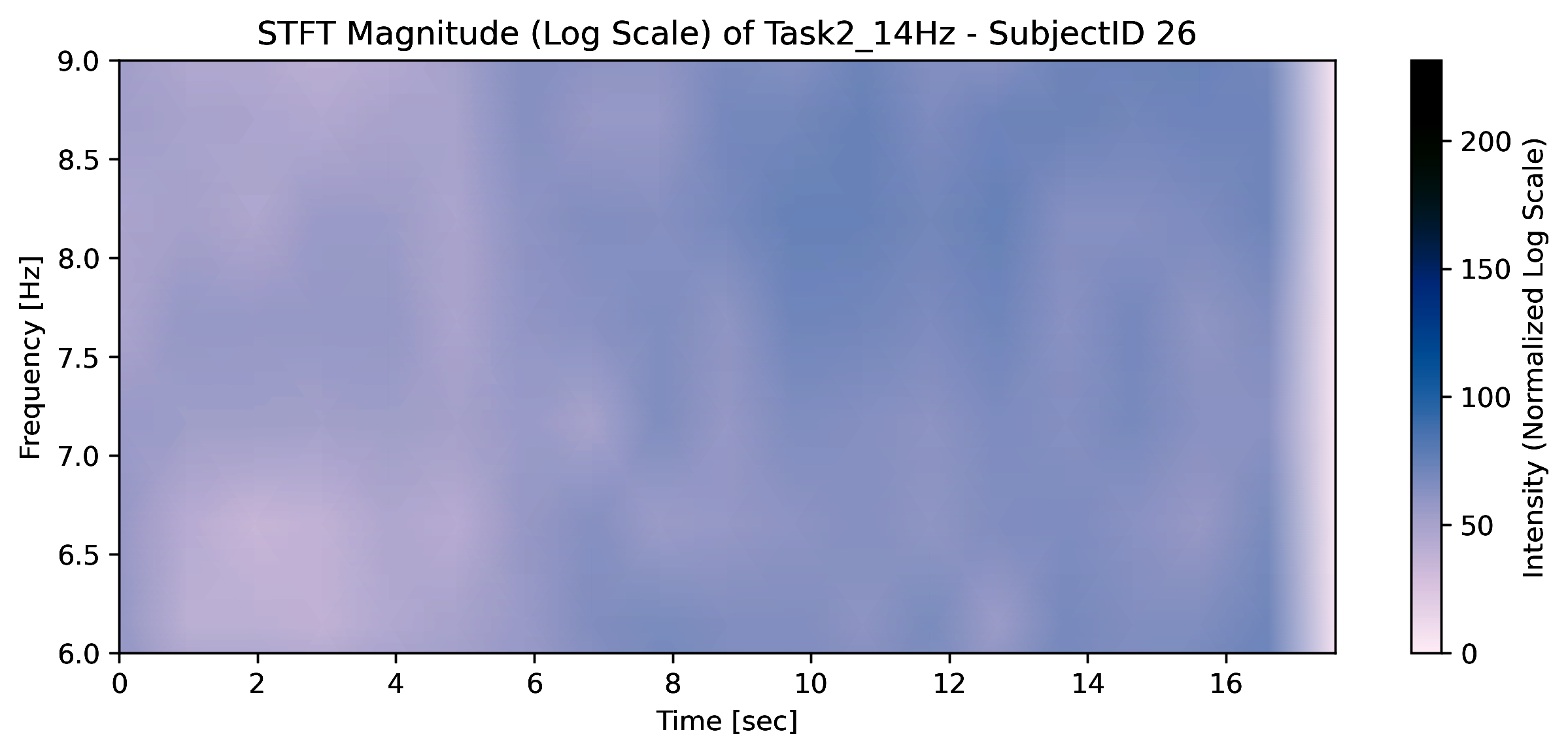

Supplement: Supplementary file 1 [file sensors-26-00157-s001.zip › STFT Images/AFG Images/Task 1-7 Images/S5 Task 2 ID_26.png]

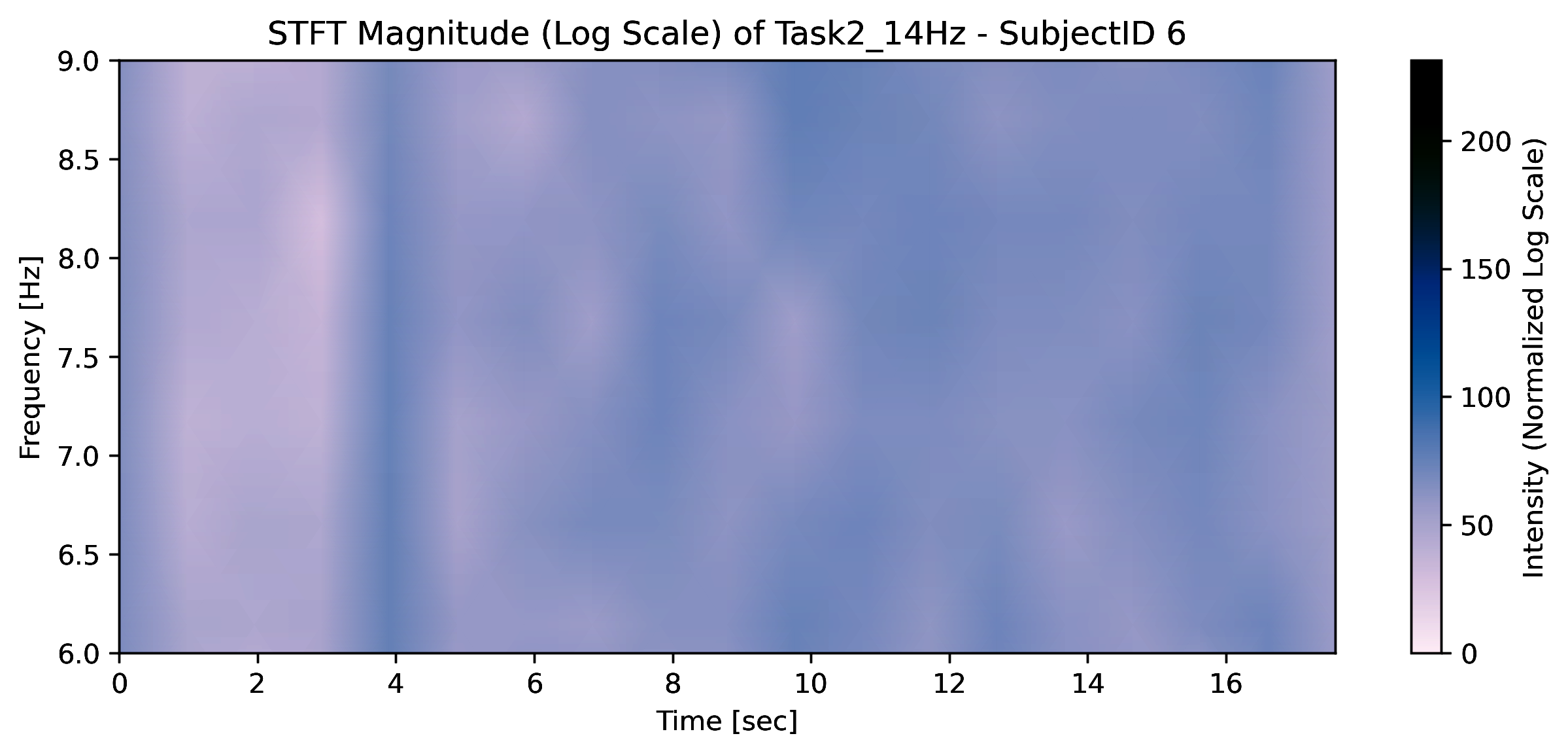

Supplement: Supplementary file 1 [file sensors-26-00157-s001.zip › STFT Images/AFG Images/Task 1-7 Images/S5 Task 2 ID_6.png]

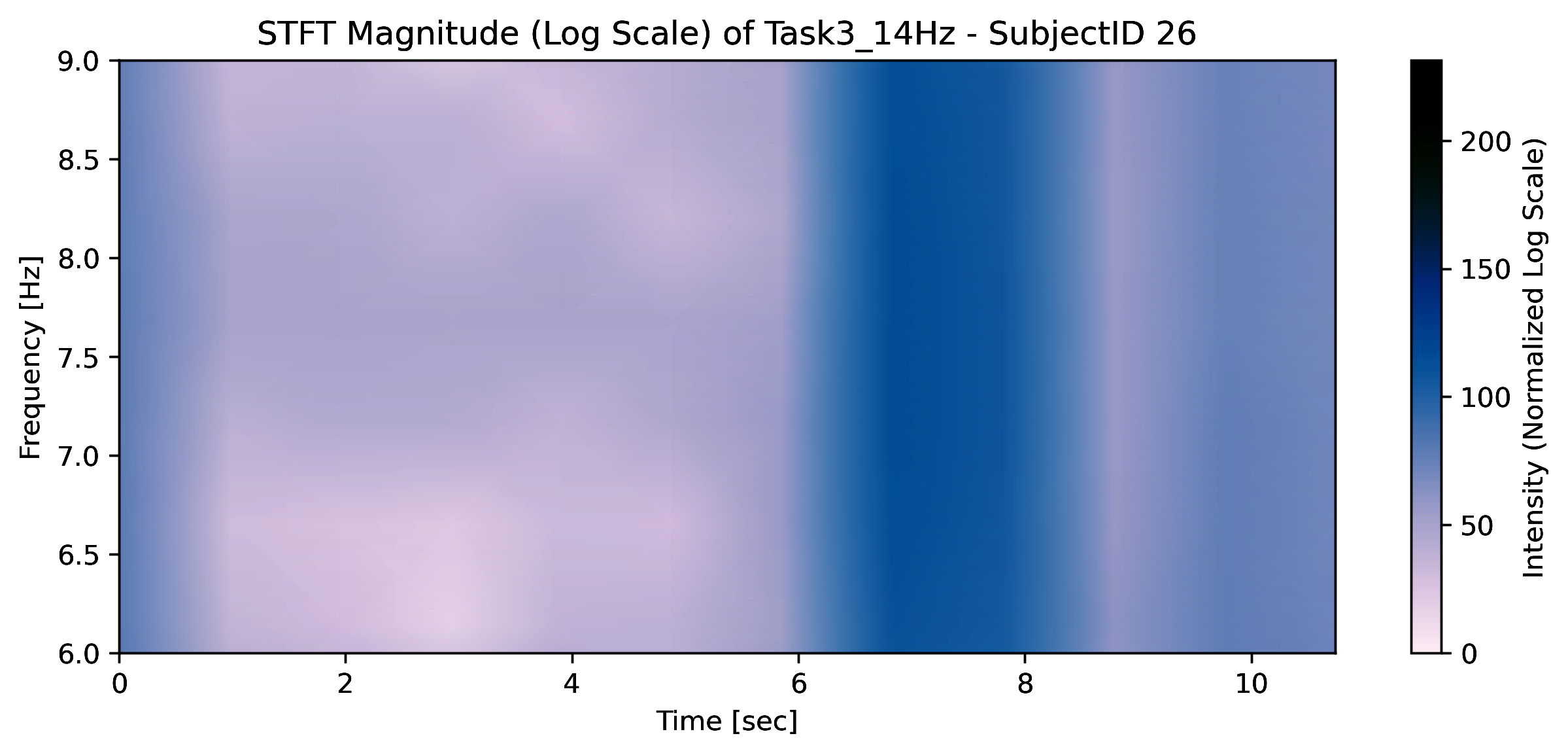

Supplement: Supplementary file 1 [file sensors-26-00157-s001.zip › STFT Images/AFG Images/Task 1-7 Images/S5 Task 3 ID_26.png]

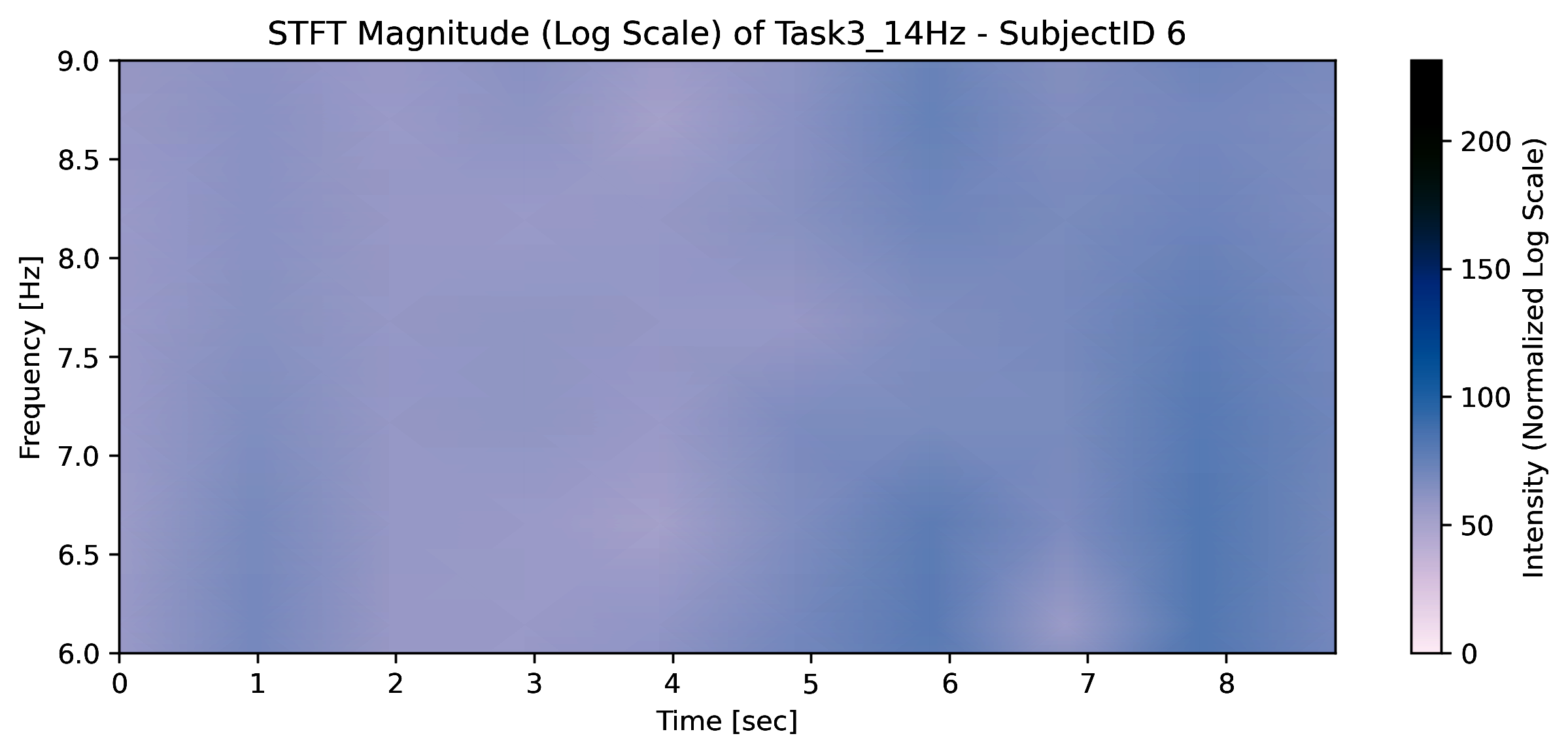

Supplement: Supplementary file 1 [file sensors-26-00157-s001.zip › STFT Images/AFG Images/Task 1-7 Images/S5 Task 3 ID_6.png]

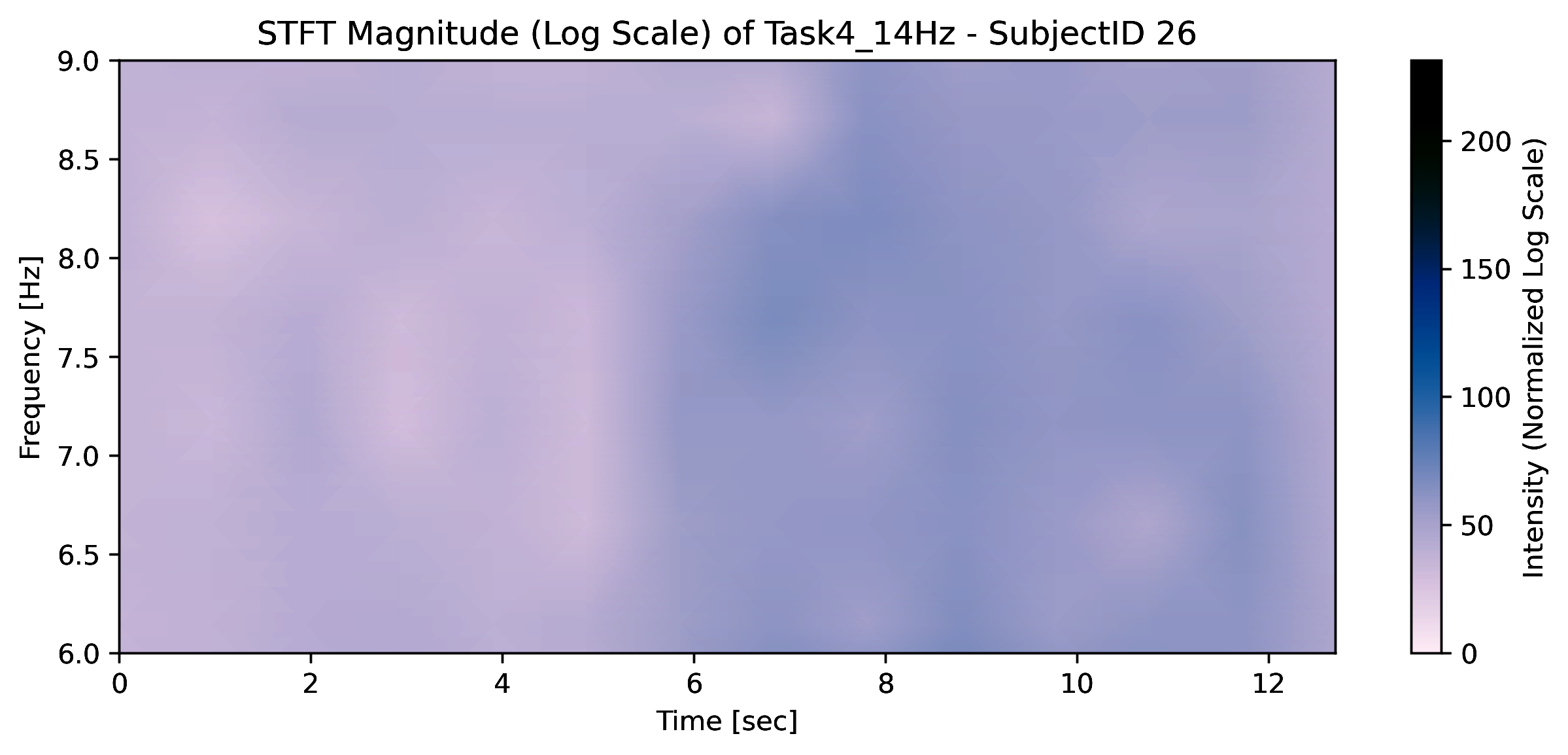

Supplement: Supplementary file 1 [file sensors-26-00157-s001.zip › STFT Images/AFG Images/Task 1-7 Images/S5 Task 4 ID_26.png]

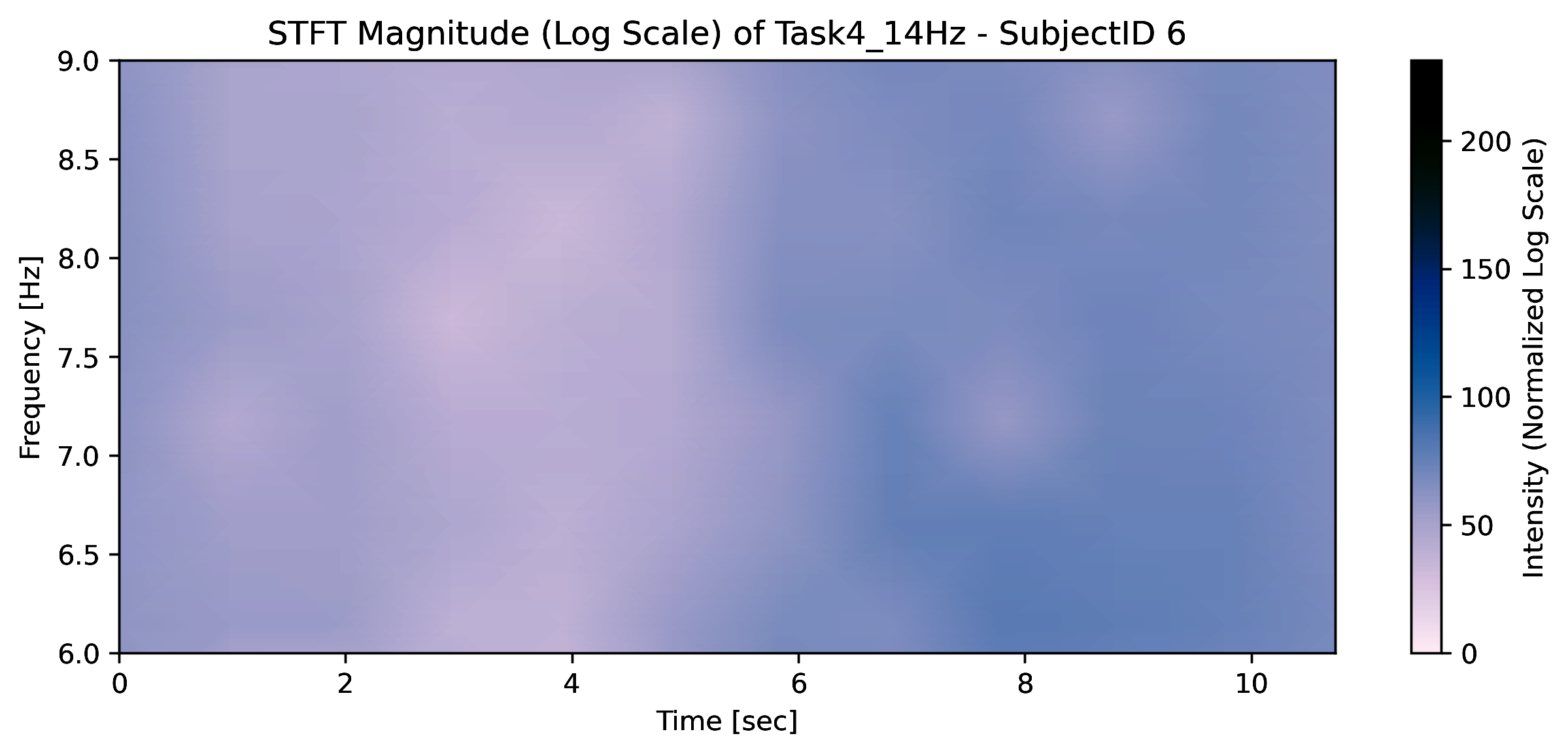

Supplement: Supplementary file 1 [file sensors-26-00157-s001.zip › STFT Images/AFG Images/Task 1-7 Images/S5 Task 4 ID_6.png]

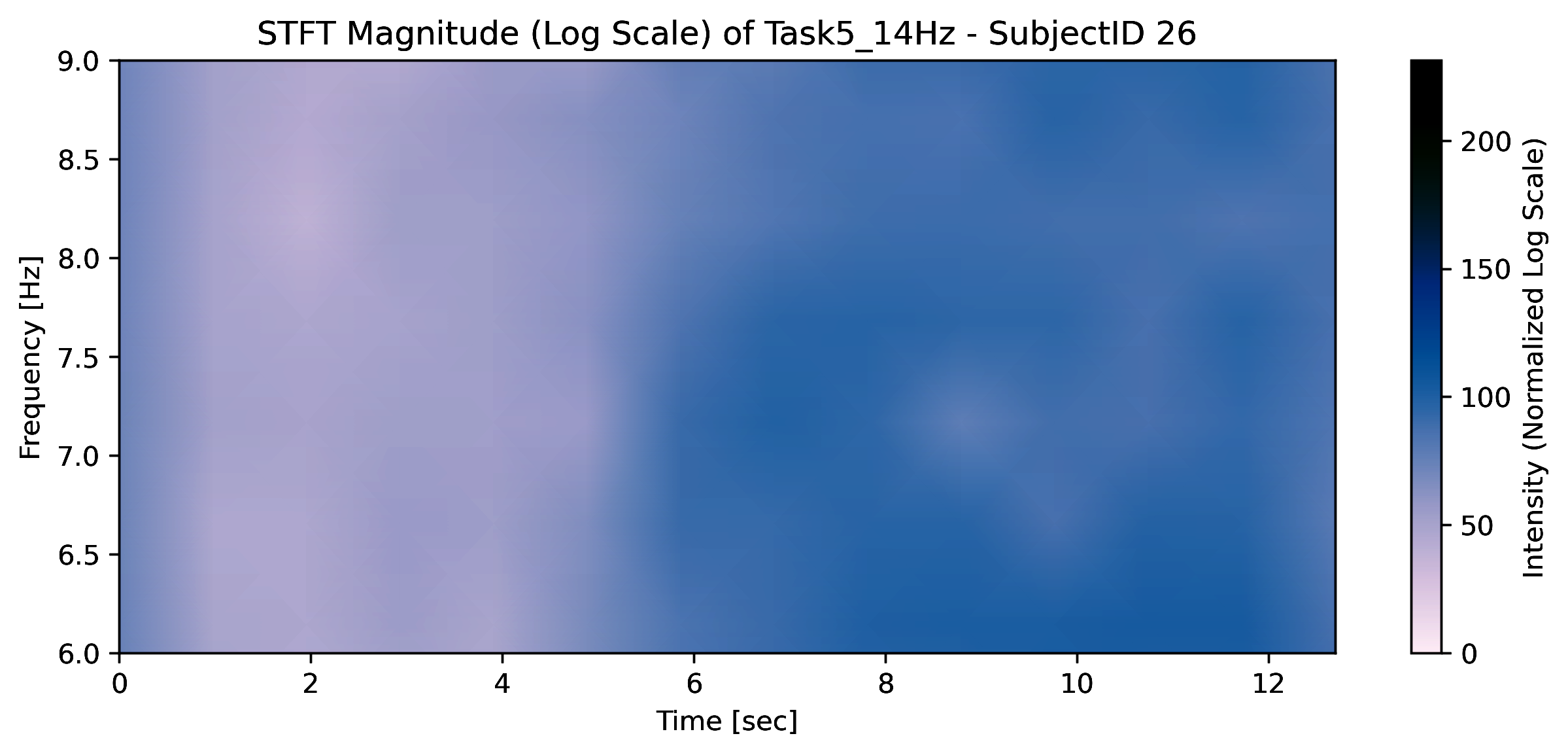

Supplement: Supplementary file 1 [file sensors-26-00157-s001.zip › STFT Images/AFG Images/Task 1-7 Images/S5 Task 5 ID_26.png]

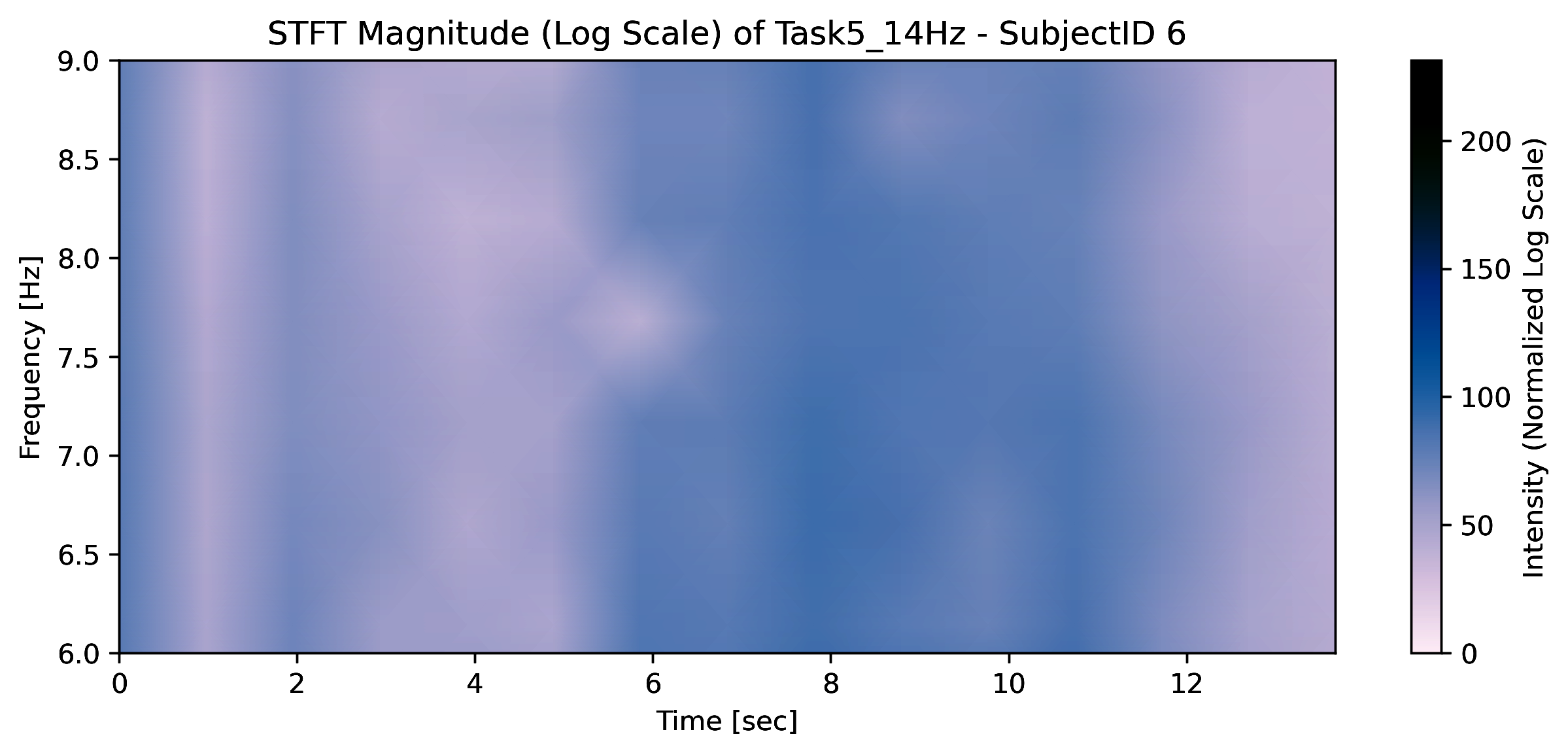

Supplement: Supplementary file 1 [file sensors-26-00157-s001.zip › STFT Images/AFG Images/Task 1-7 Images/S5 Task 5 ID_6.png]

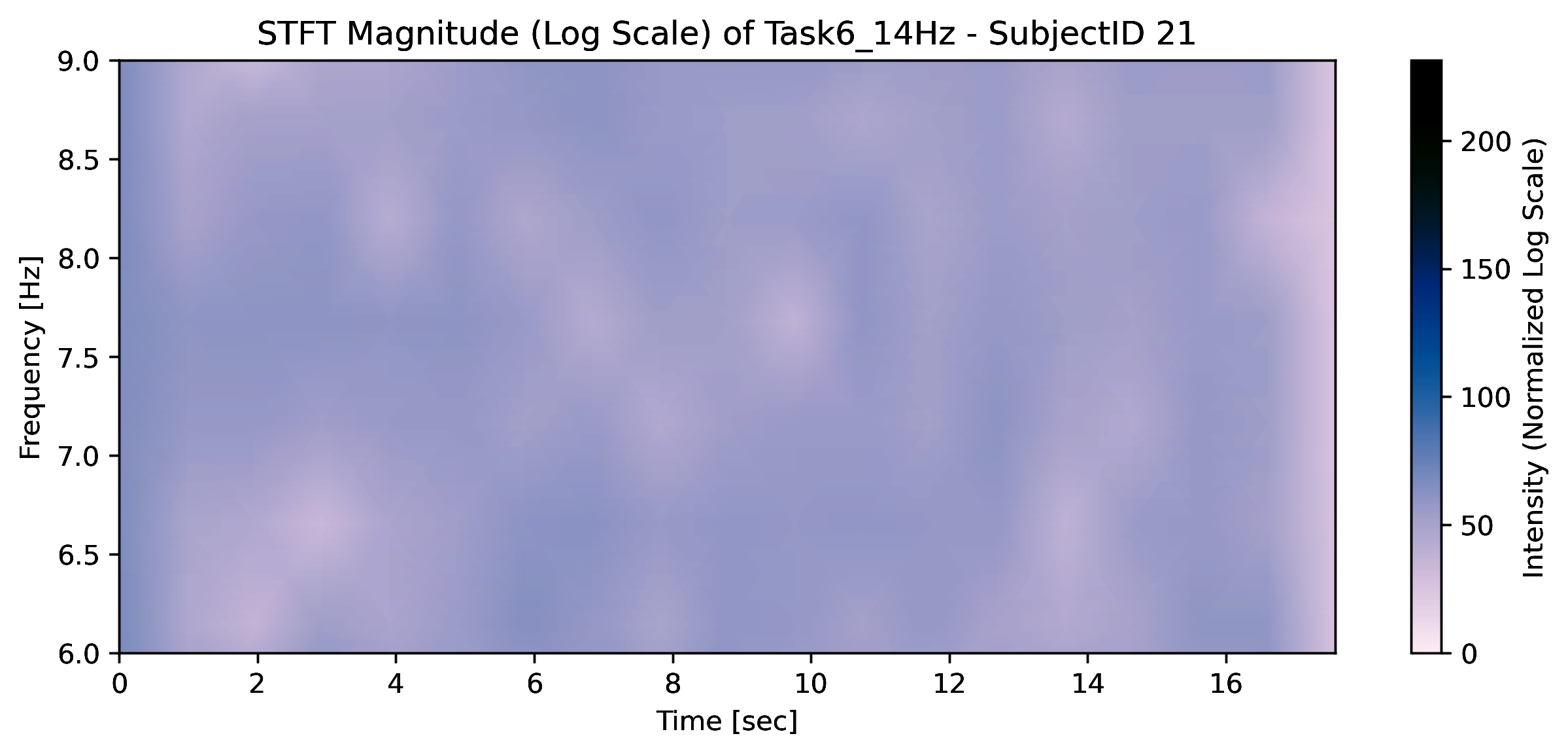

Supplement: Supplementary file 1 [file sensors-26-00157-s001.zip › STFT Images/AFG Images/Task 1-7 Images/S5 Task 6 ID_21.png]

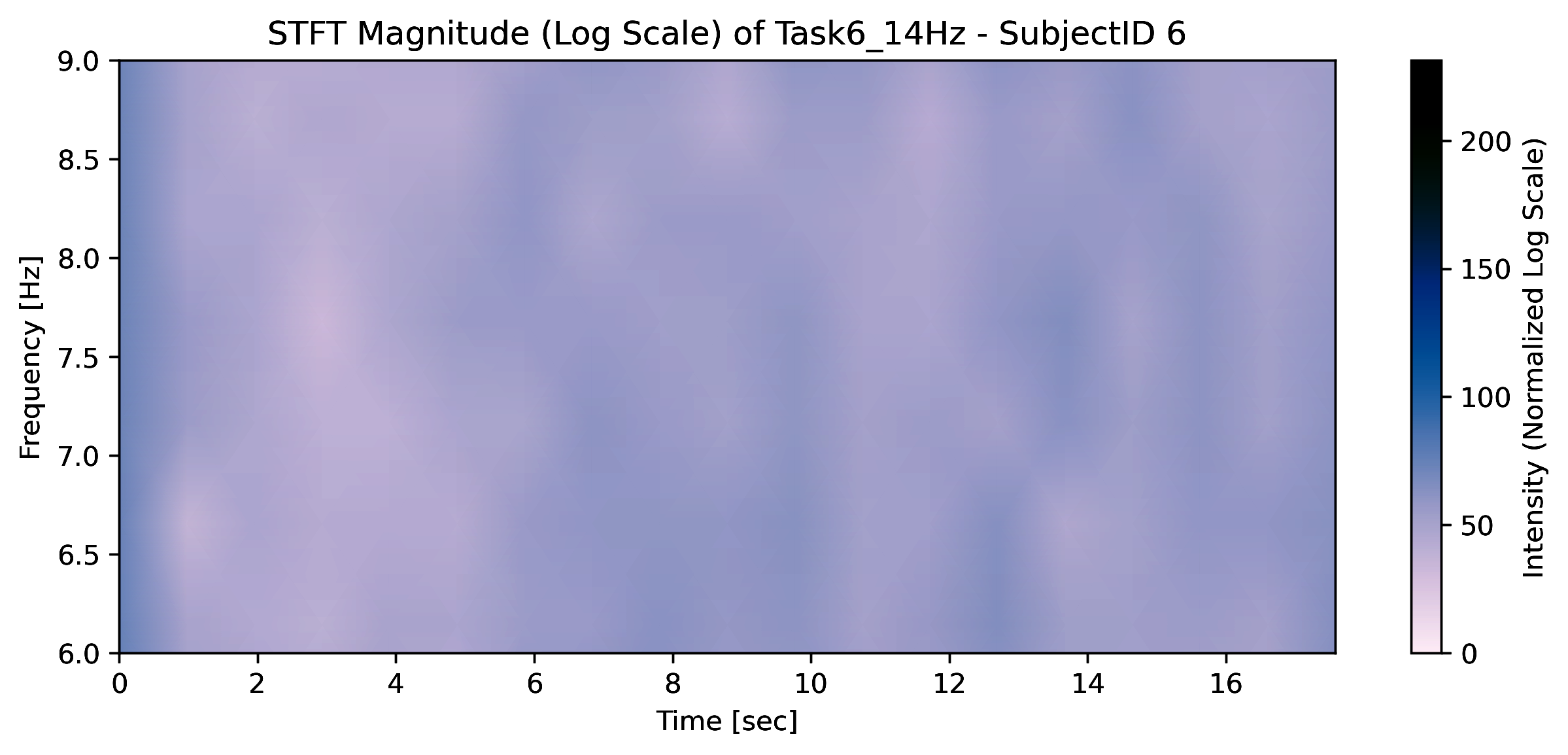

Supplement: Supplementary file 1 [file sensors-26-00157-s001.zip › STFT Images/AFG Images/Task 1-7 Images/S5 Task 6 ID_6.png]

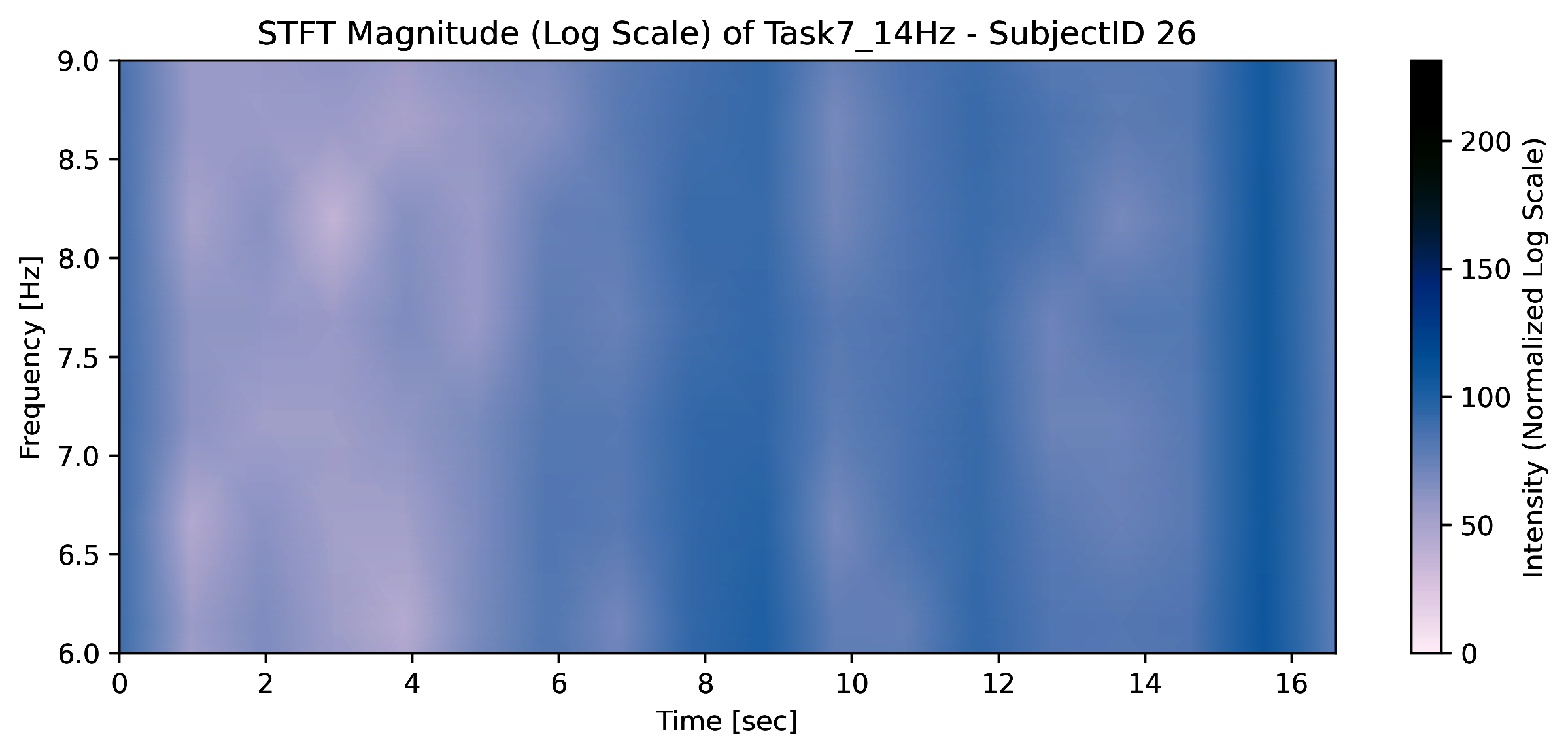

Supplement: Supplementary file 1 [file sensors-26-00157-s001.zip › STFT Images/AFG Images/Task 1-7 Images/S5 Task 7 ID_26.png]

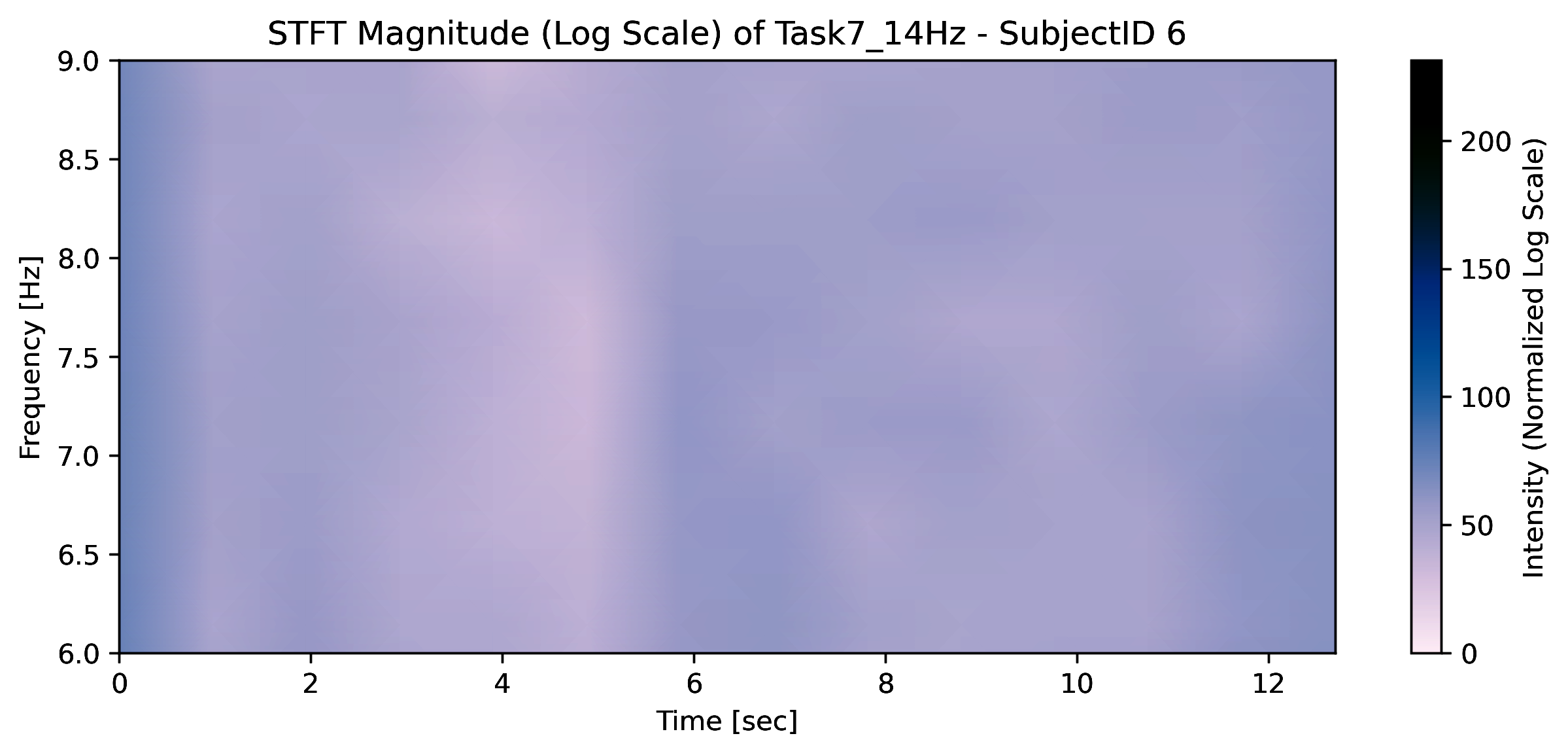

Supplement: Supplementary file 1 [file sensors-26-00157-s001.zip › STFT Images/AFG Images/Task 1-7 Images/S5 Task 7 ID_6.png]

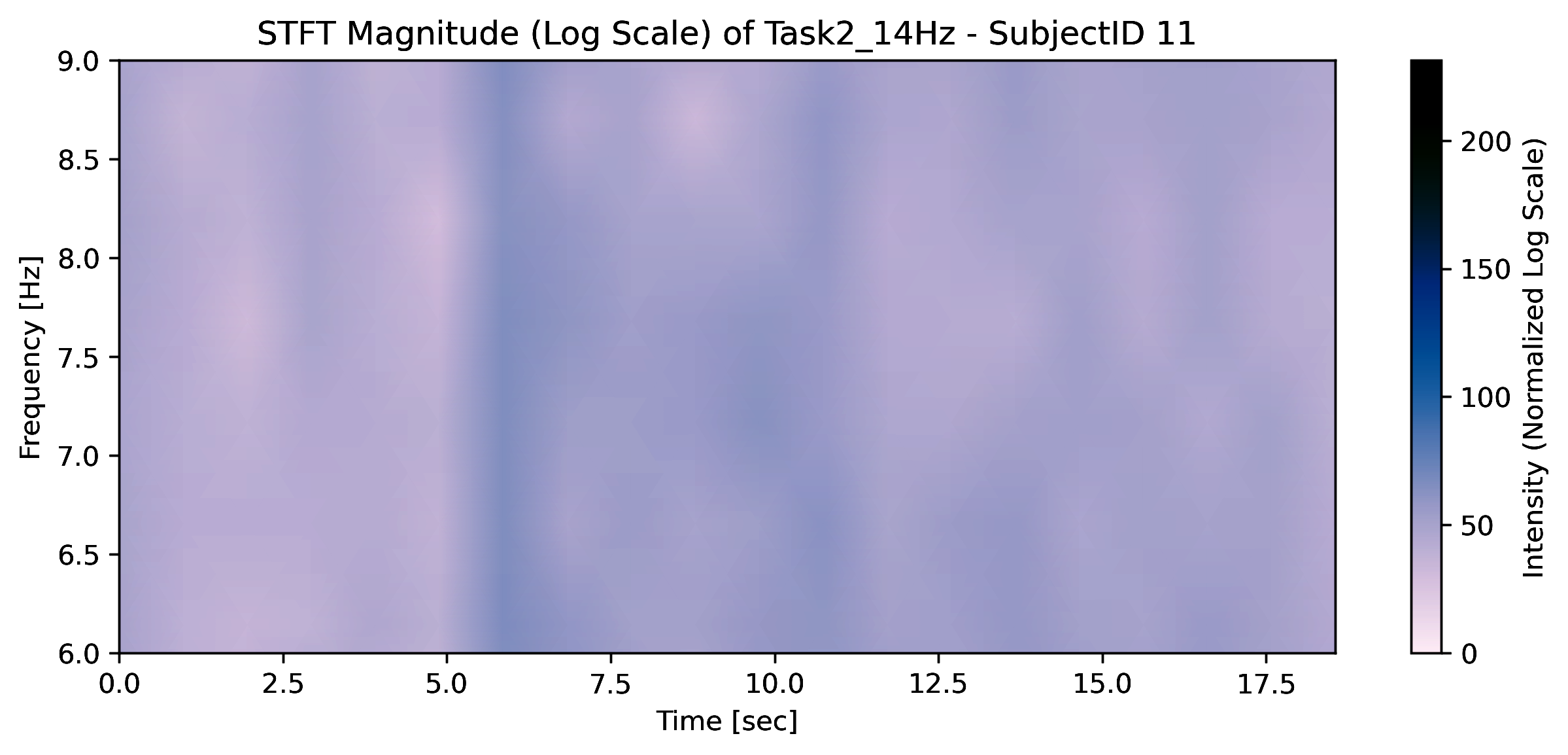

Supplement: Supplementary file 1 [file sensors-26-00157-s001.zip › STFT Images/AFG Images/Task 2 Images/S3 Task 2 ID_11.png]

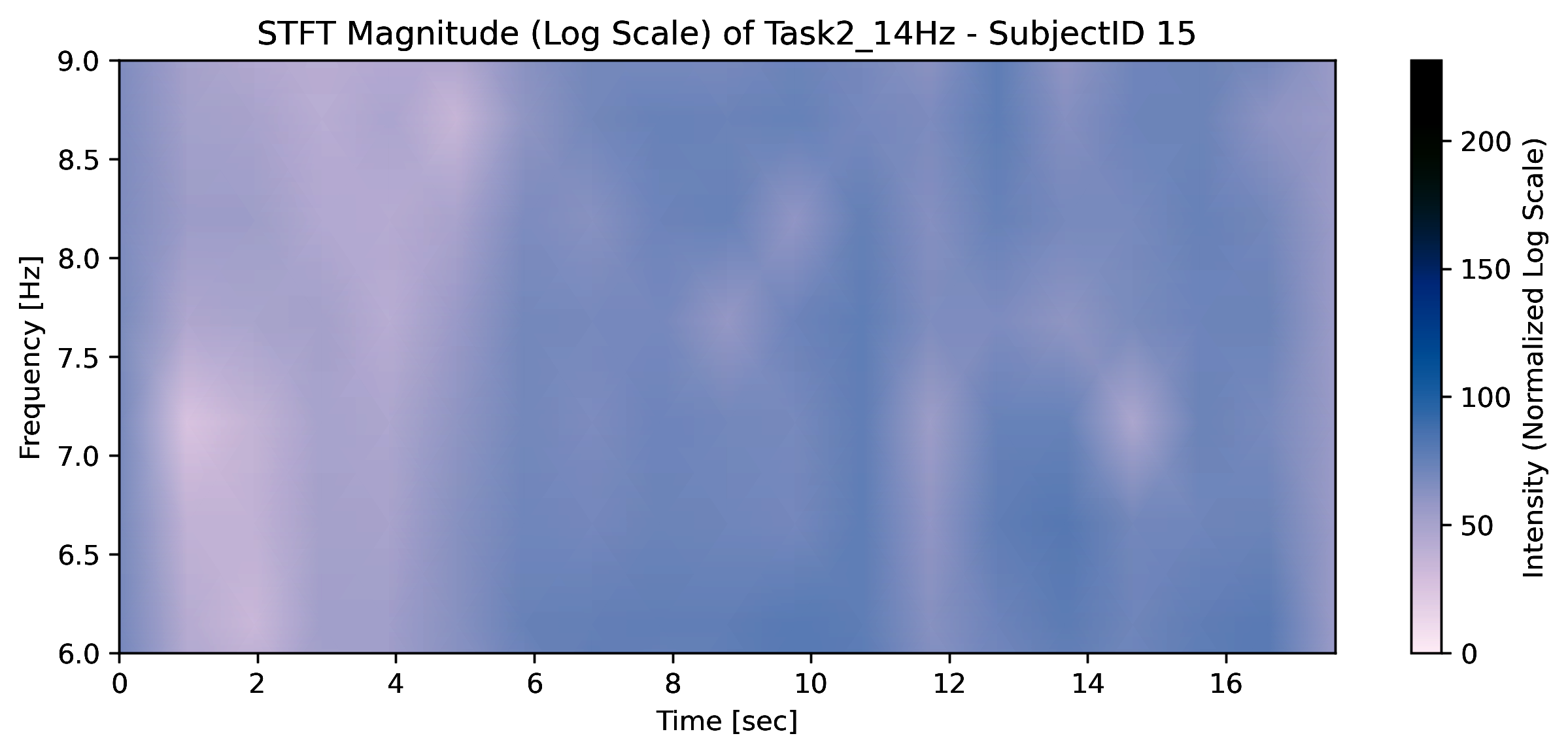

Supplement: Supplementary file 1 [file sensors-26-00157-s001.zip › STFT Images/AFG Images/Task 2 Images/S3 Task 2 ID_15.png]

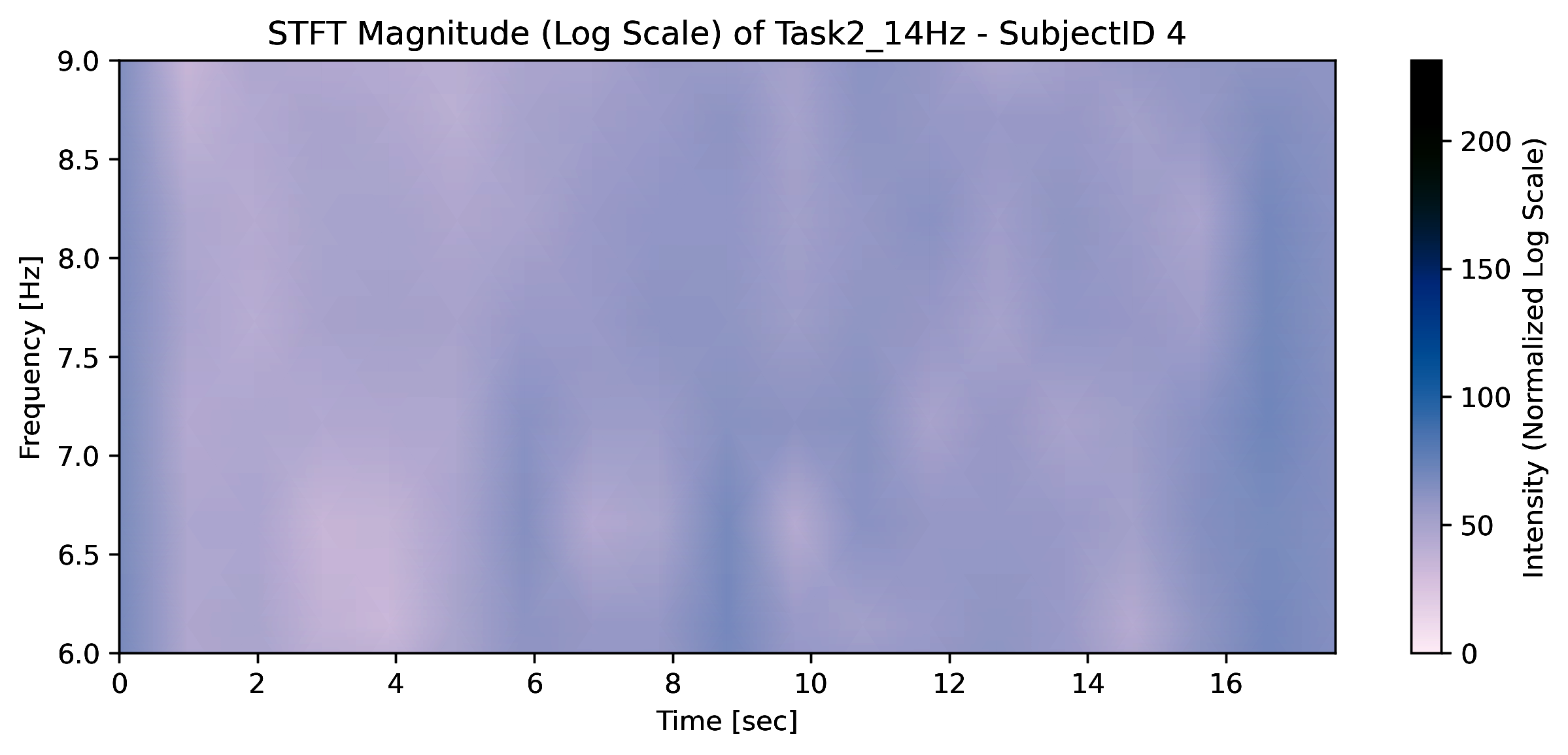

Supplement: Supplementary file 1 [file sensors-26-00157-s001.zip › STFT Images/AFG Images/Task 2 Images/S3 Task 2 ID_4.png]

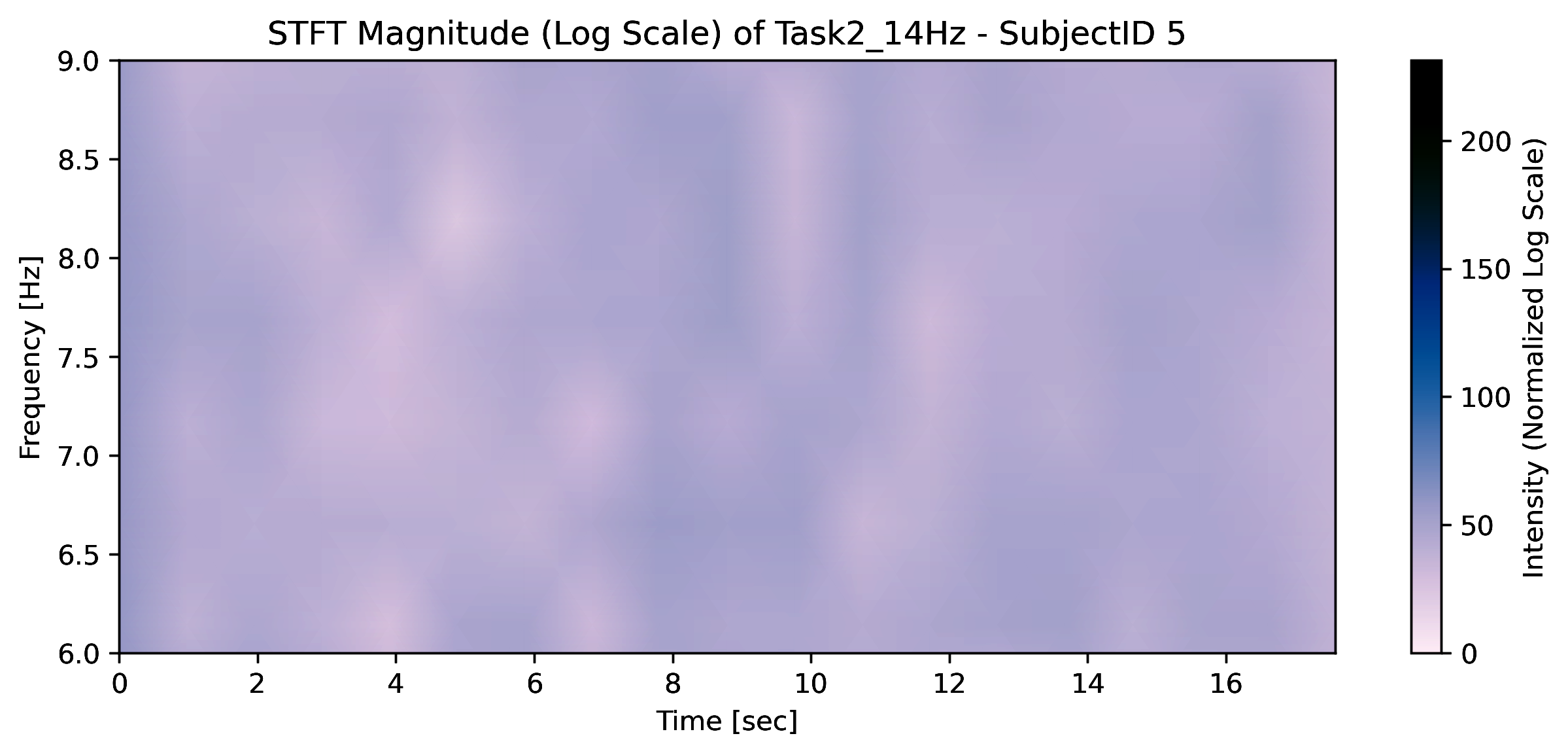

Supplement: Supplementary file 1 [file sensors-26-00157-s001.zip › STFT Images/AFG Images/Task 2 Images/S3 Task 2 ID_5.png]

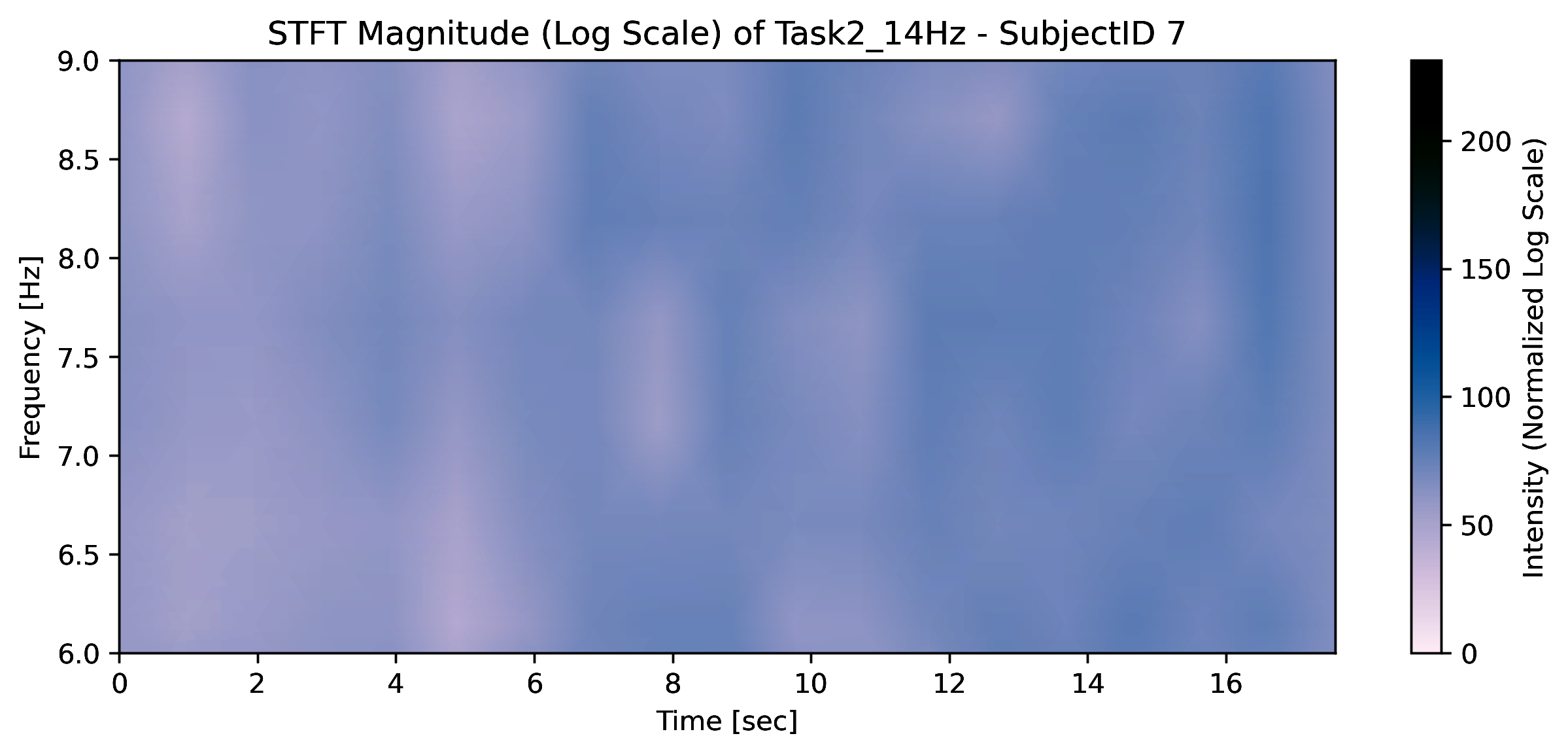

Supplement: Supplementary file 1 [file sensors-26-00157-s001.zip › STFT Images/AFG Images/Task 2 Images/S3 Task 2 ID_7.png]

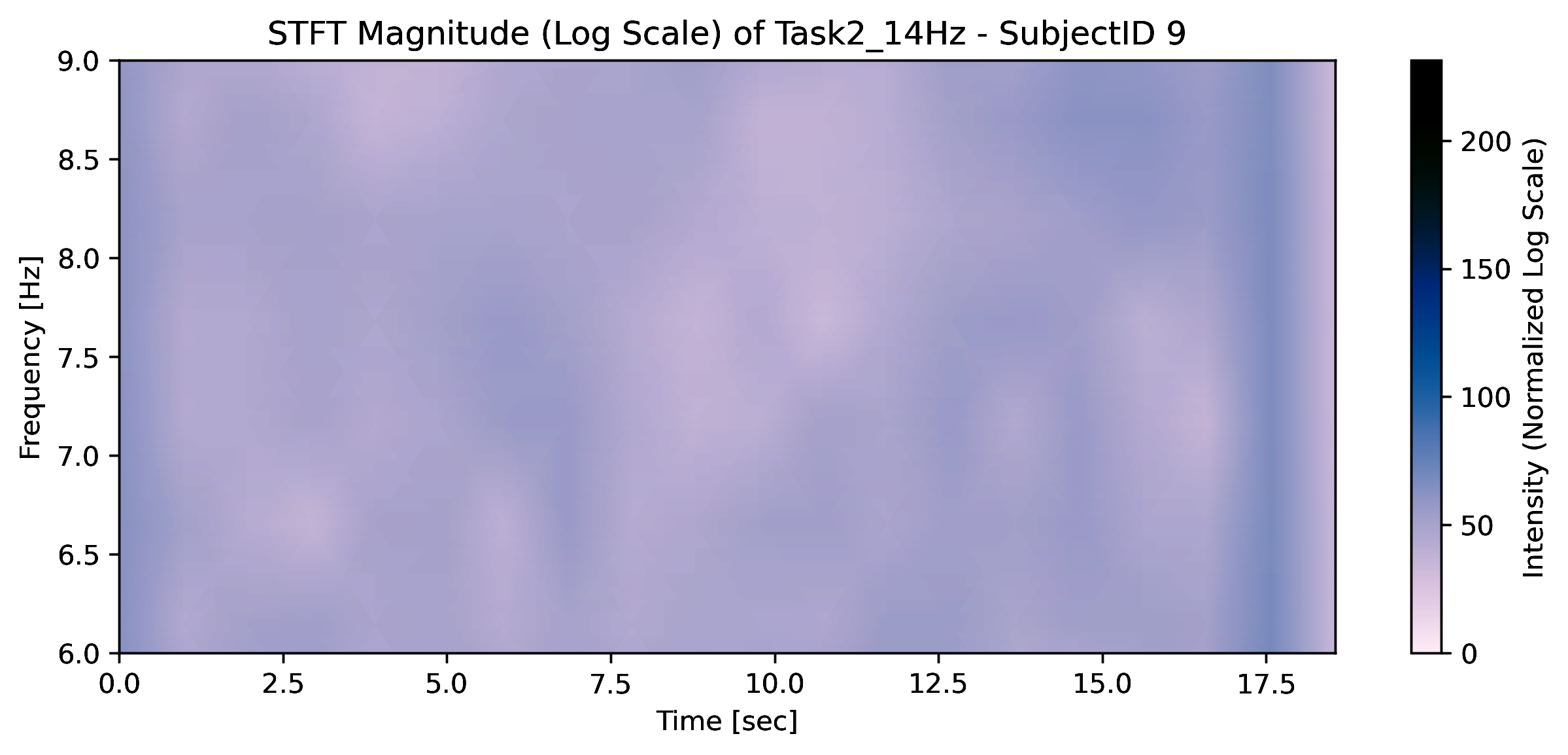

Supplement: Supplementary file 1 [file sensors-26-00157-s001.zip › STFT Images/AFG Images/Task 2 Images/S3 Task 2 ID_9.png]

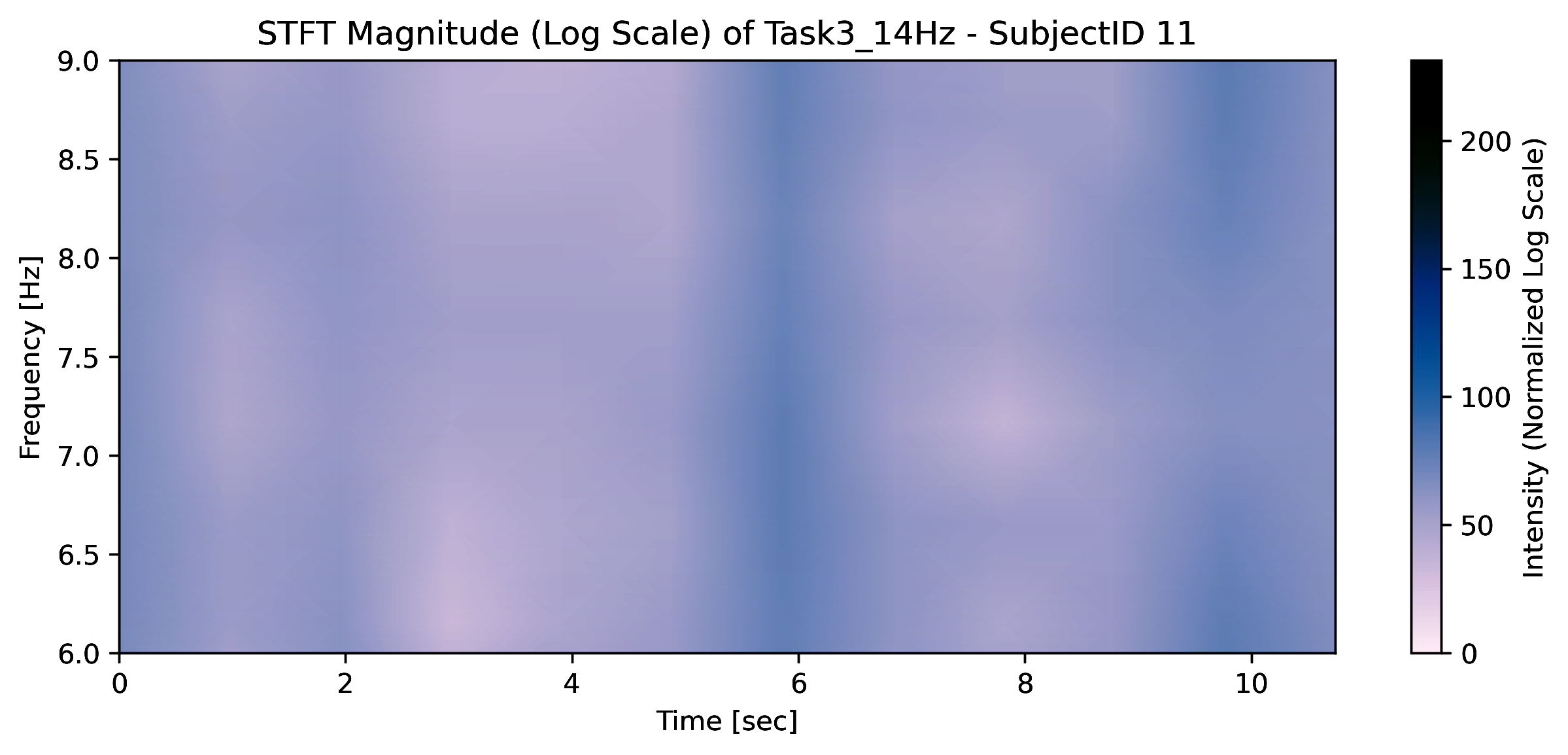

Supplement: Supplementary file 1 [file sensors-26-00157-s001.zip › STFT Images/AFG Images/Task 3 Images/S4 Task 3 ID_11.png]

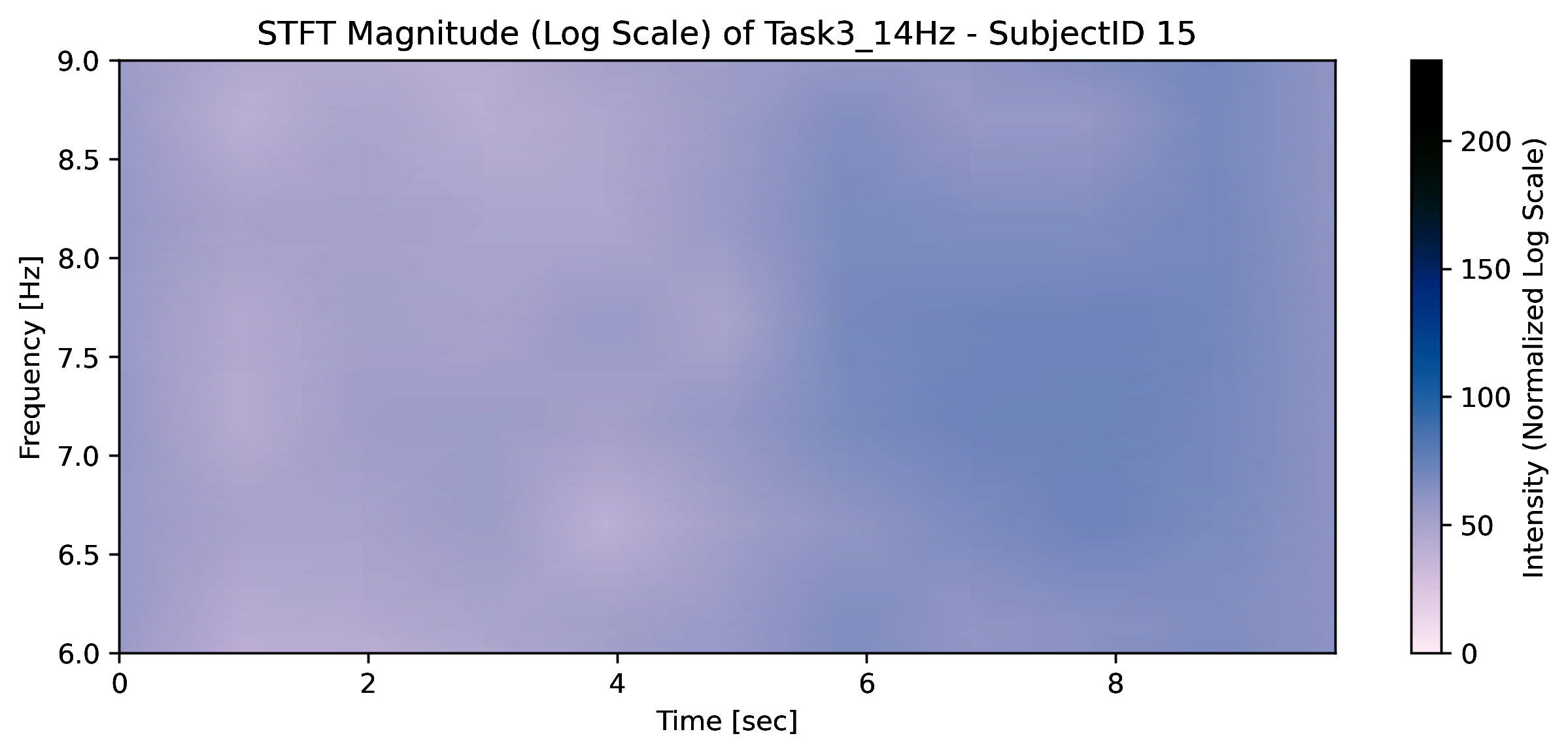

Supplement: Supplementary file 1 [file sensors-26-00157-s001.zip › STFT Images/AFG Images/Task 3 Images/S4 Task 3 ID_15.png]

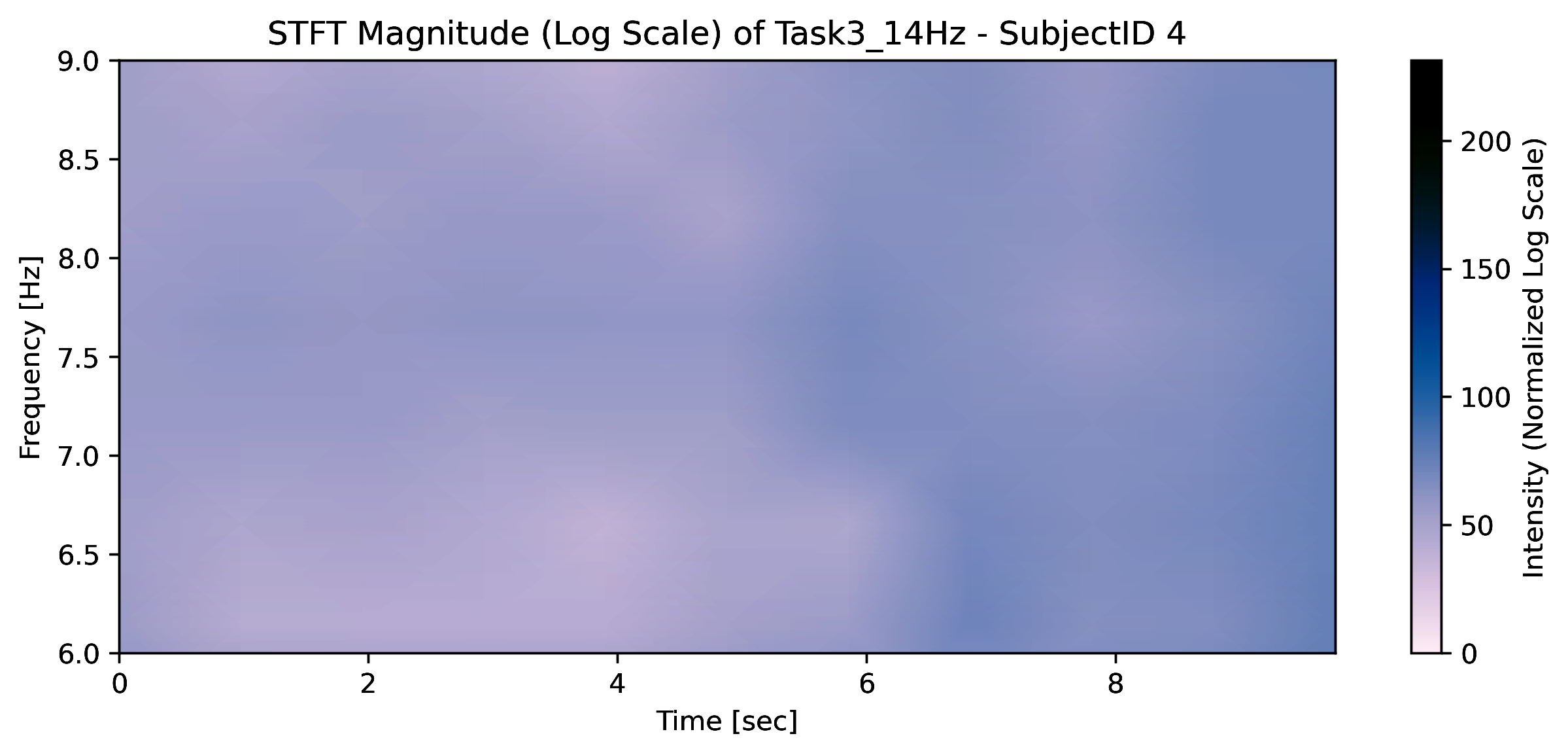

Supplement: Supplementary file 1 [file sensors-26-00157-s001.zip › STFT Images/AFG Images/Task 3 Images/S4 Task 3 ID_4.png]

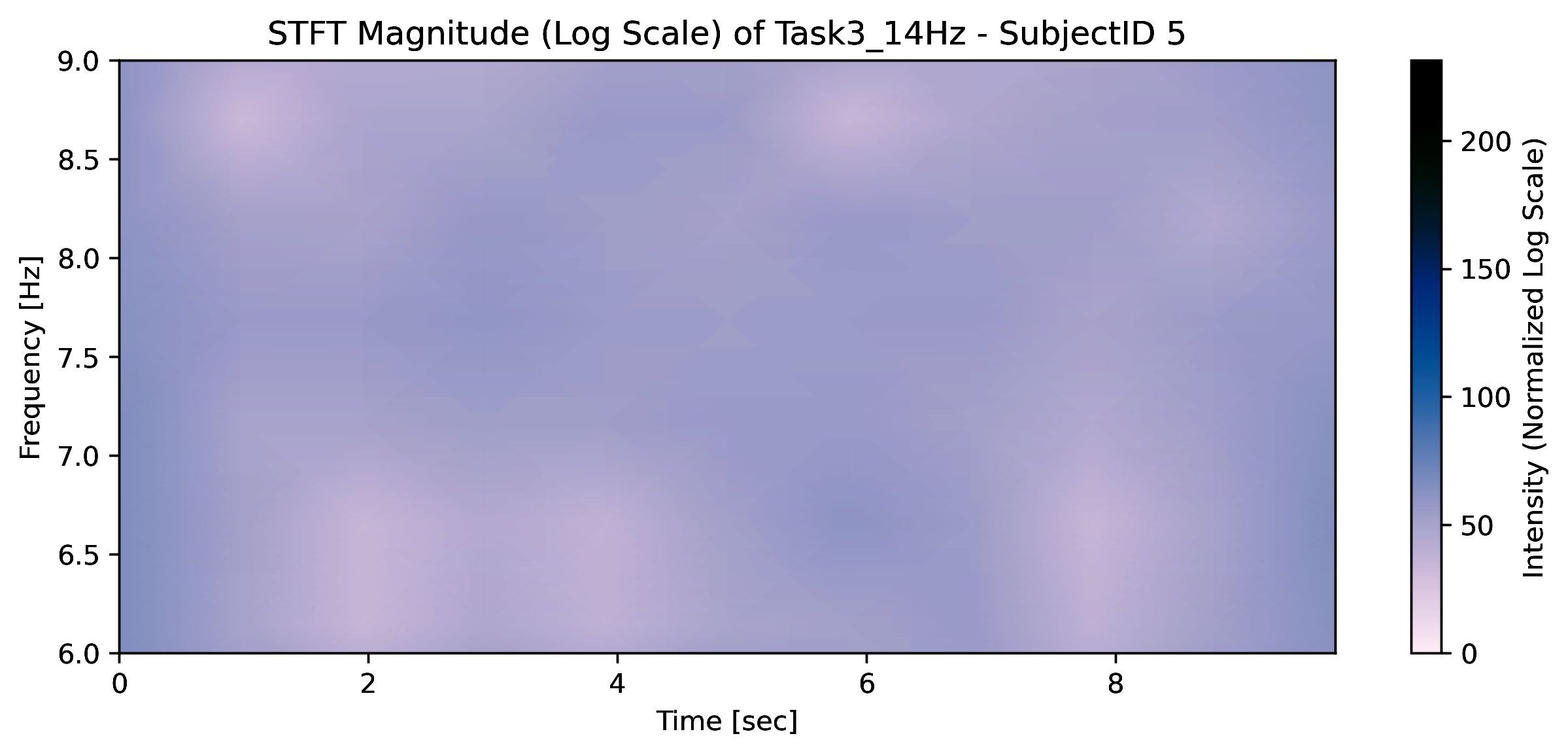

Supplement: Supplementary file 1 [file sensors-26-00157-s001.zip › STFT Images/AFG Images/Task 3 Images/S4 Task 3 ID_5.png]

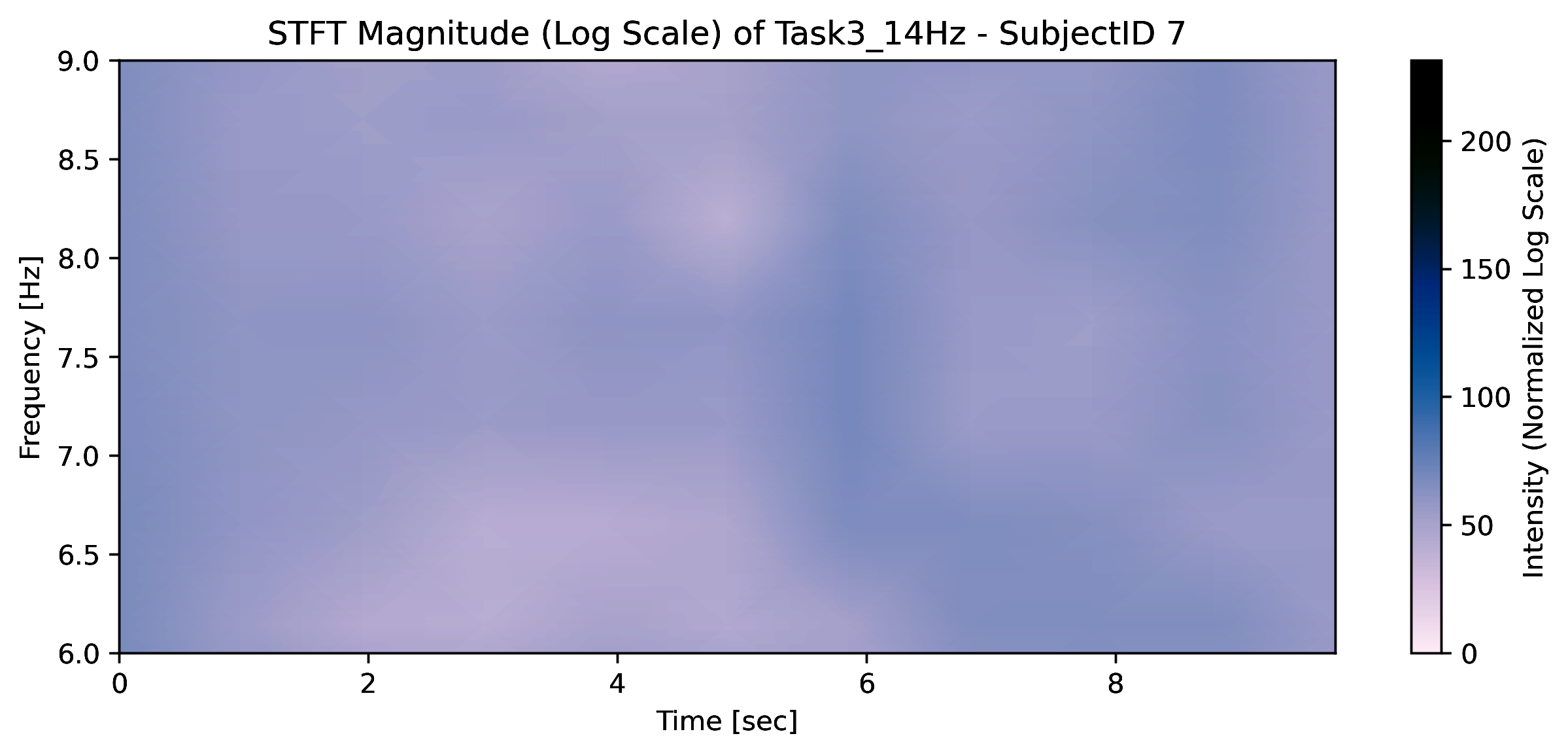

Supplement: Supplementary file 1 [file sensors-26-00157-s001.zip › STFT Images/AFG Images/Task 3 Images/S4 Task 3 ID_7.png]

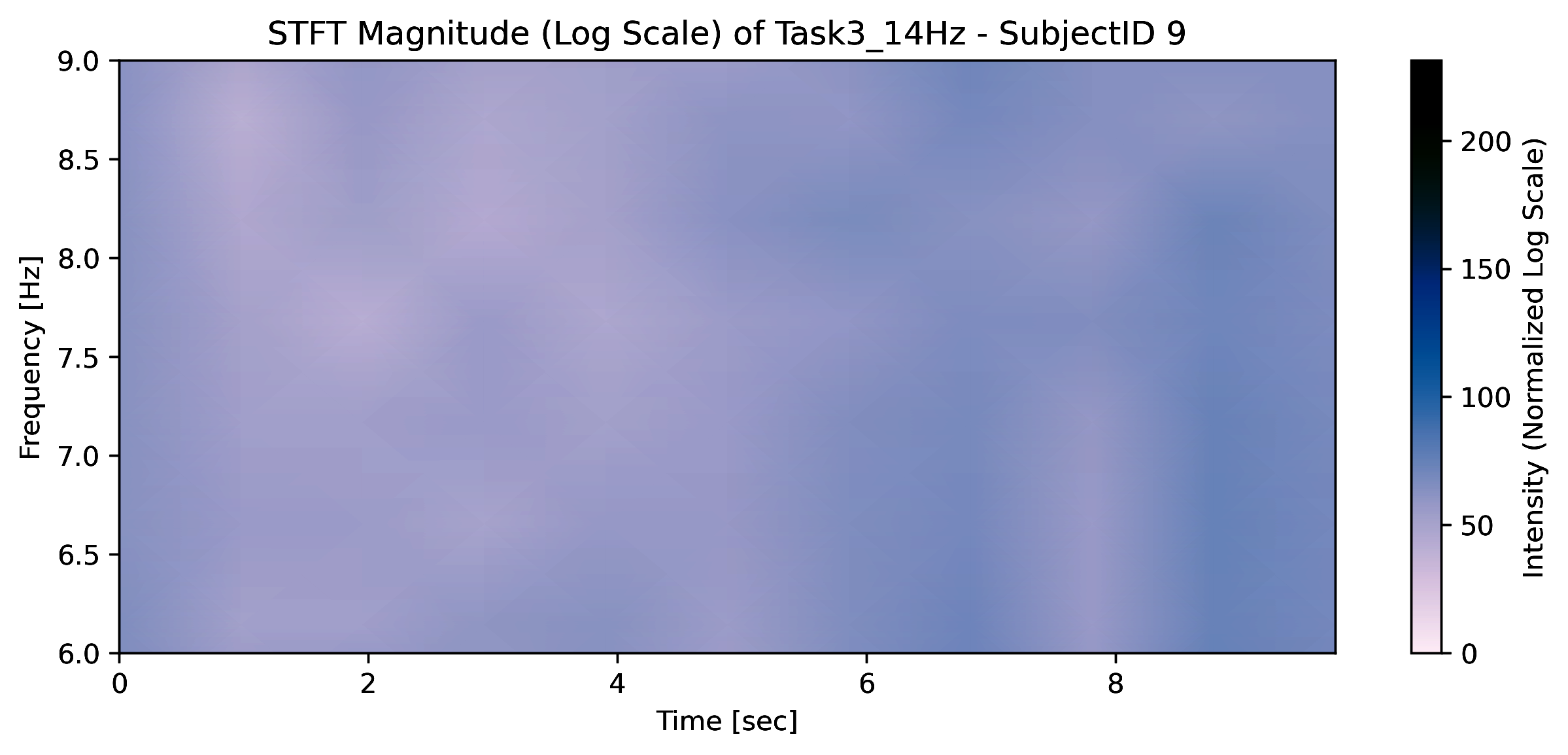

Supplement: Supplementary file 1 [file sensors-26-00157-s001.zip › STFT Images/AFG Images/Task 3 Images/S4 Task 3 ID_9.png]

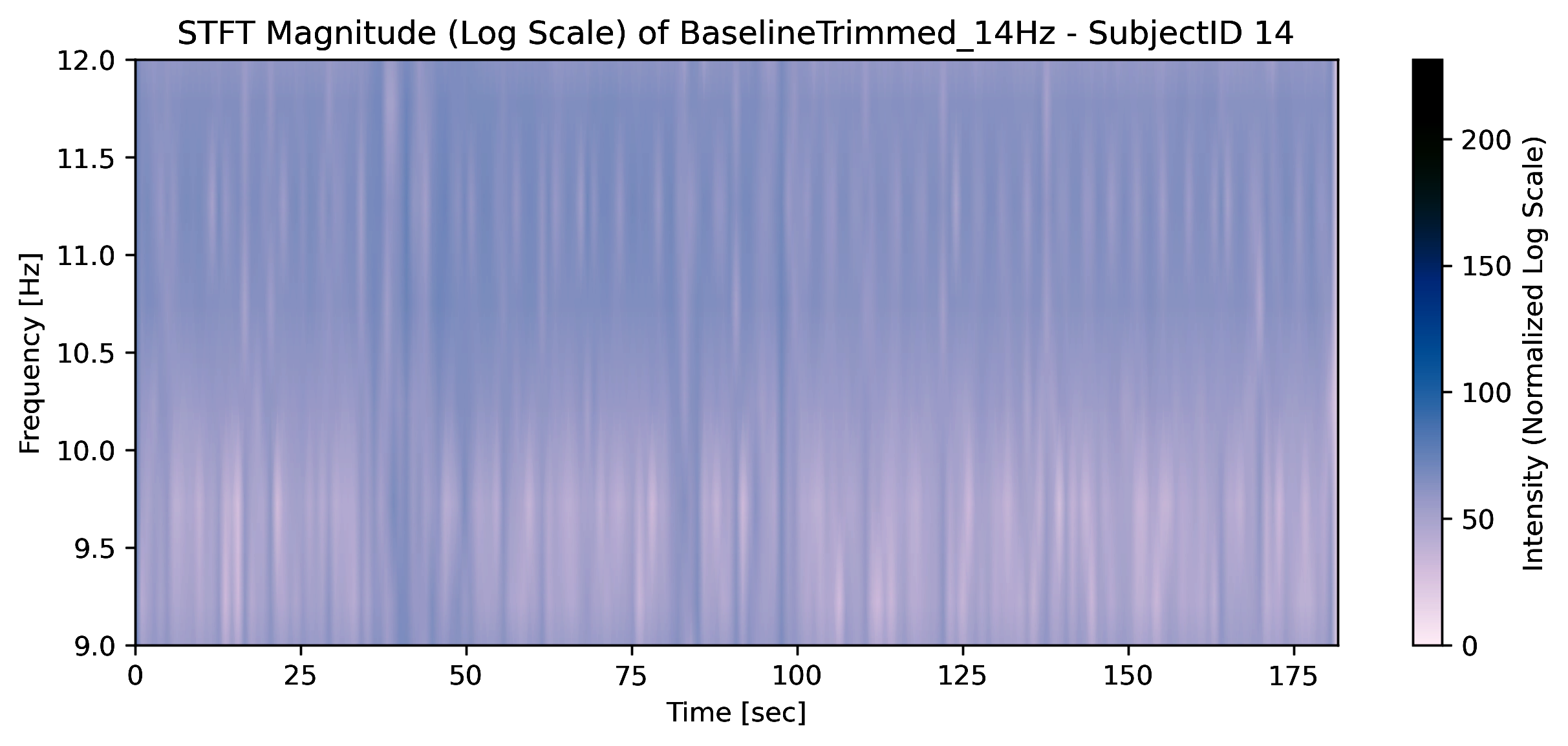

Supplement: Supplementary file 1 [file sensors-26-00157-s001.zip › STFT Images/PFG Images/Baseline Images/S6A ID_14.png]

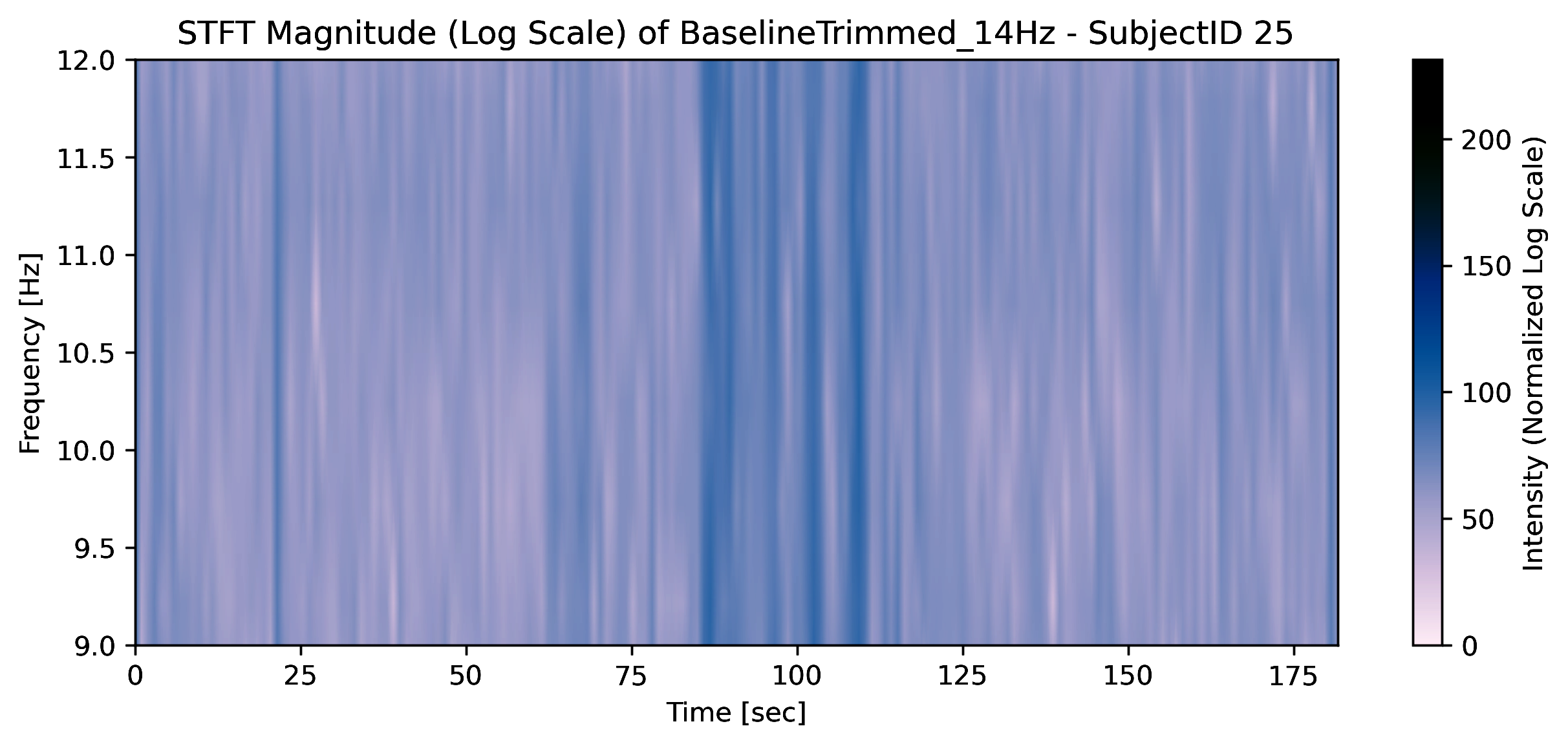

Supplement: Supplementary file 1 [file sensors-26-00157-s001.zip › STFT Images/PFG Images/Baseline Images/S6B ID_25.png]

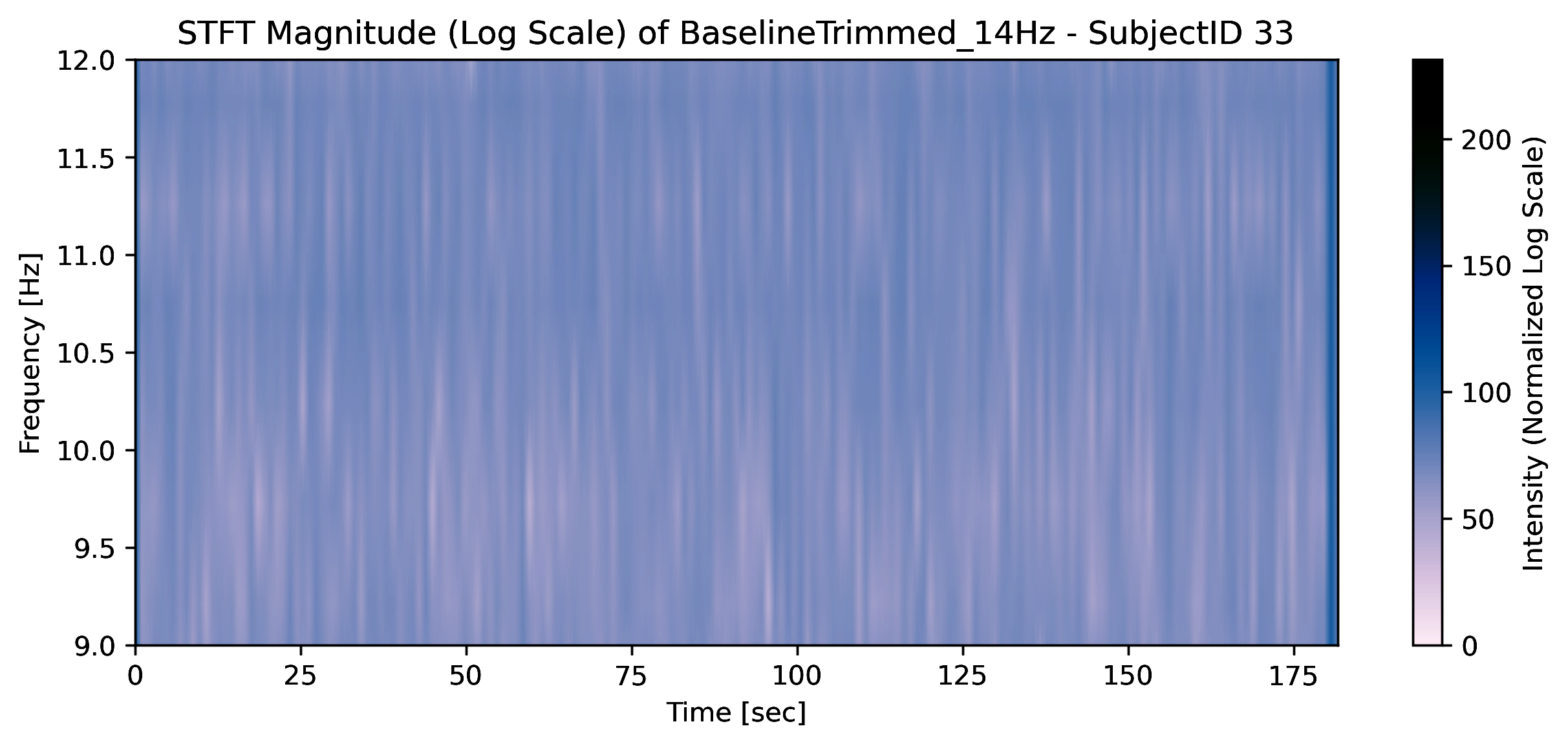

Supplement: Supplementary file 1 [file sensors-26-00157-s001.zip › STFT Images/PFG Images/Baseline Images/S6C ID_33.png]

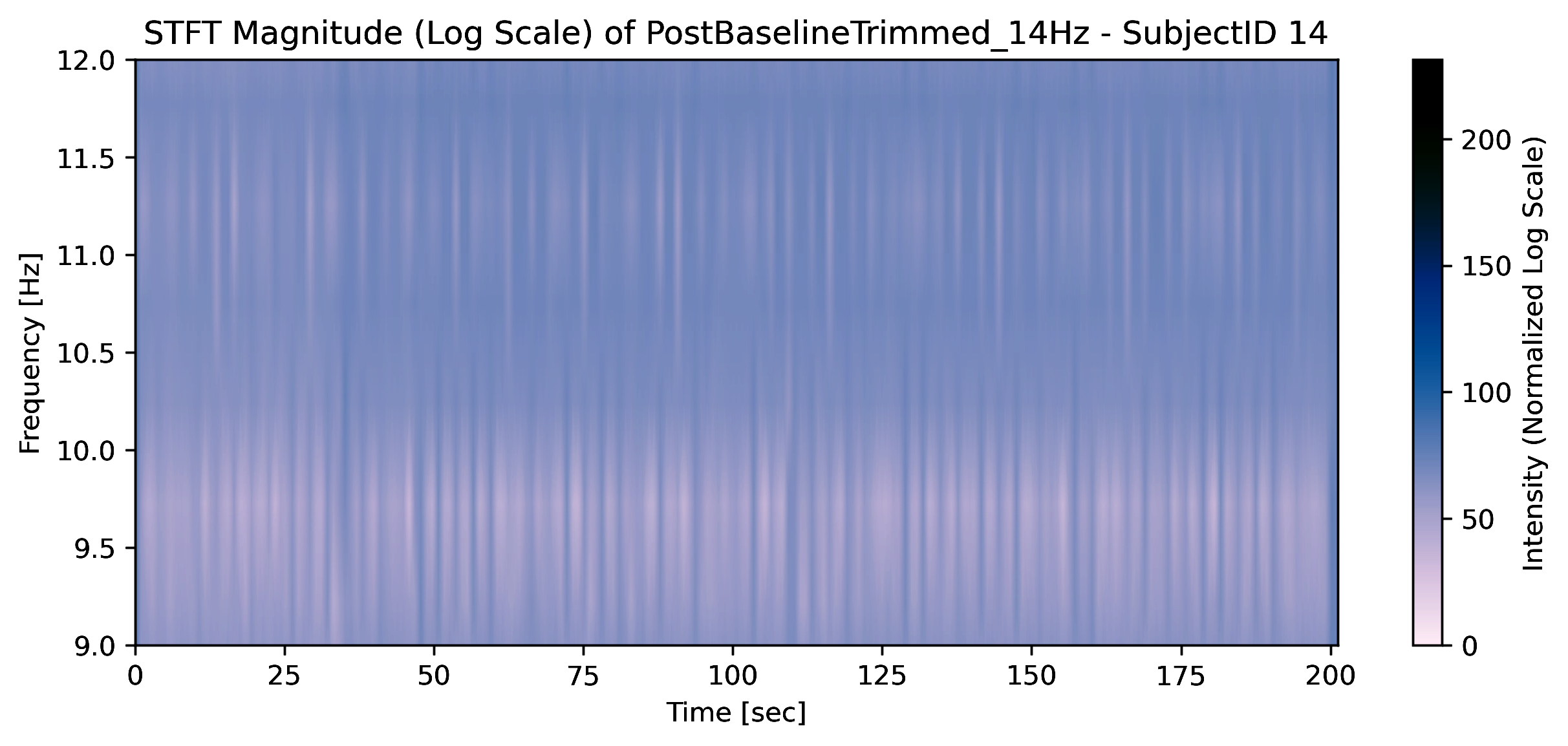

Supplement: Supplementary file 1 [file sensors-26-00157-s001.zip › STFT Images/PFG Images/PostBaseline Images/S7A ID_14.png]

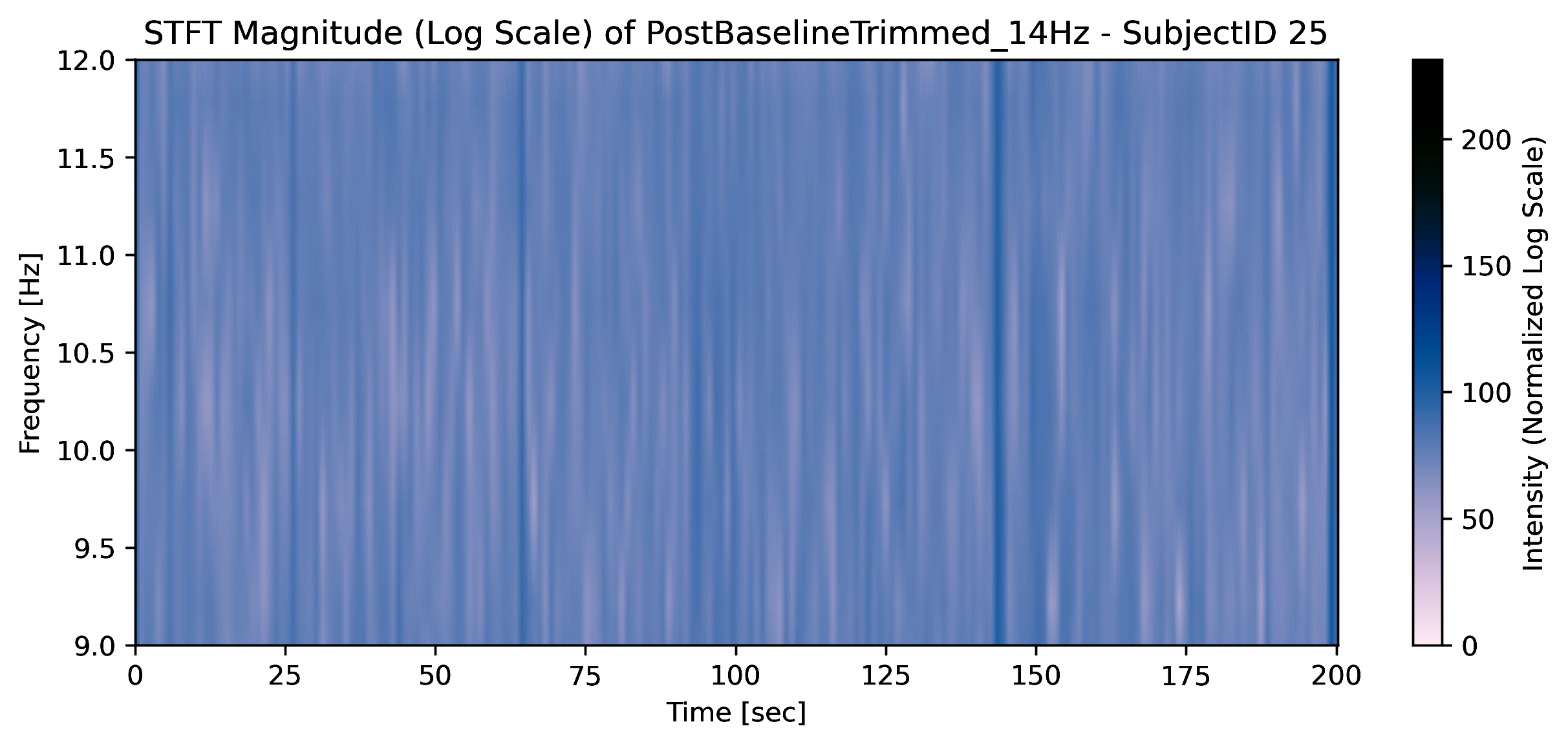

Supplement: Supplementary file 1 [file sensors-26-00157-s001.zip › STFT Images/PFG Images/PostBaseline Images/S7B ID_25.png]

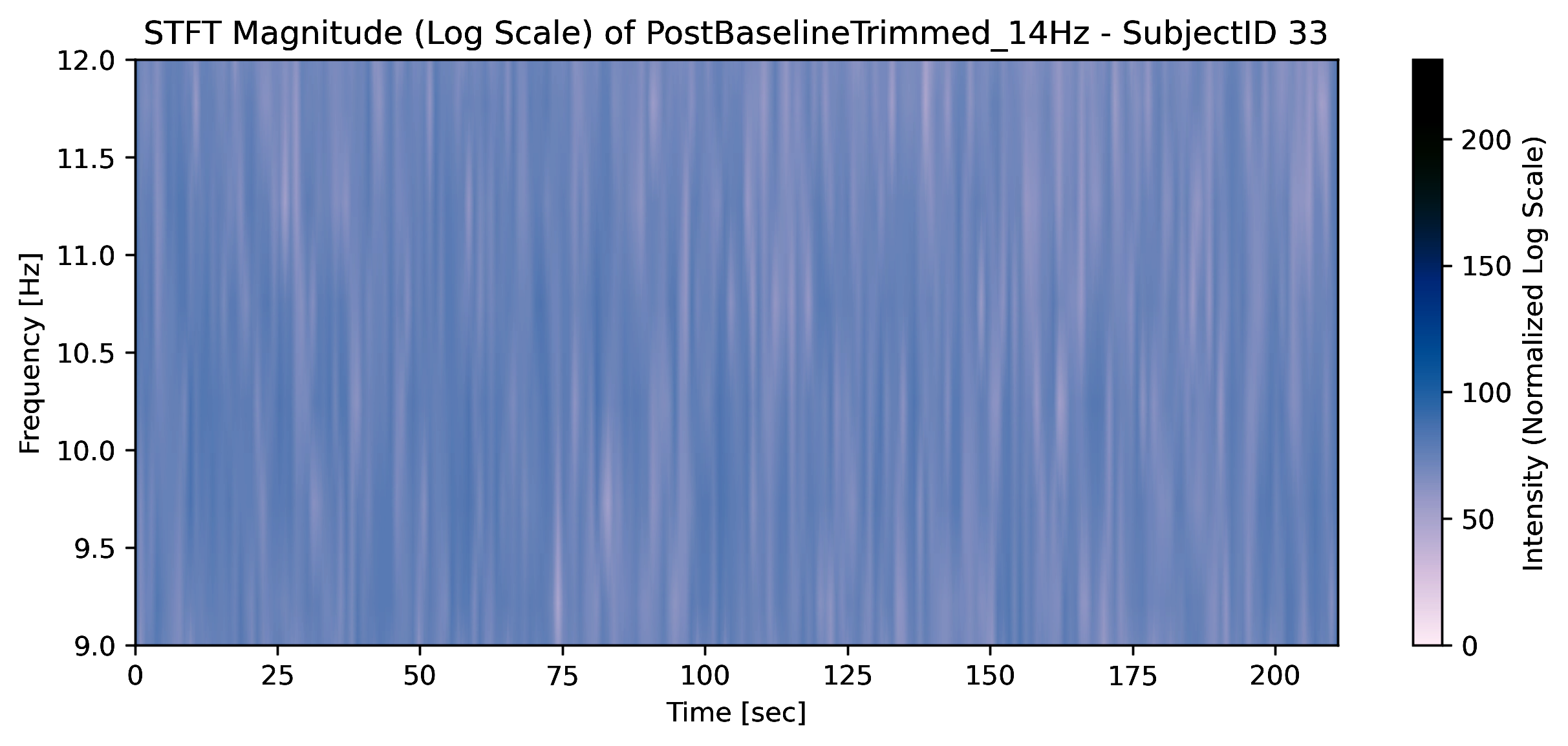

Supplement: Supplementary file 1 [file sensors-26-00157-s001.zip › STFT Images/PFG Images/PostBaseline Images/S7C ID_33.png]

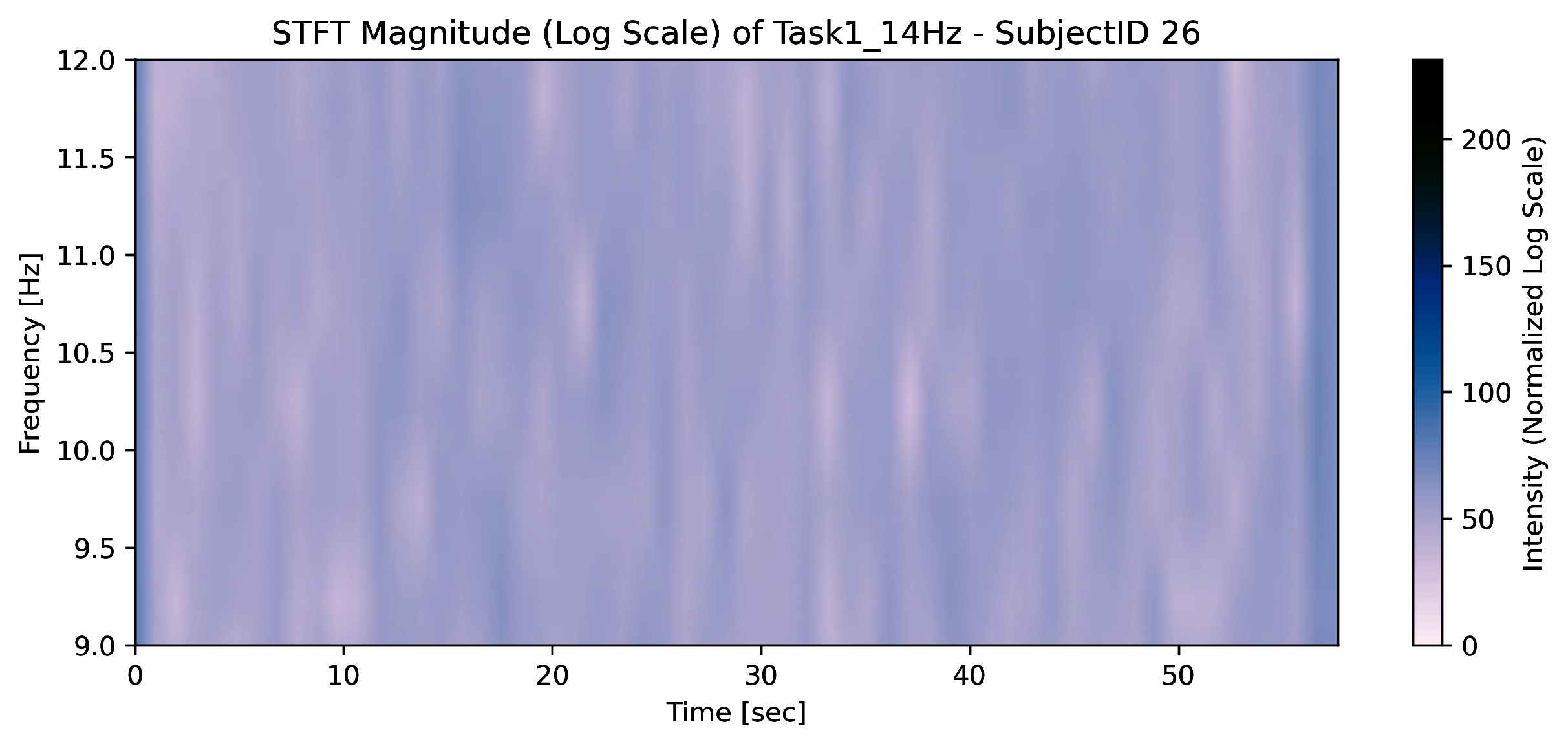

Supplement: Supplementary file 1 [file sensors-26-00157-s001.zip › STFT Images/PFG Images/Task 1-7 Images/S10 Task 1 ID_26.png]

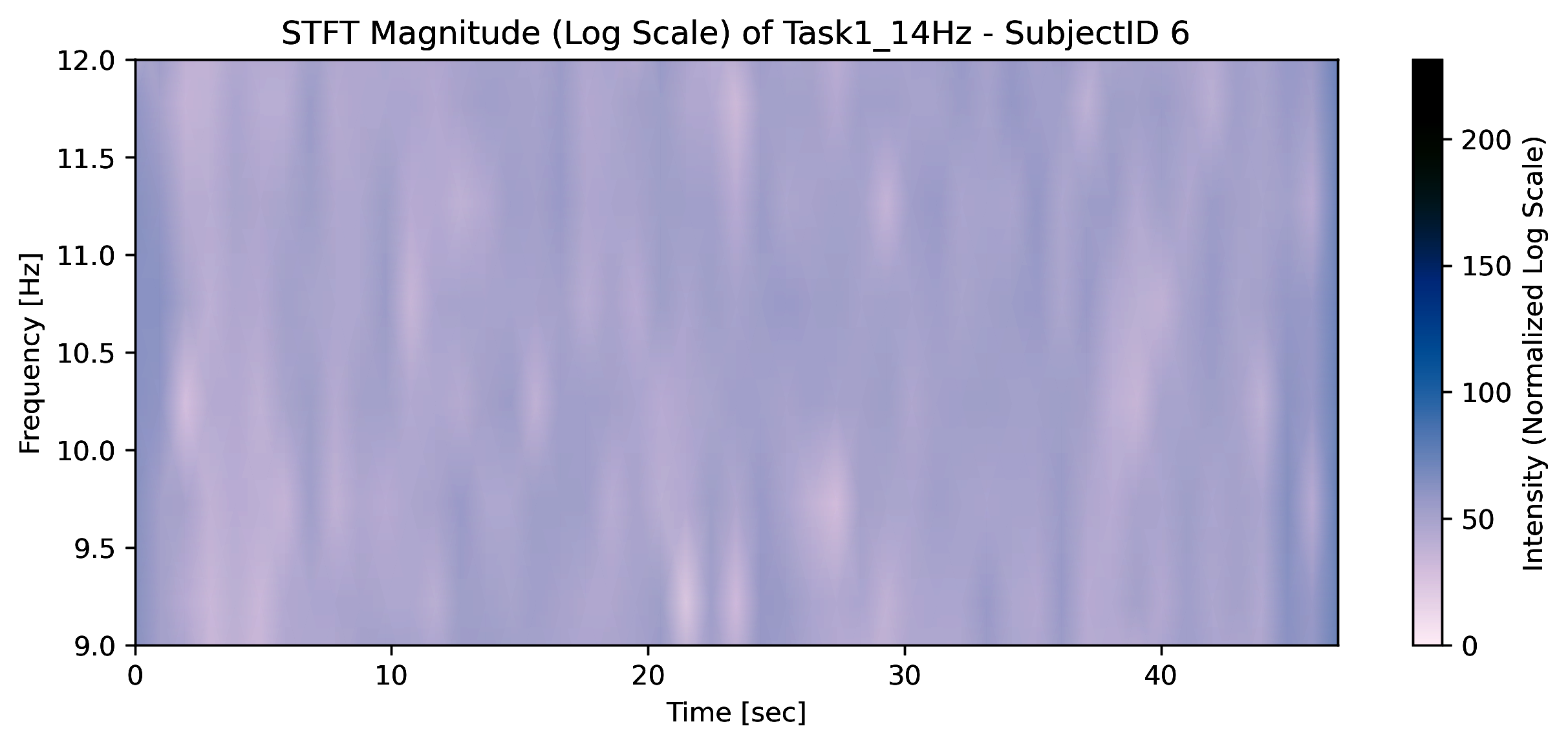

Supplement: Supplementary file 1 [file sensors-26-00157-s001.zip › STFT Images/PFG Images/Task 1-7 Images/S10 Task 1 ID_6.png]

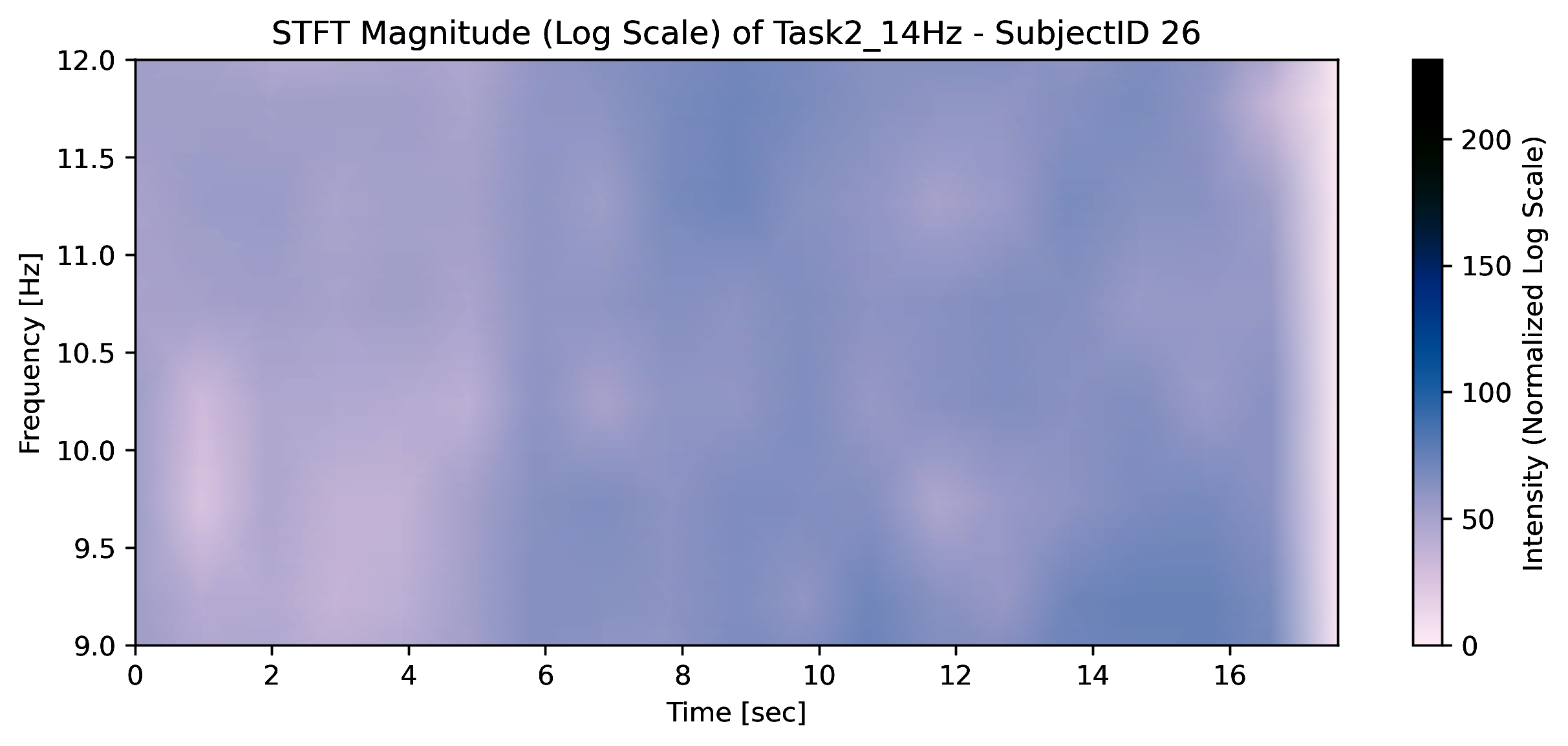

Supplement: Supplementary file 1 [file sensors-26-00157-s001.zip › STFT Images/PFG Images/Task 1-7 Images/S10 Task 2 ID_26.png]

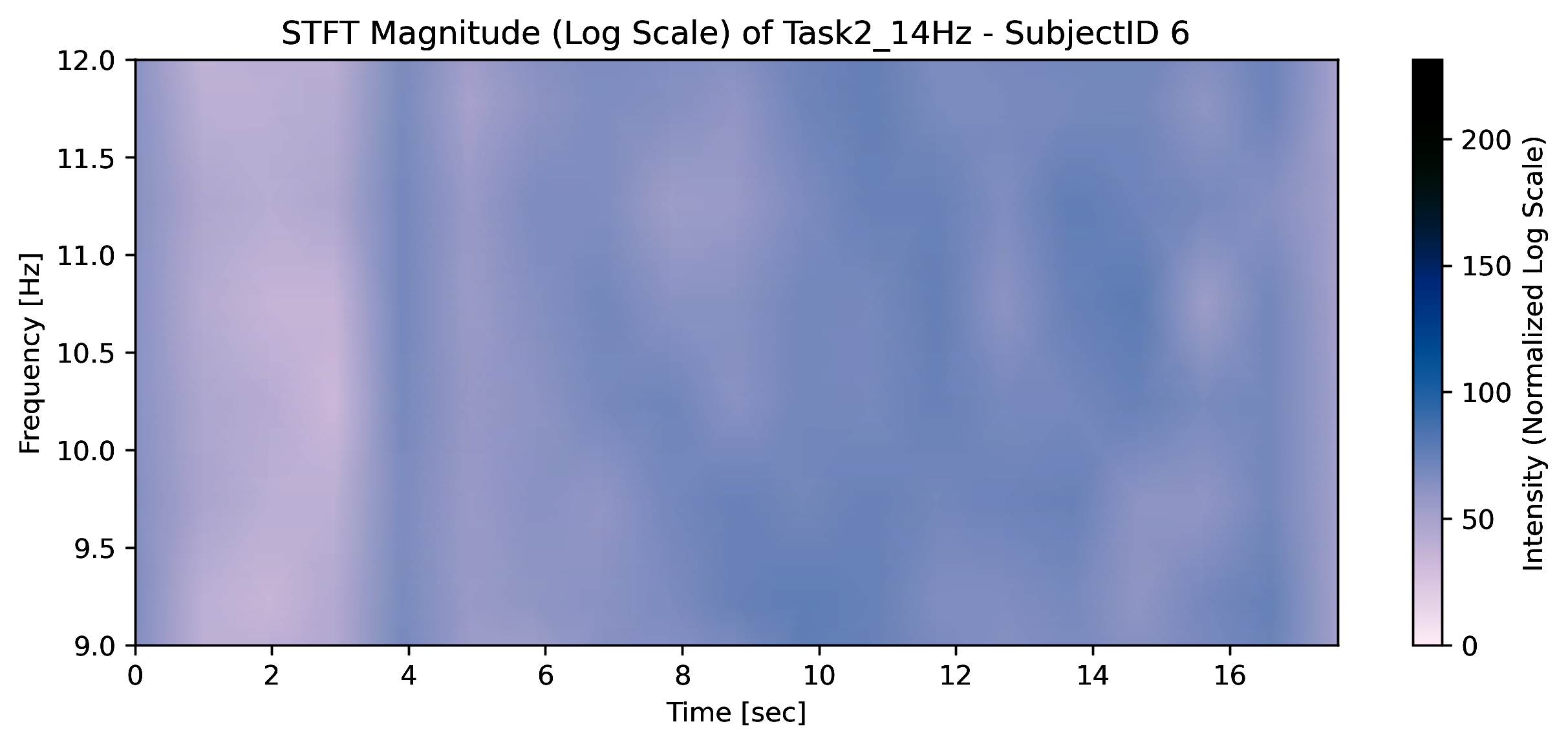

Supplement: Supplementary file 1 [file sensors-26-00157-s001.zip › STFT Images/PFG Images/Task 1-7 Images/S10 Task 2 ID_6.png]

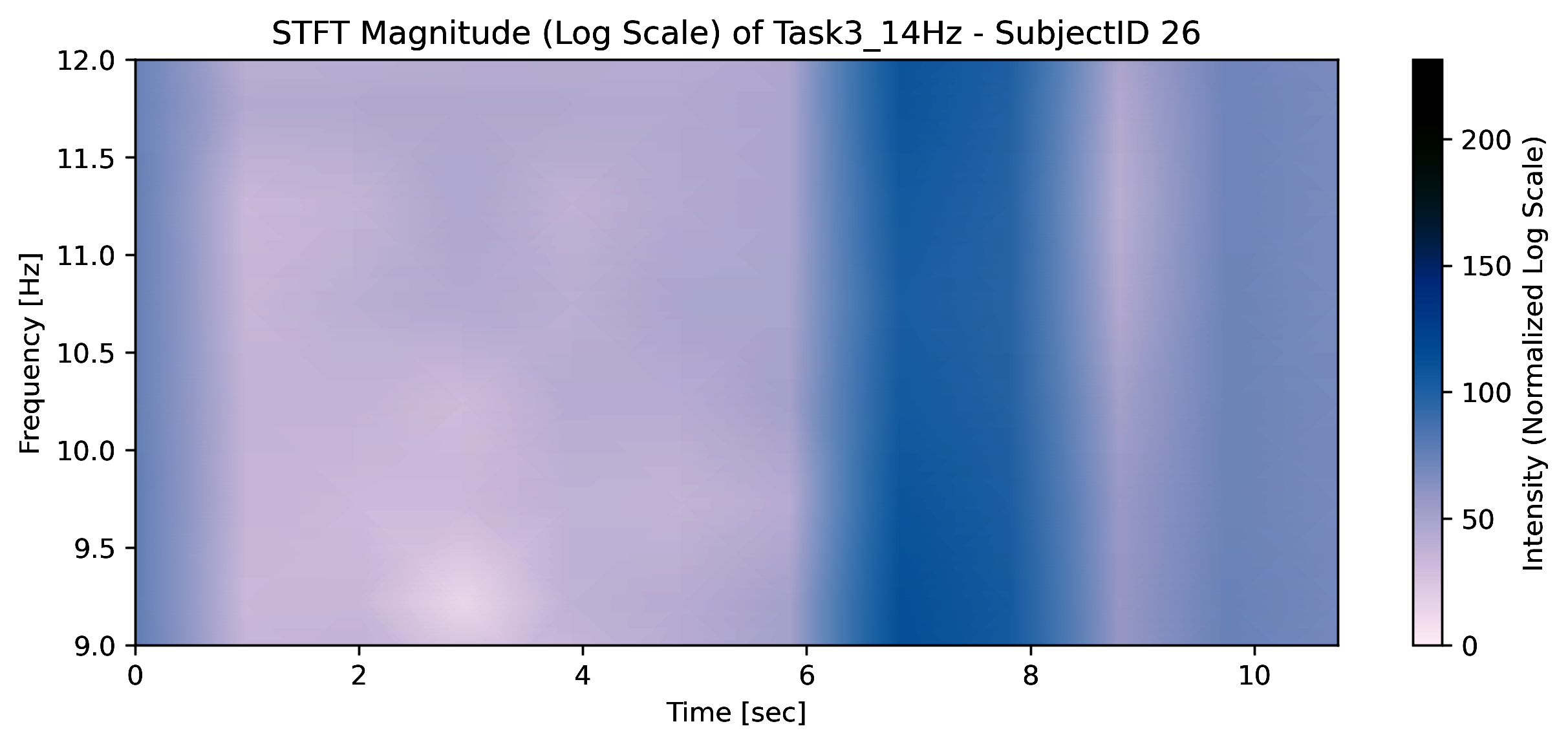

Supplement: Supplementary file 1 [file sensors-26-00157-s001.zip › STFT Images/PFG Images/Task 1-7 Images/S10 Task 3 ID_26.png]

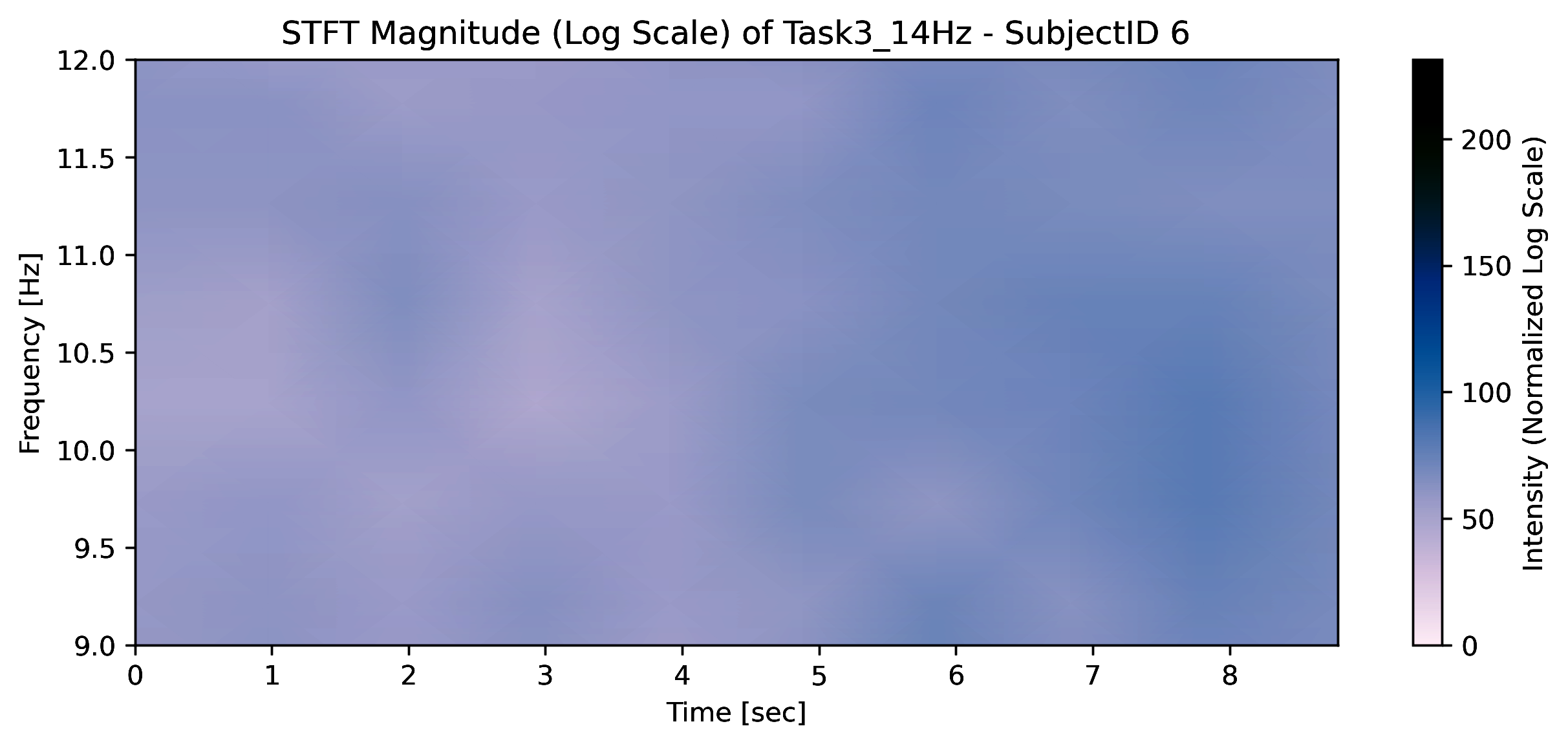

Supplement: Supplementary file 1 [file sensors-26-00157-s001.zip › STFT Images/PFG Images/Task 1-7 Images/S10 Task 3 ID_6.png]

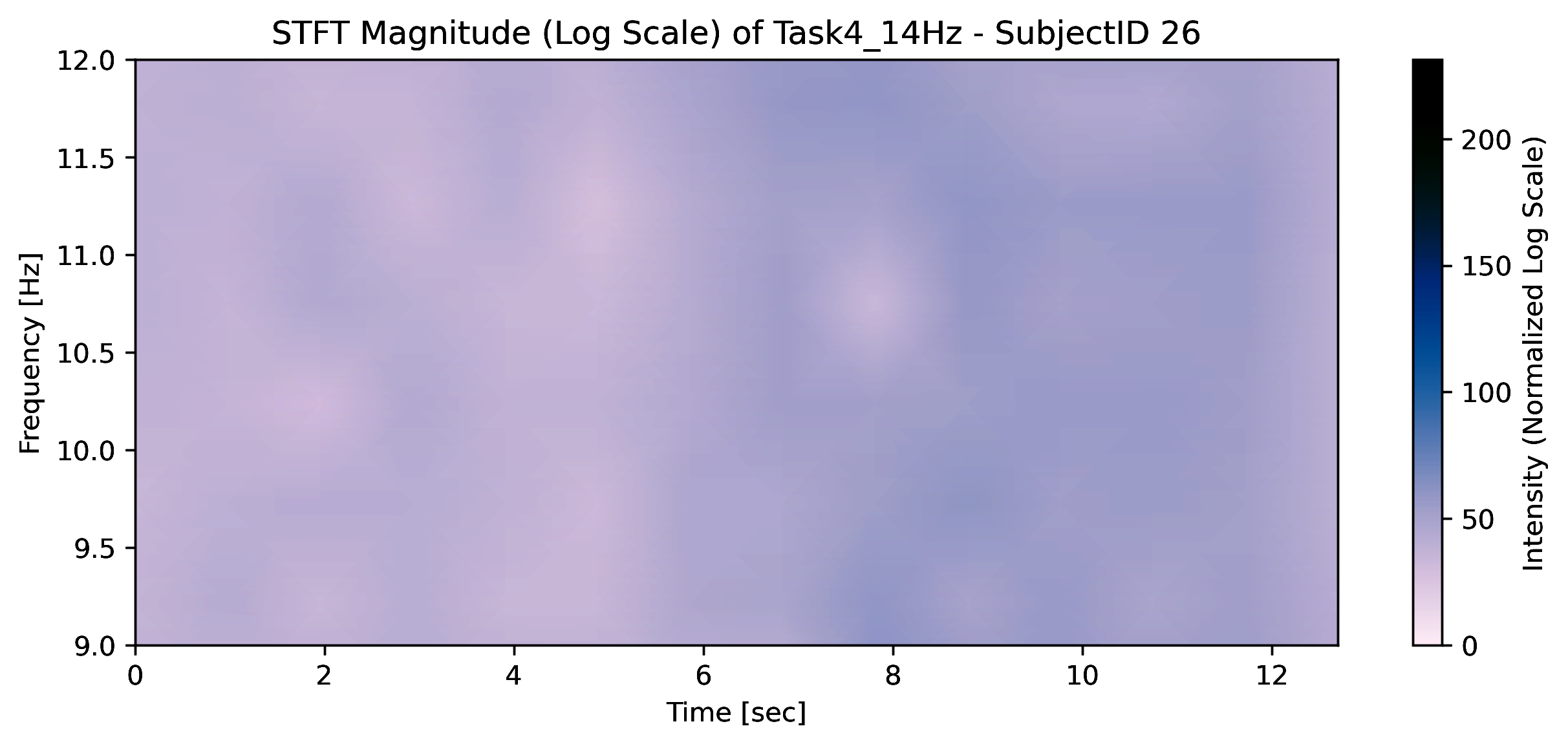

Supplement: Supplementary file 1 [file sensors-26-00157-s001.zip › STFT Images/PFG Images/Task 1-7 Images/S10 Task 4 ID_26.png]

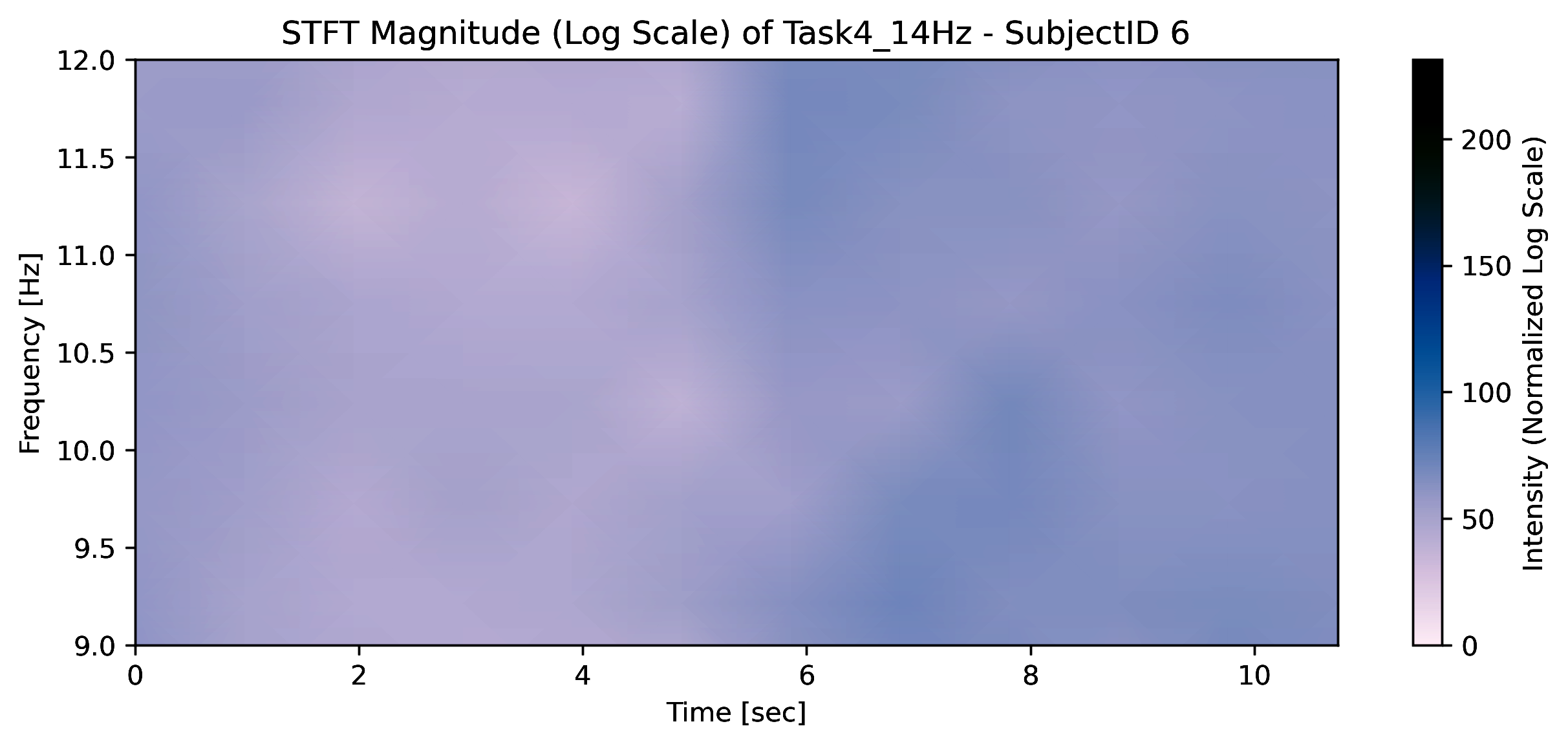

Supplement: Supplementary file 1 [file sensors-26-00157-s001.zip › STFT Images/PFG Images/Task 1-7 Images/S10 Task 4 ID_6.png]

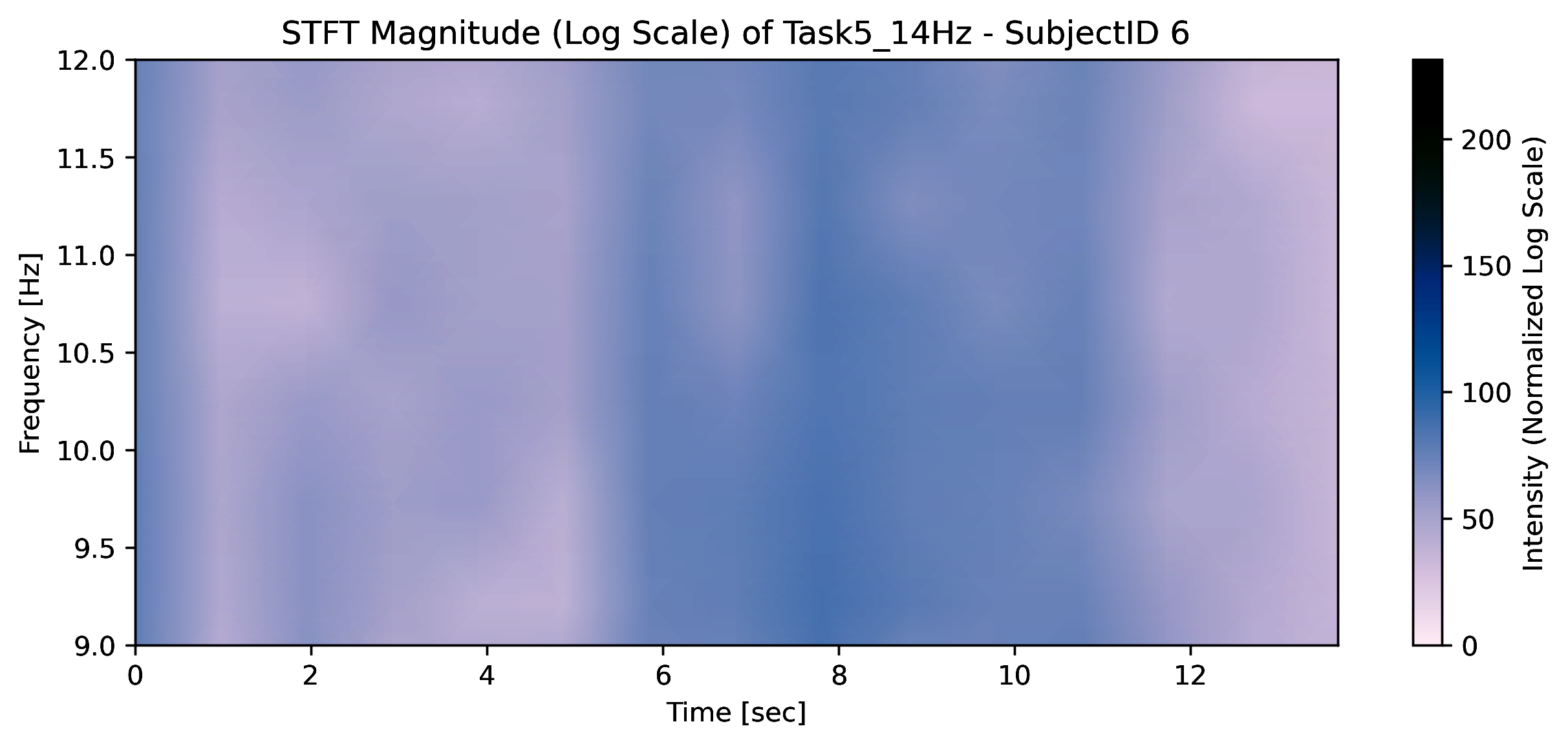

Supplement: Supplementary file 1 [file sensors-26-00157-s001.zip › STFT Images/PFG Images/Task 1-7 Images/S10 Task 5 D_6.png]

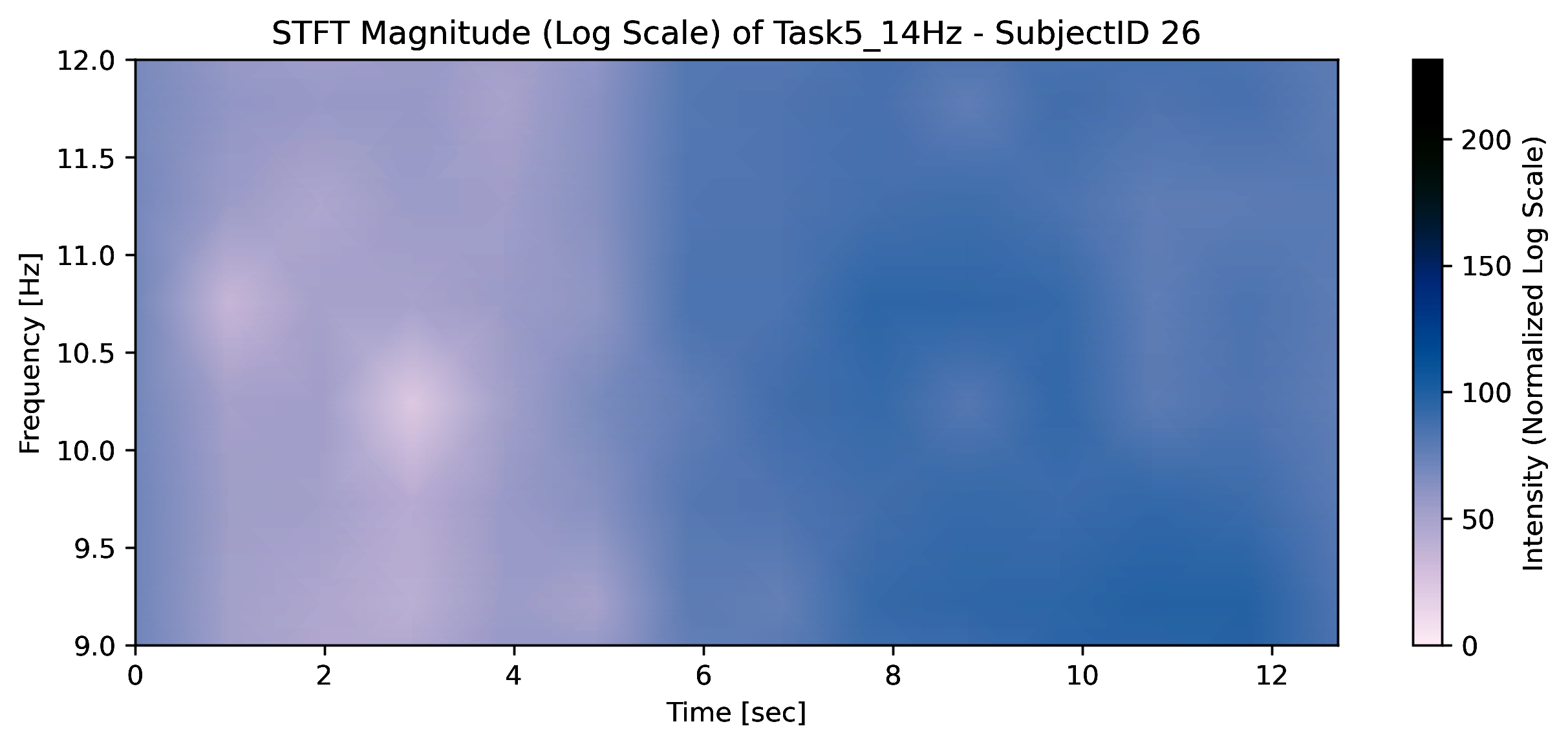

Supplement: Supplementary file 1 [file sensors-26-00157-s001.zip › STFT Images/PFG Images/Task 1-7 Images/S10 Task 5 ID_26.png]

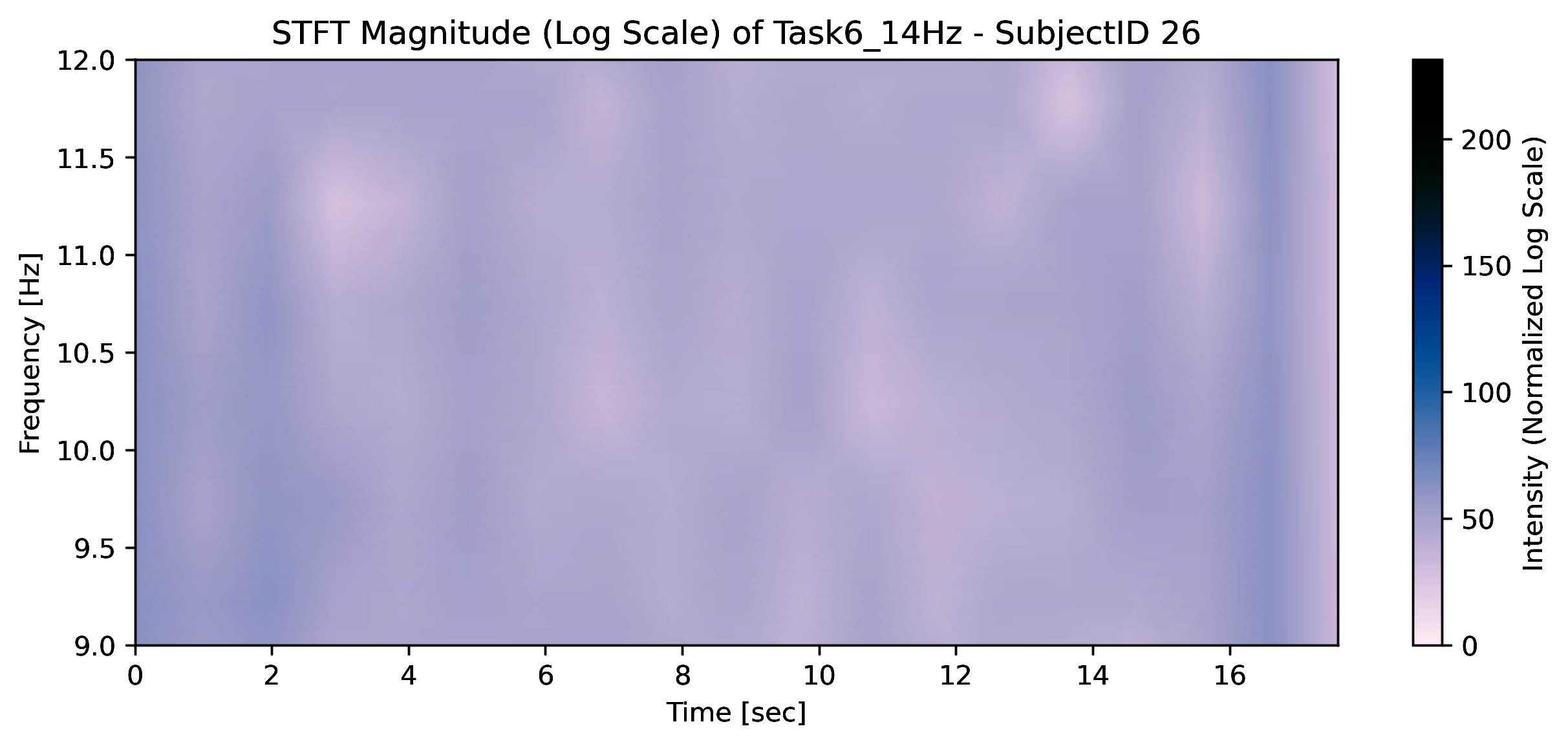

Supplement: Supplementary file 1 [file sensors-26-00157-s001.zip › STFT Images/PFG Images/Task 1-7 Images/S10 Task 6 ID_26.png]

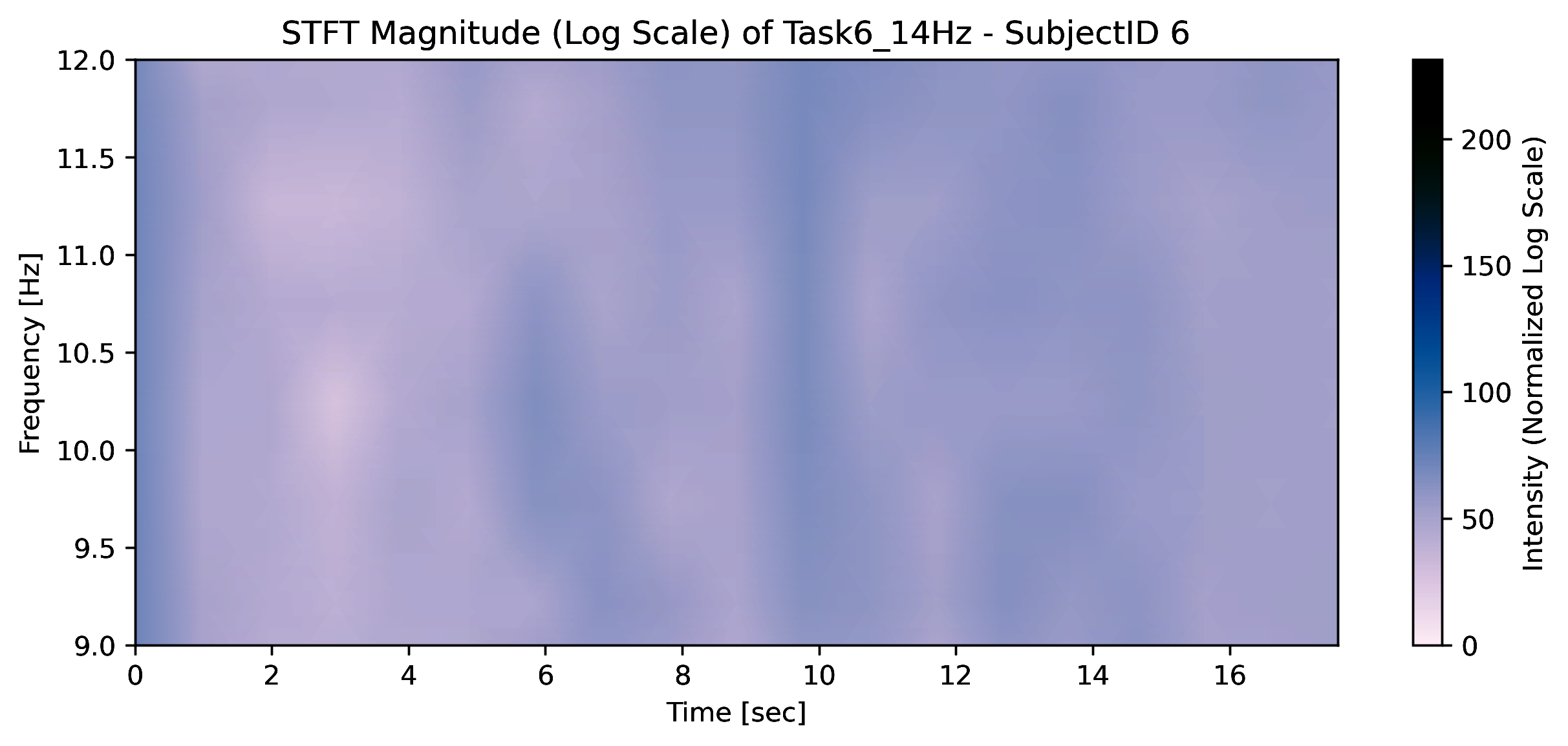

Supplement: Supplementary file 1 [file sensors-26-00157-s001.zip › STFT Images/PFG Images/Task 1-7 Images/S10 Task 6 ID_6.png]

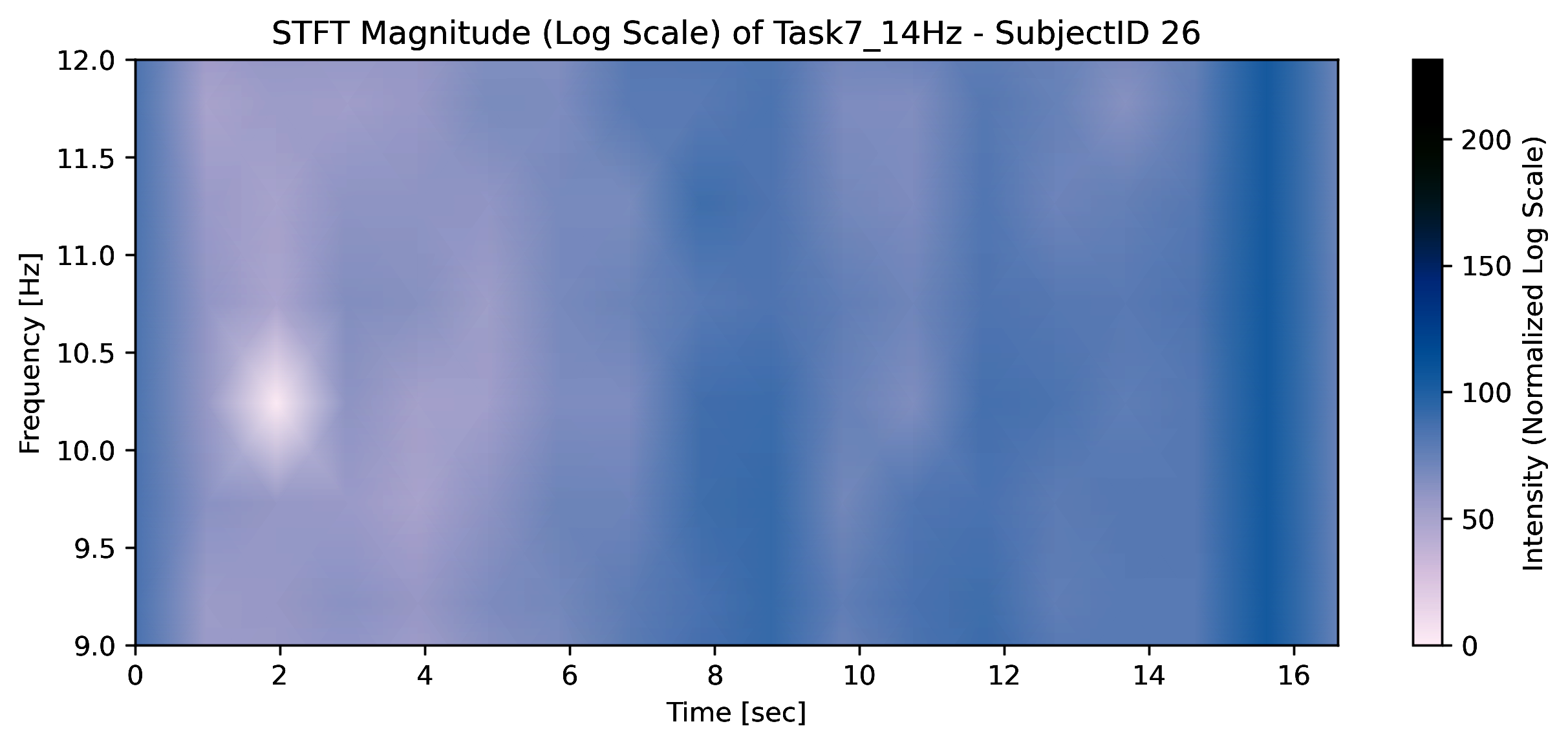

Supplement: Supplementary file 1 [file sensors-26-00157-s001.zip › STFT Images/PFG Images/Task 1-7 Images/S10 Task 7 ID_26.png]

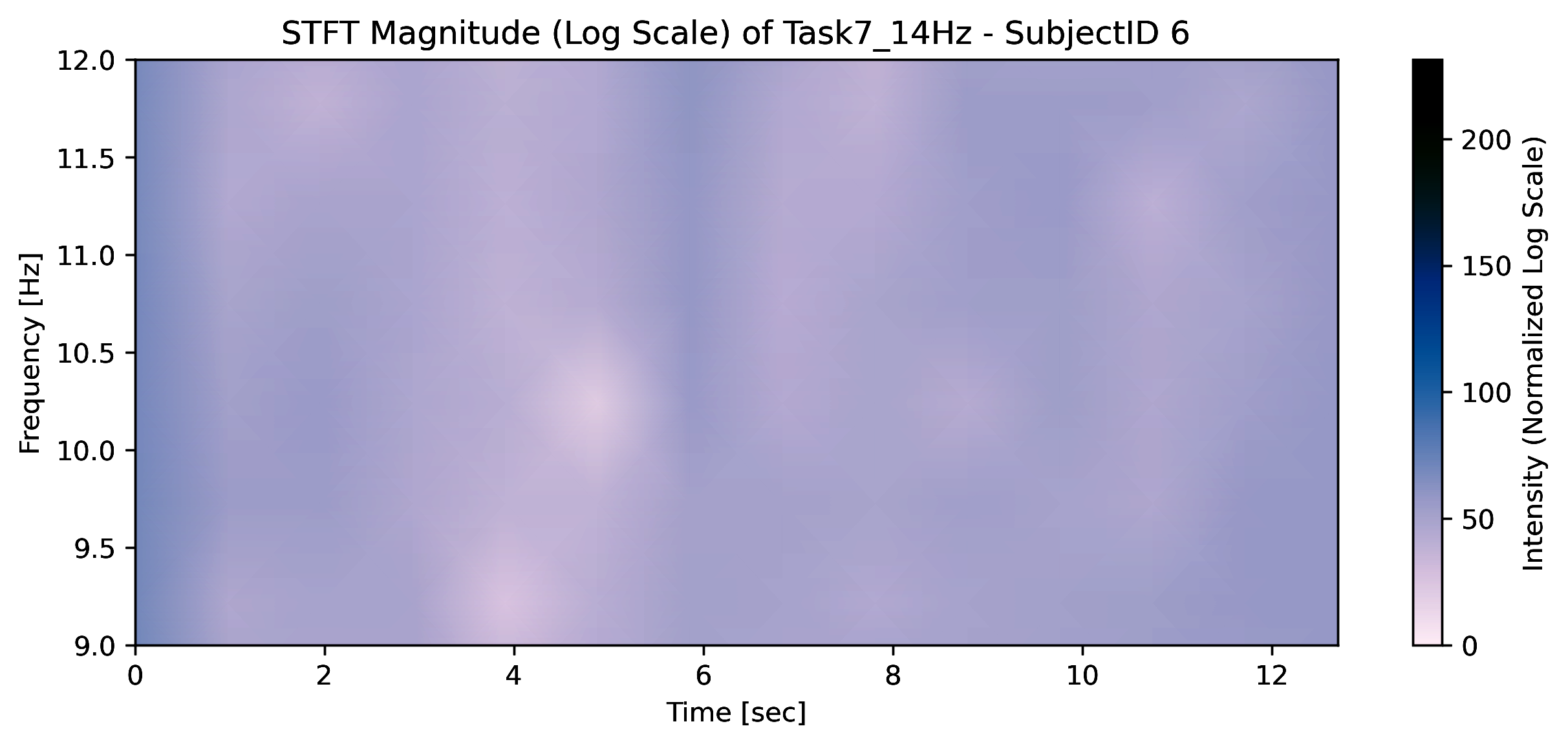

Supplement: Supplementary file 1 [file sensors-26-00157-s001.zip › STFT Images/PFG Images/Task 1-7 Images/S10 Task 7 ID_6.png]

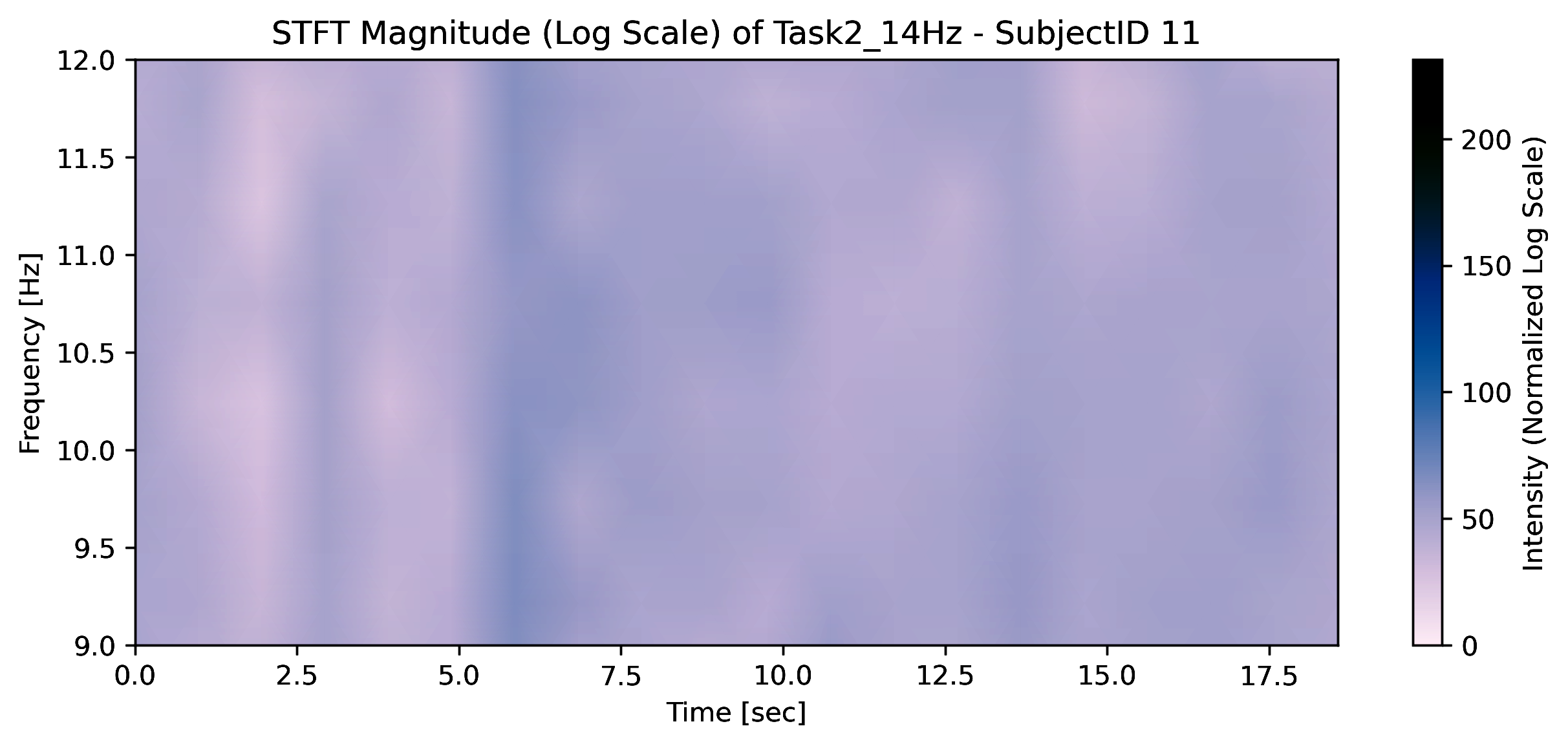

Supplement: Supplementary file 1 [file sensors-26-00157-s001.zip › STFT Images/PFG Images/Task 2 Images/S8 ID_11.png]

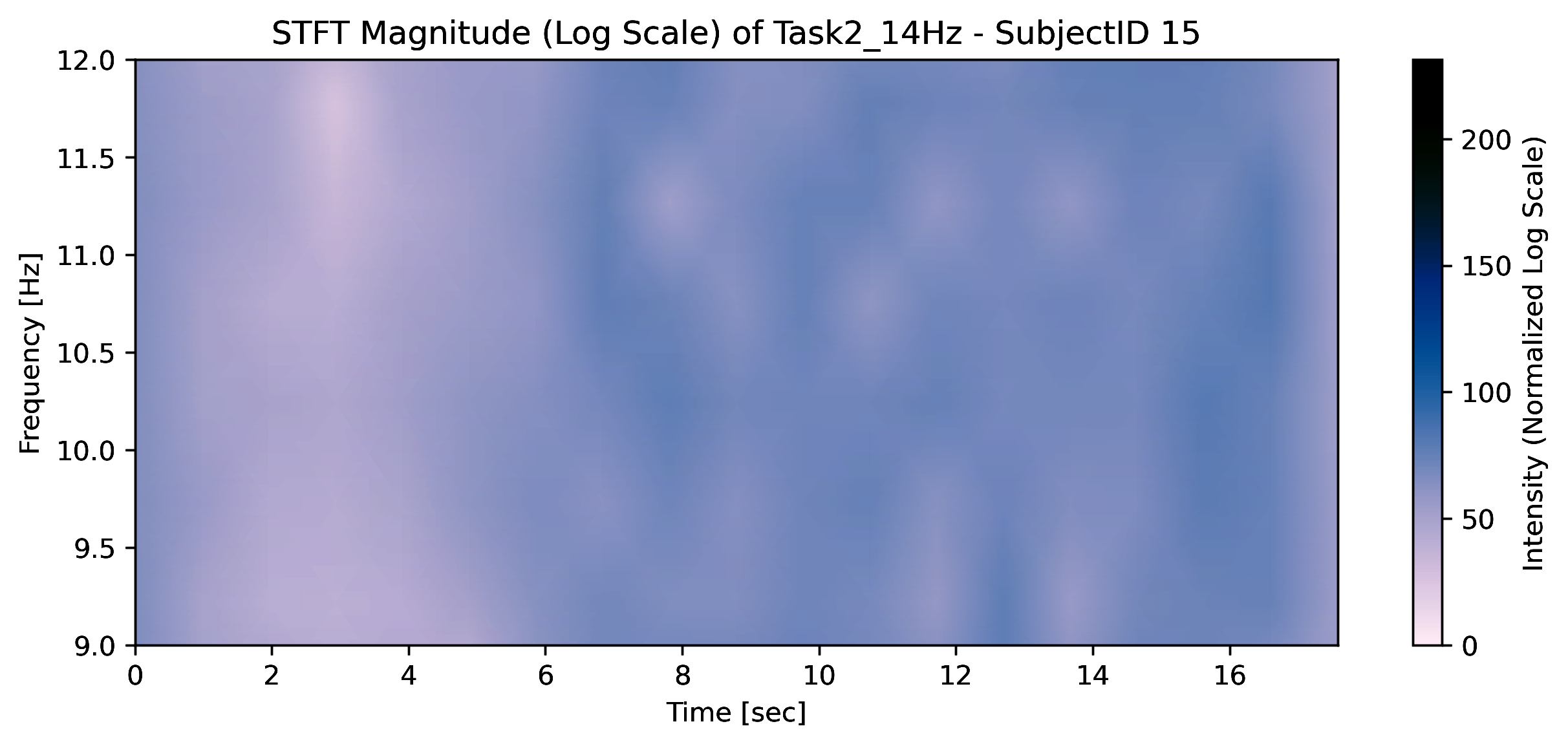

Supplement: Supplementary file 1 [file sensors-26-00157-s001.zip › STFT Images/PFG Images/Task 2 Images/S8 ID_15.png]

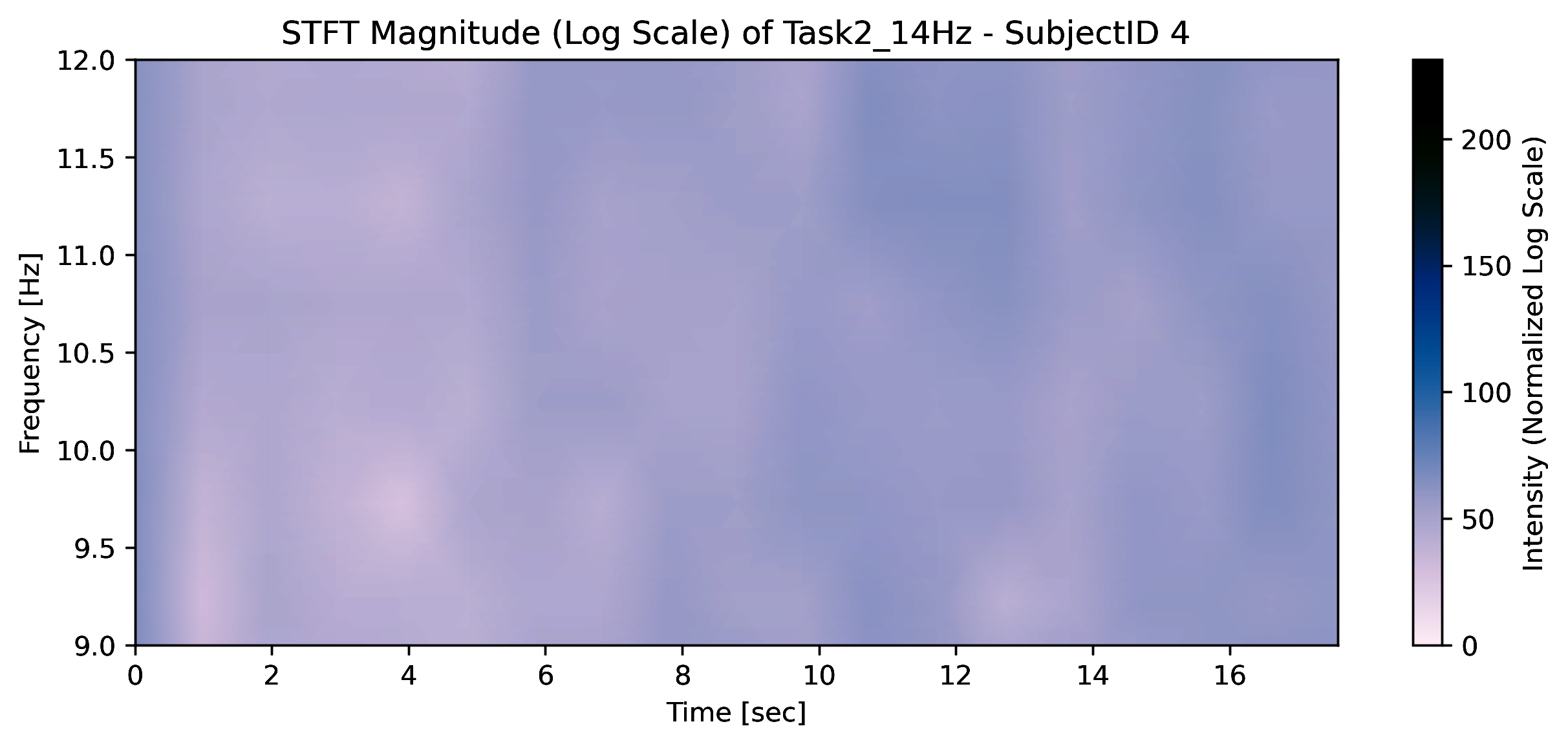

Supplement: Supplementary file 1 [file sensors-26-00157-s001.zip › STFT Images/PFG Images/Task 2 Images/S8 ID_4.png]

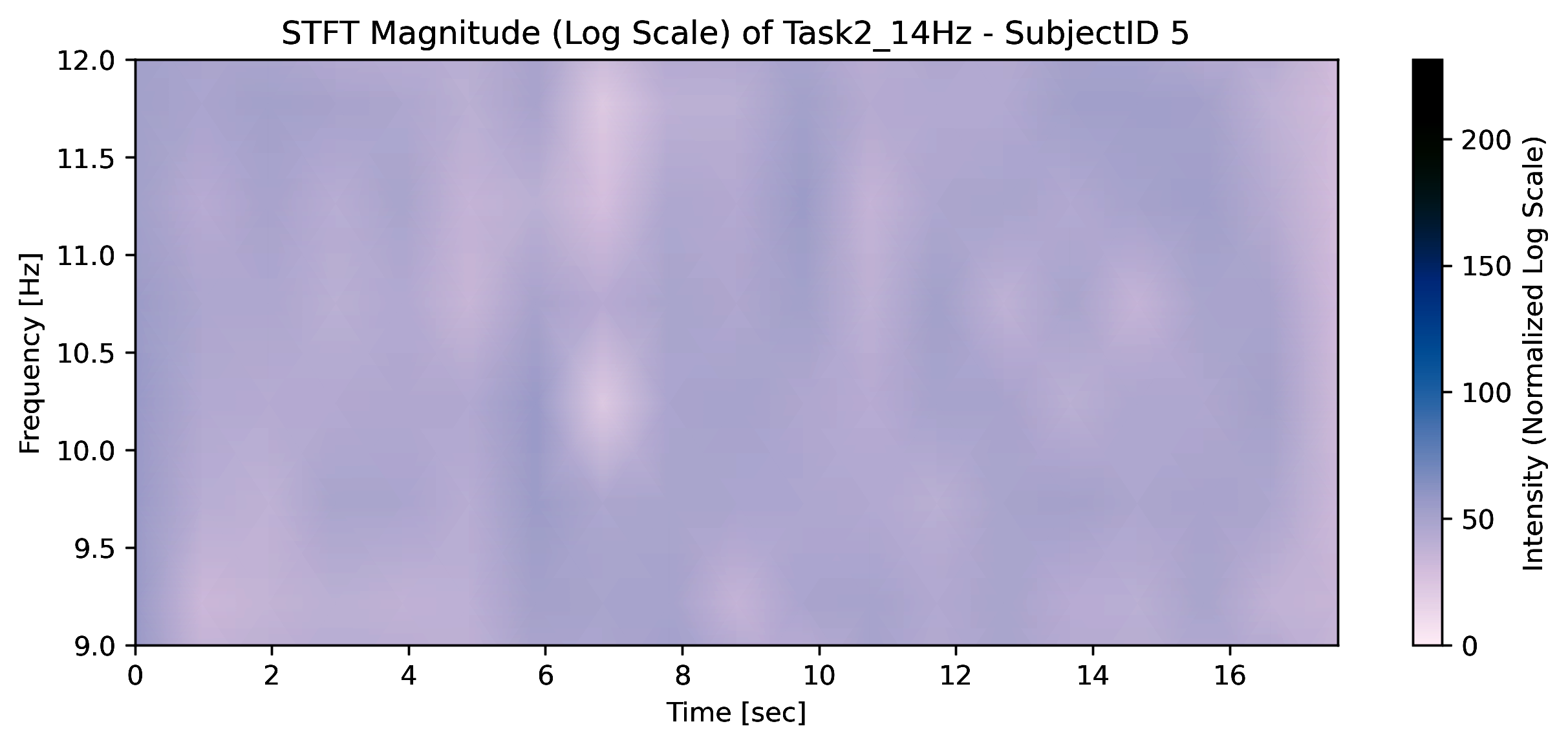

Supplement: Supplementary file 1 [file sensors-26-00157-s001.zip › STFT Images/PFG Images/Task 2 Images/S8 ID_5.png]

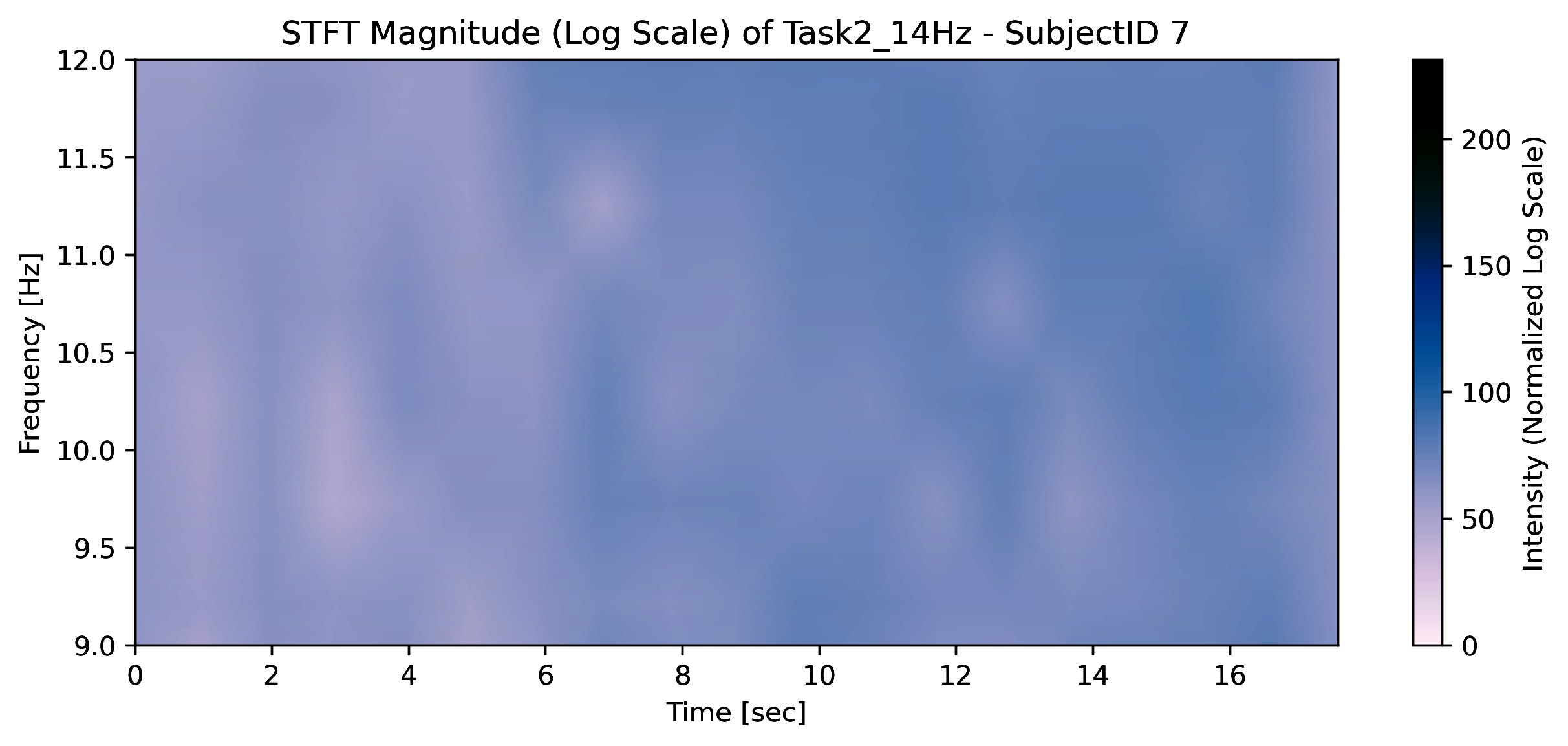

Supplement: Supplementary file 1 [file sensors-26-00157-s001.zip › STFT Images/PFG Images/Task 2 Images/S8 ID_7.png]

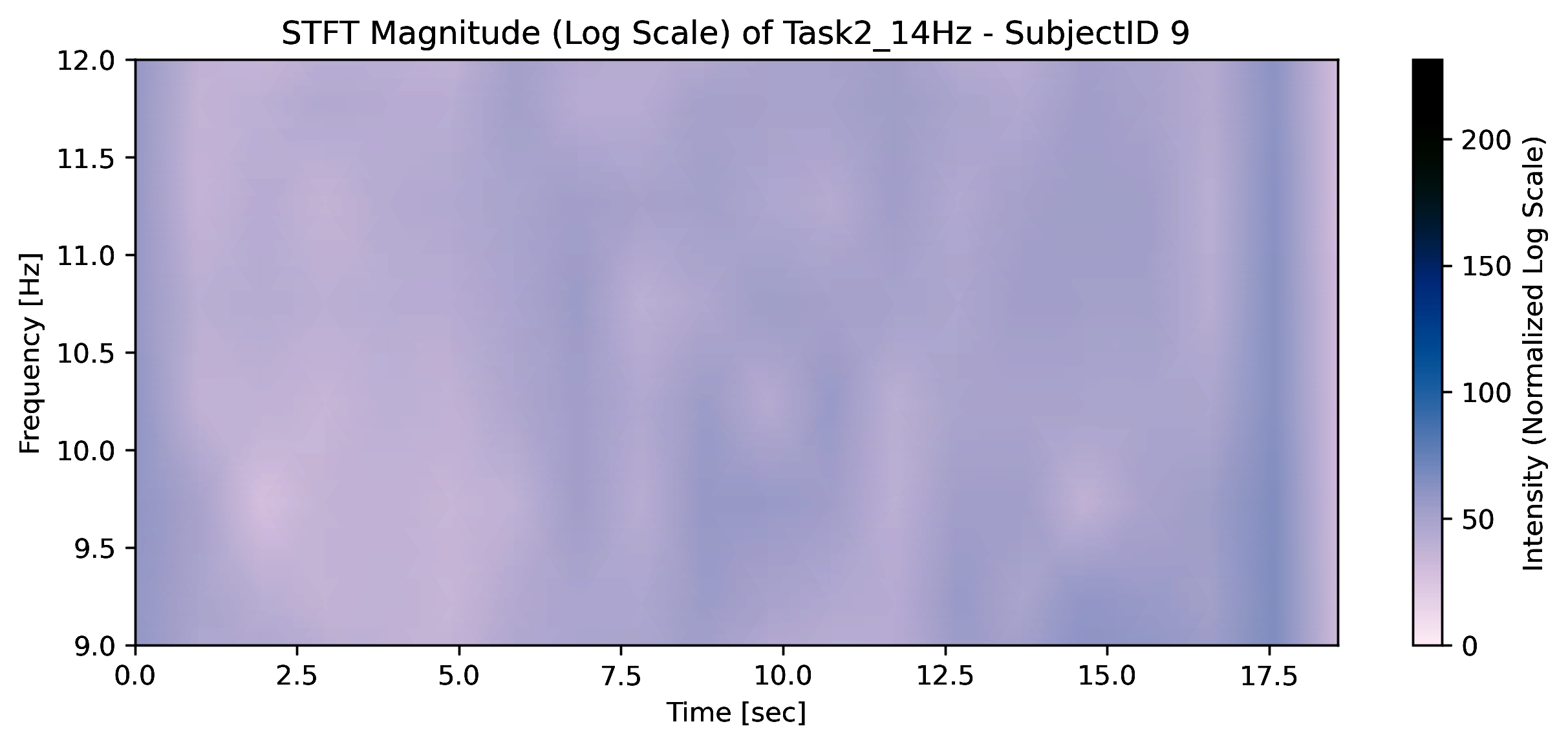

Supplement: Supplementary file 1 [file sensors-26-00157-s001.zip › STFT Images/PFG Images/Task 2 Images/S8 ID_9.png]

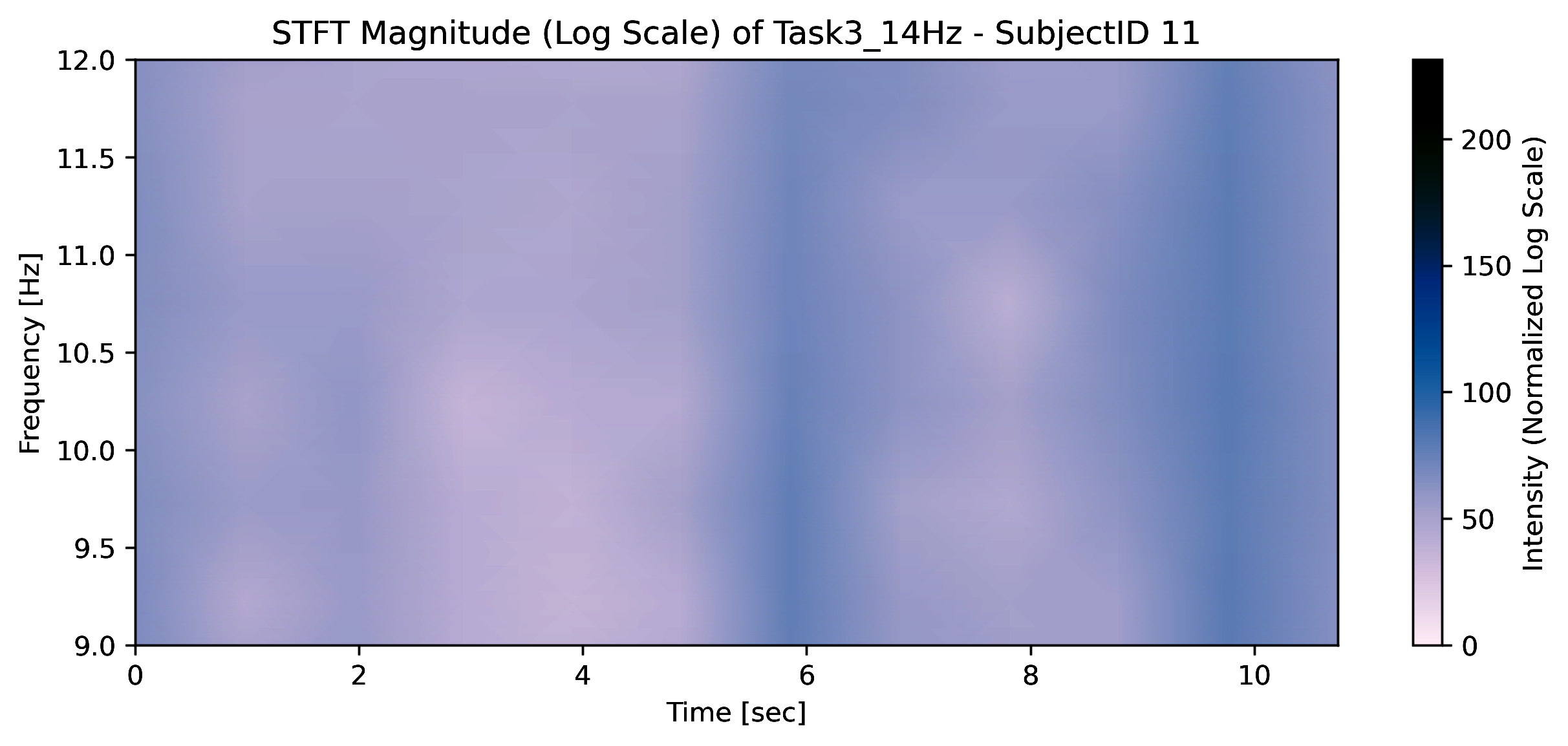

Supplement: Supplementary file 1 [file sensors-26-00157-s001.zip › STFT Images/PFG Images/Task 3 Images/S9 ID_11.png]

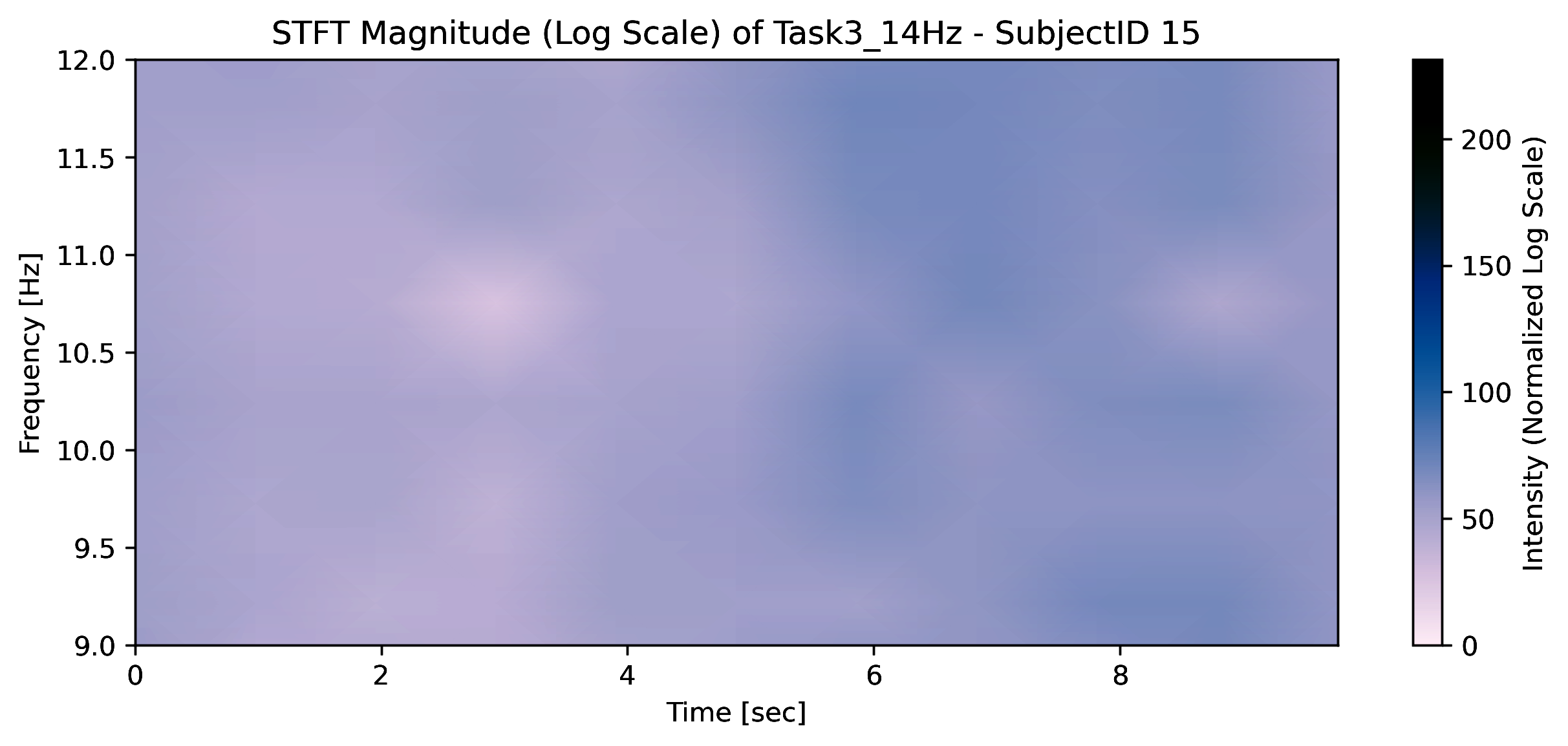

Supplement: Supplementary file 1 [file sensors-26-00157-s001.zip › STFT Images/PFG Images/Task 3 Images/S9 ID_15.png]

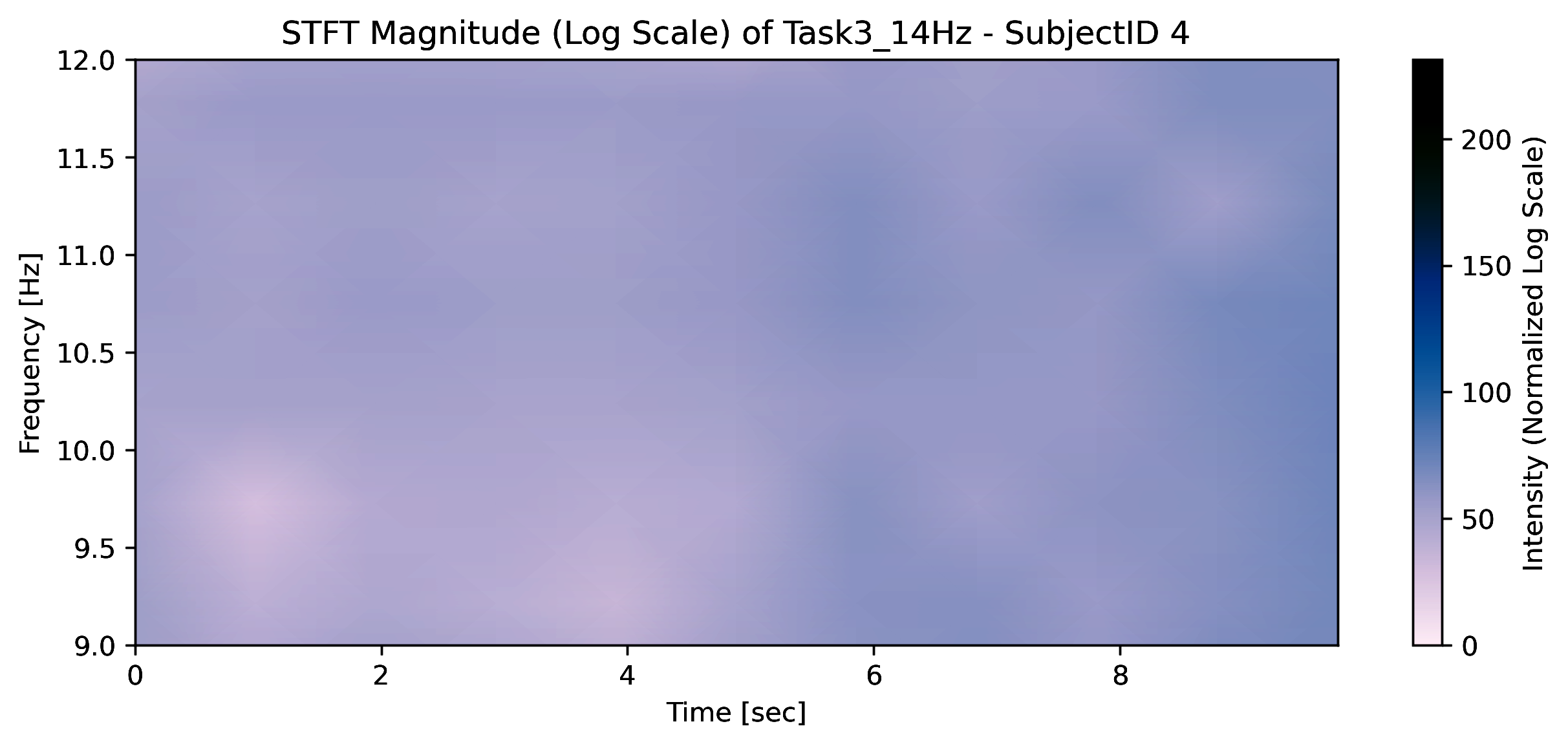

Supplement: Supplementary file 1 [file sensors-26-00157-s001.zip › STFT Images/PFG Images/Task 3 Images/S9 ID_4.png]

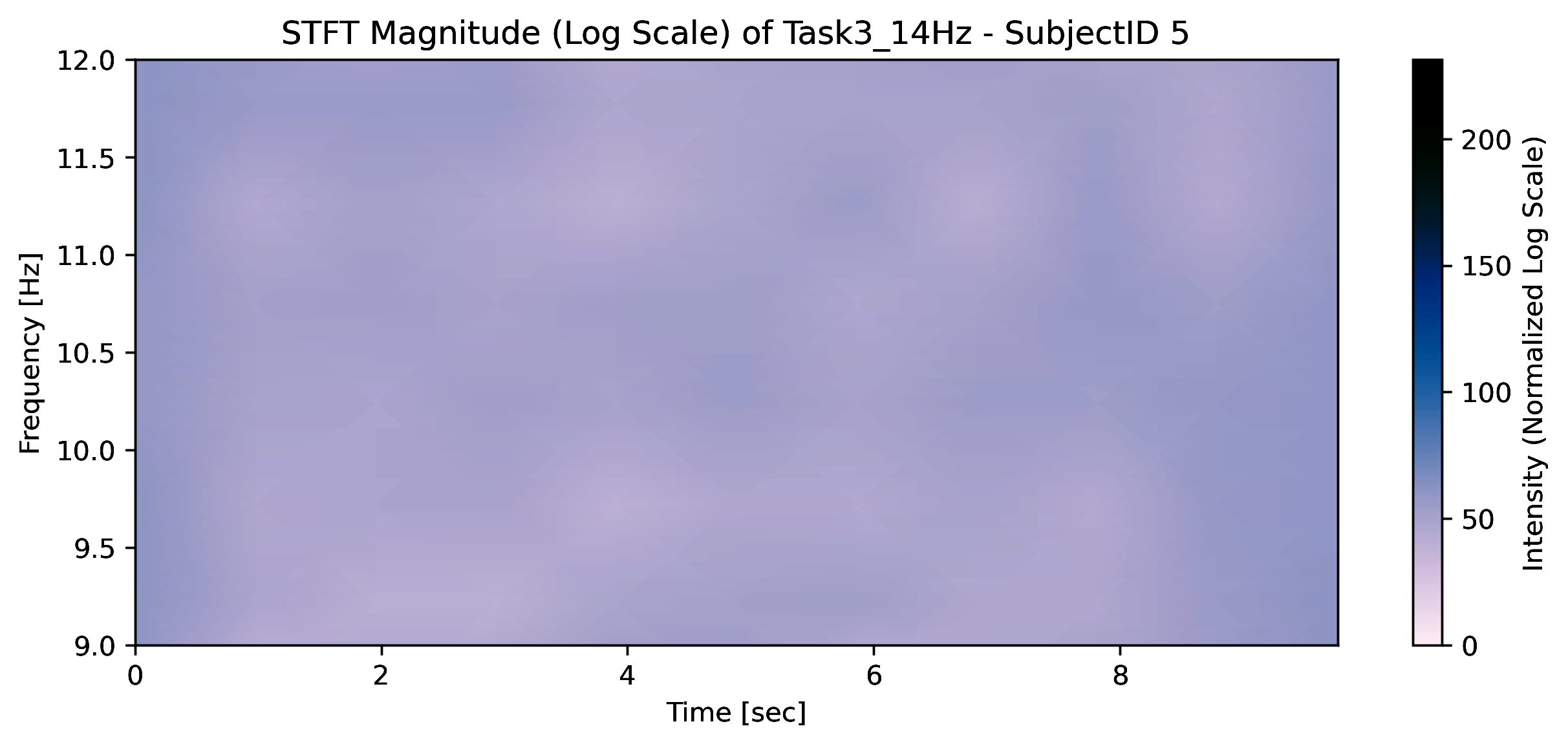

Supplement: Supplementary file 1 [file sensors-26-00157-s001.zip › STFT Images/PFG Images/Task 3 Images/S9 ID_5.png]

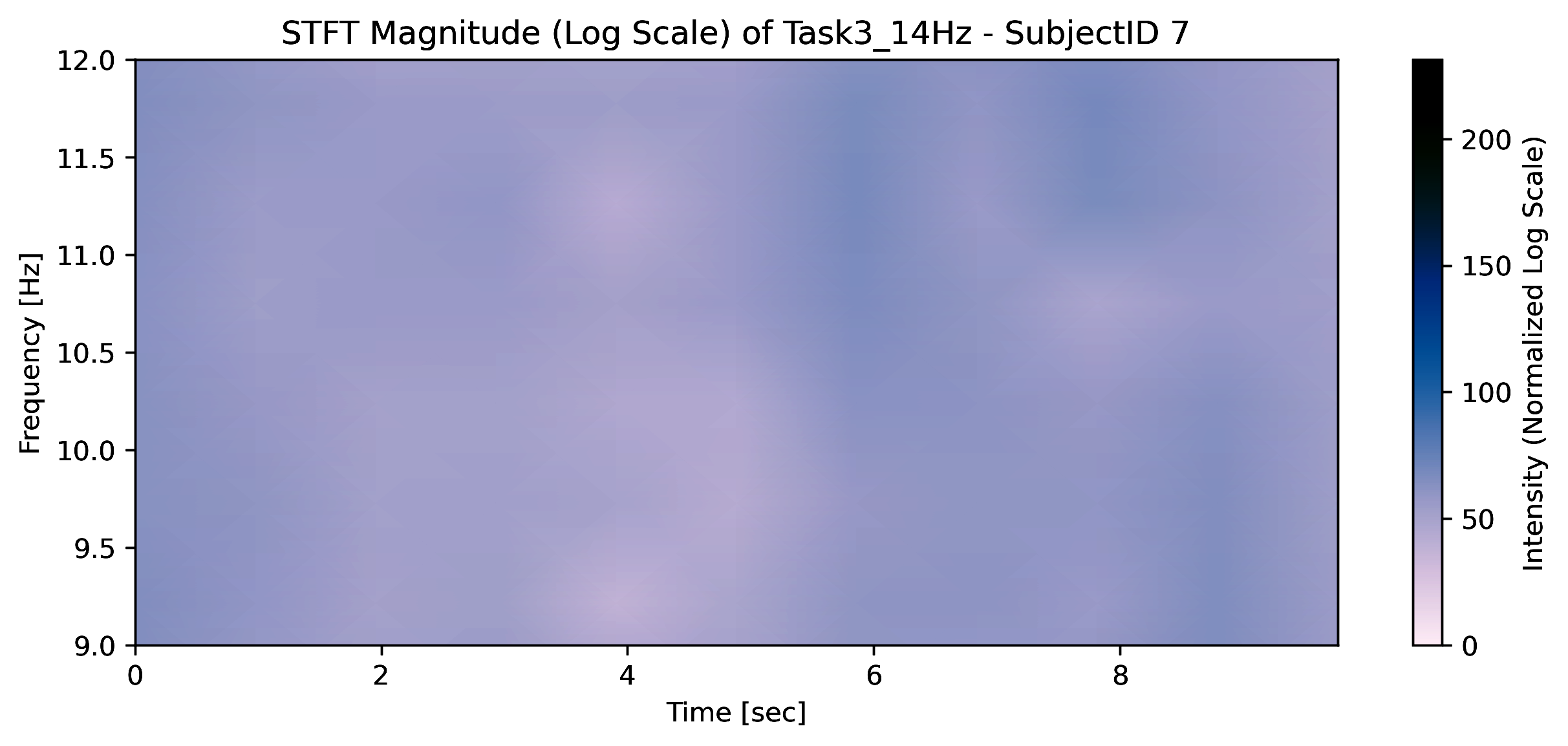

Supplement: Supplementary file 1 [file sensors-26-00157-s001.zip › STFT Images/PFG Images/Task 3 Images/S9 ID_7.png]

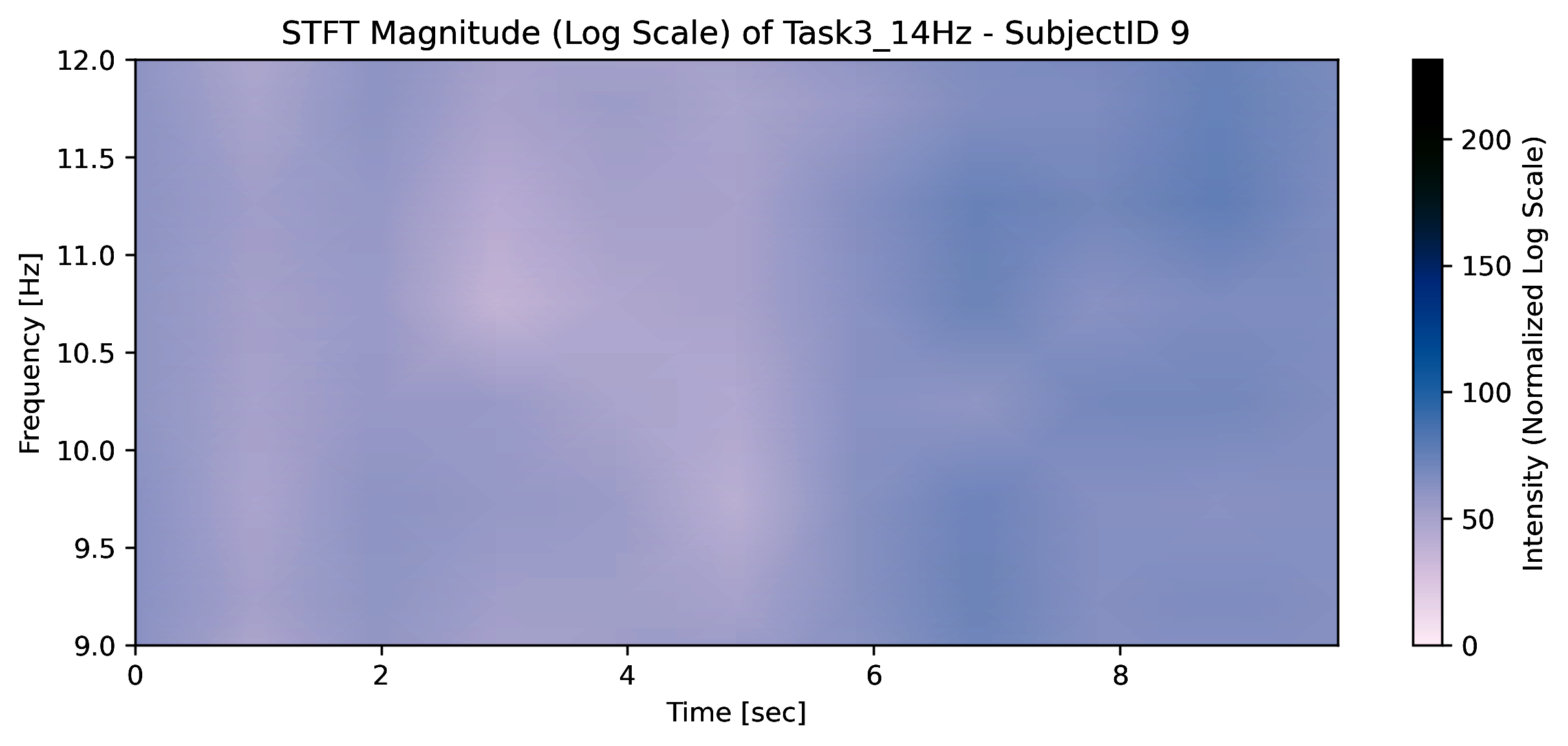

Supplement: Supplementary file 1 [file sensors-26-00157-s001.zip › STFT Images/PFG Images/Task 3 Images/S9 ID_9.png]

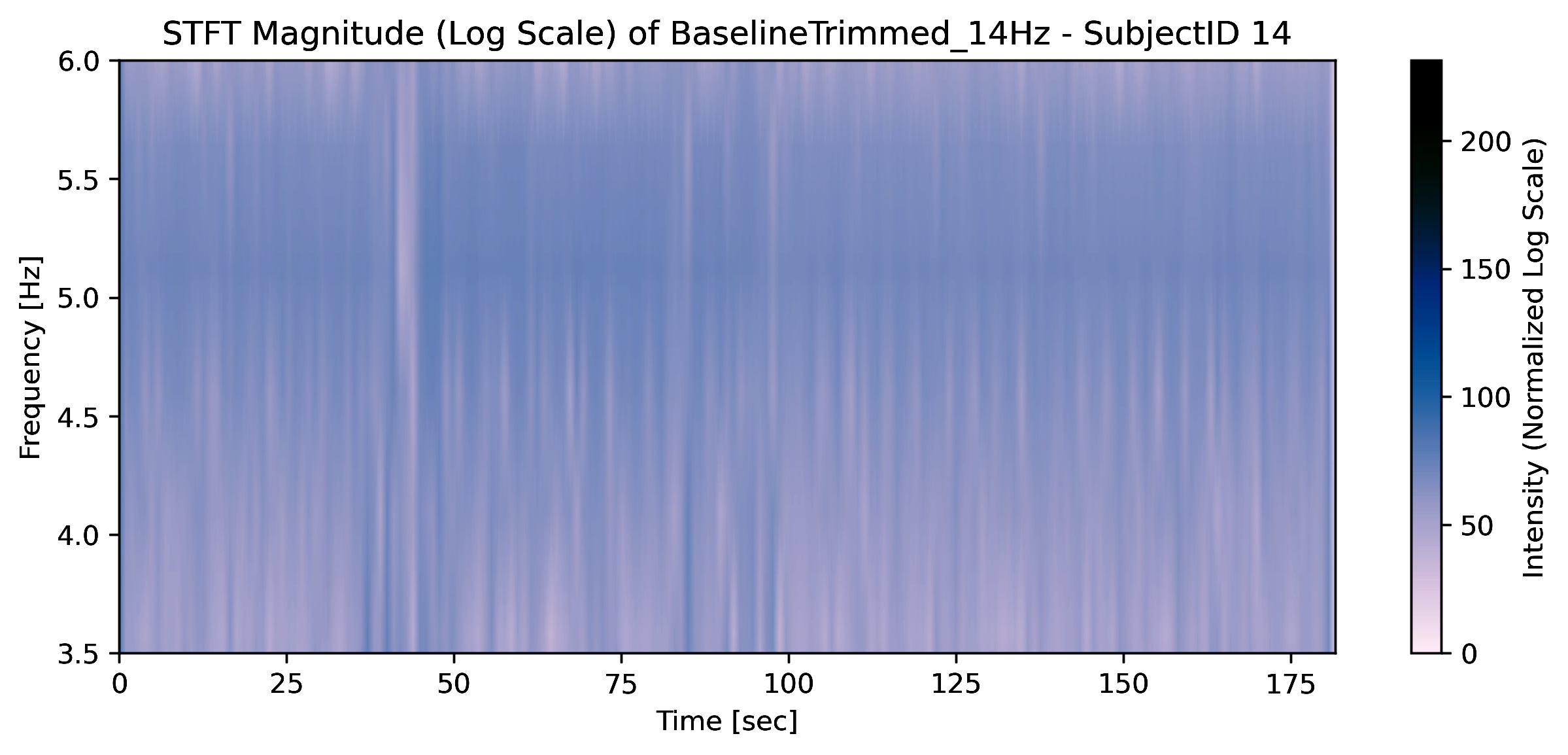

Supplement: Supplementary file 1 [file sensors-26-00157-s001.zip › STFT Images/RFG Images/Baseline Images/Figure S11A ID 14.png]

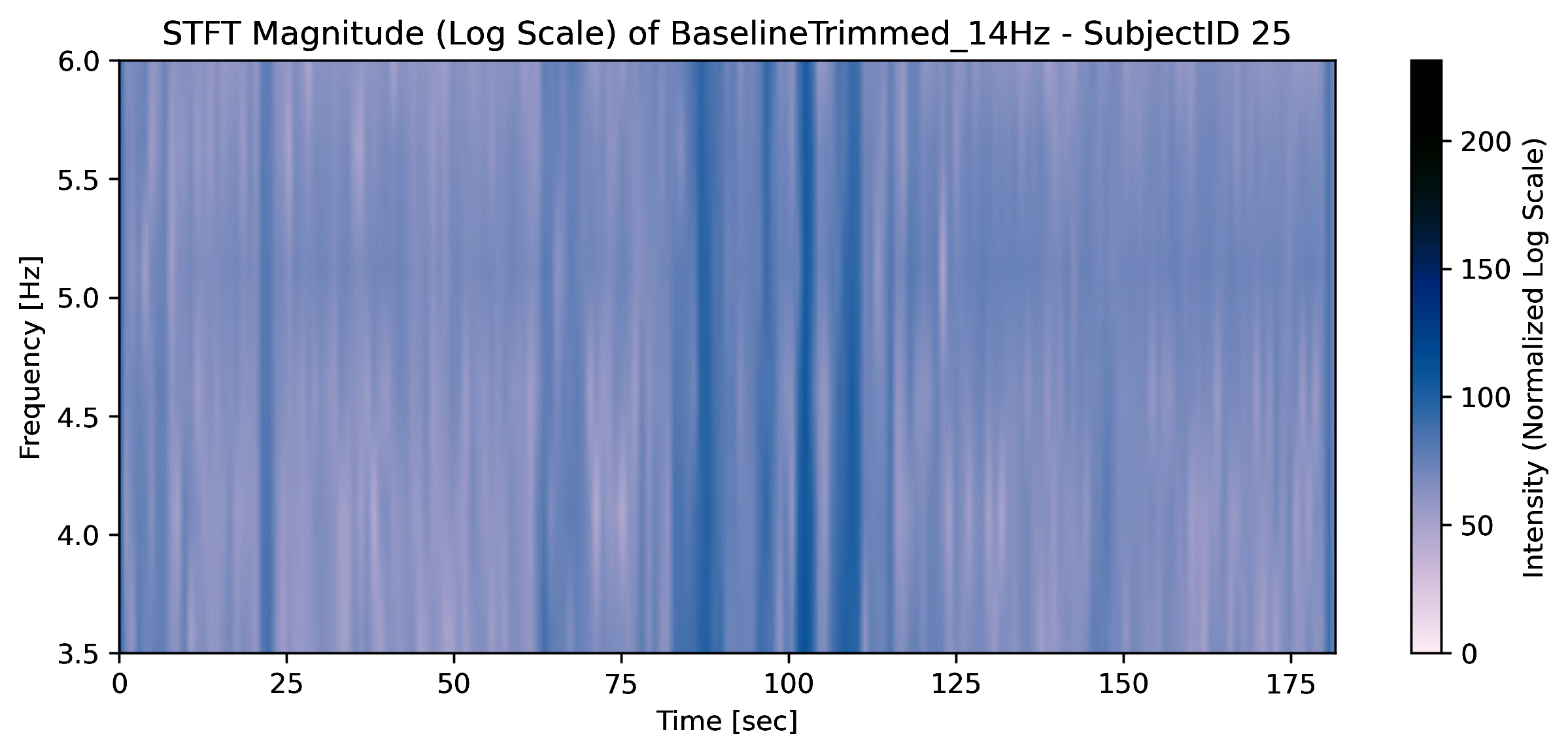

Supplement: Supplementary file 1 [file sensors-26-00157-s001.zip › STFT Images/RFG Images/Baseline Images/Figure S11B ID 25.png]

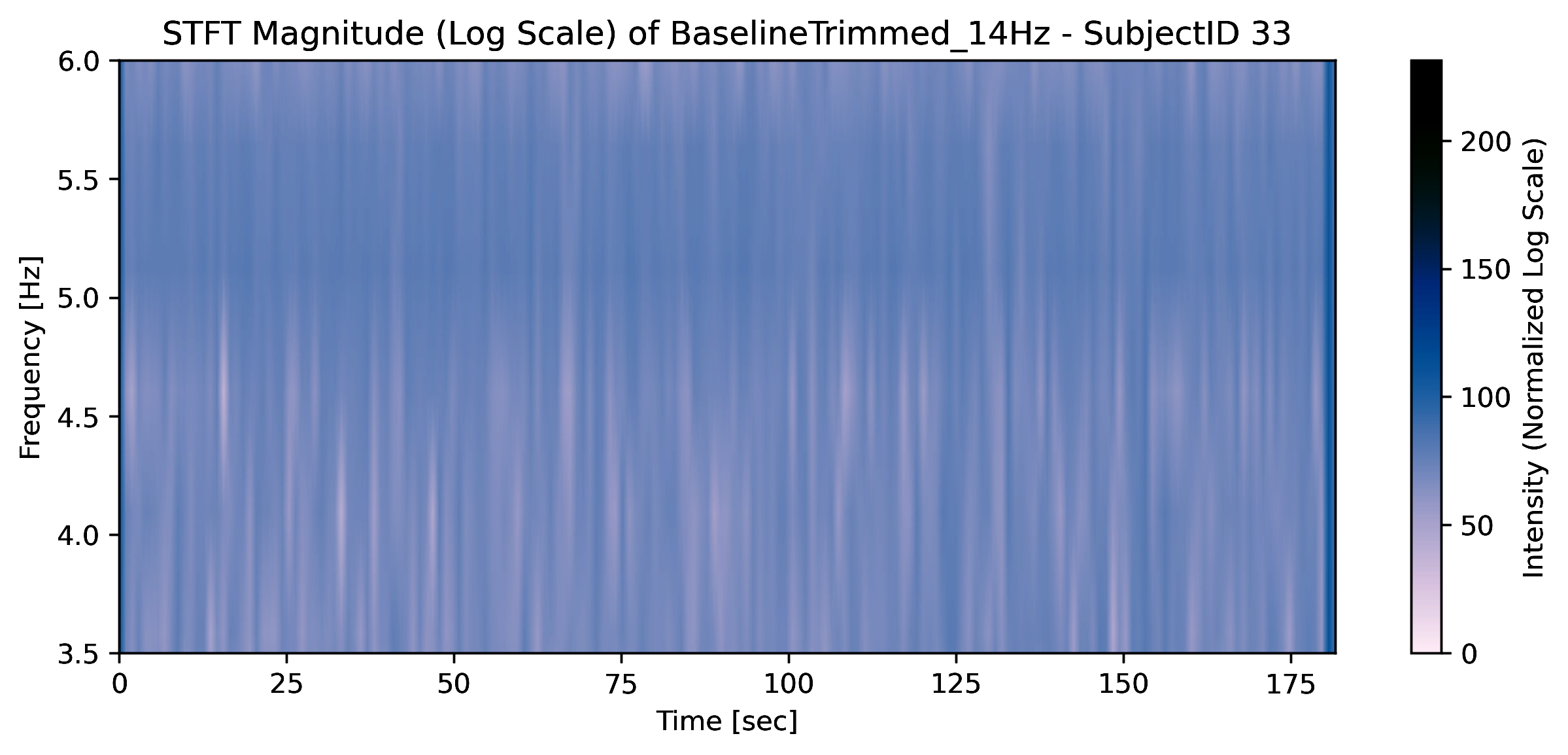

Supplement: Supplementary file 1 [file sensors-26-00157-s001.zip › STFT Images/RFG Images/Baseline Images/Figure S11C ID 33.png]

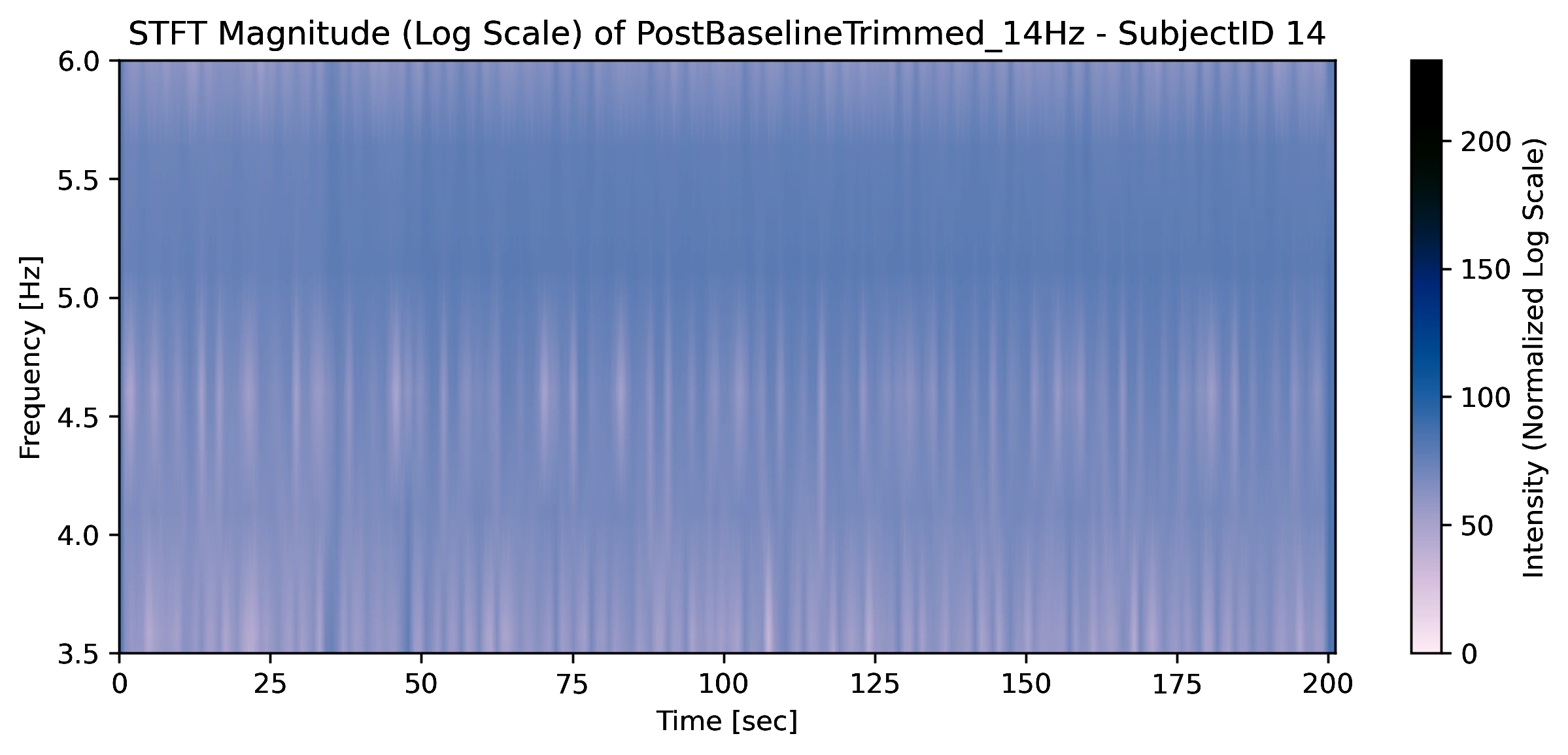

Supplement: Supplementary file 1 [file sensors-26-00157-s001.zip › STFT Images/RFG Images/PostBaseline Images/Figure S12A ID 14.png]

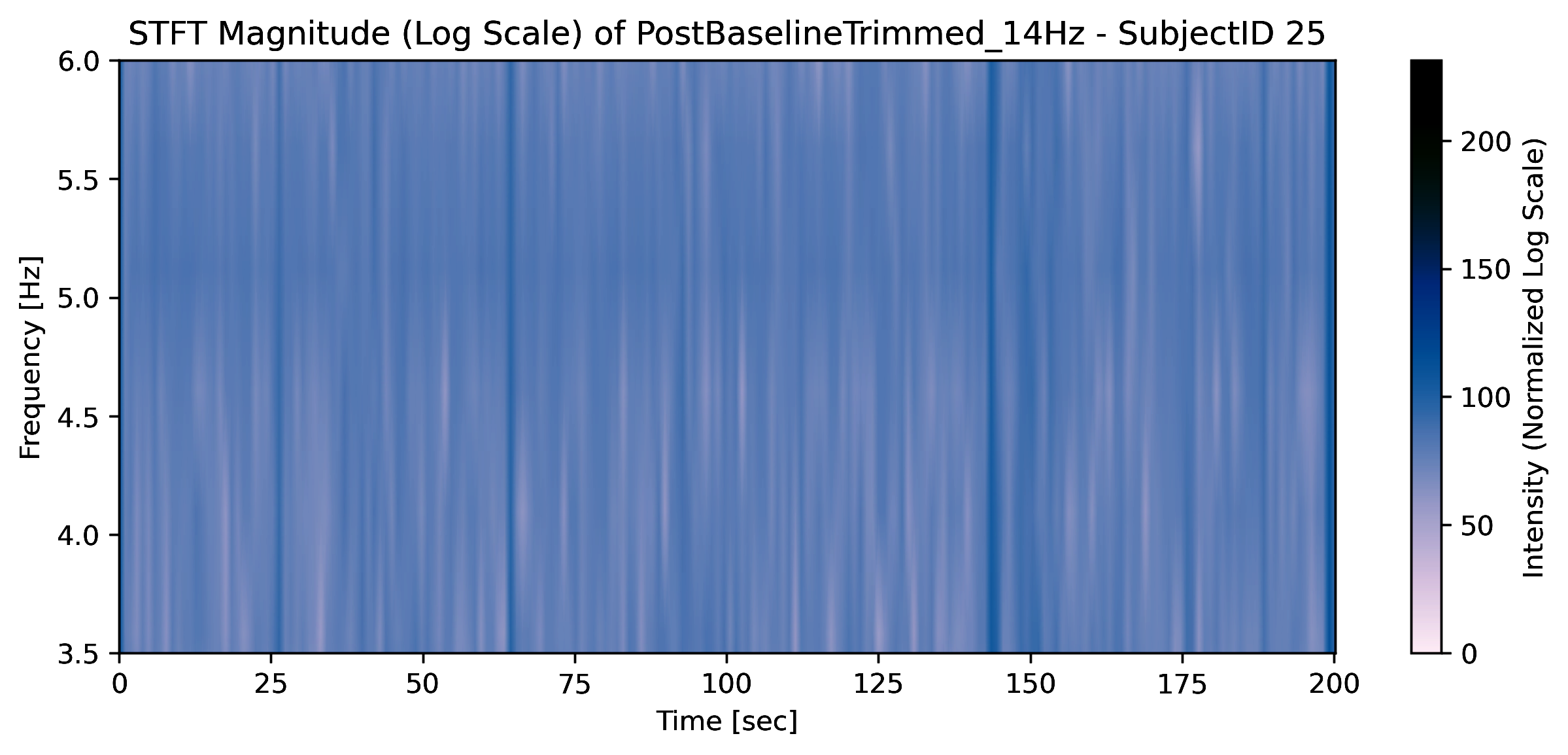

Supplement: Supplementary file 1 [file sensors-26-00157-s001.zip › STFT Images/RFG Images/PostBaseline Images/Figure S12B ID 25.png]

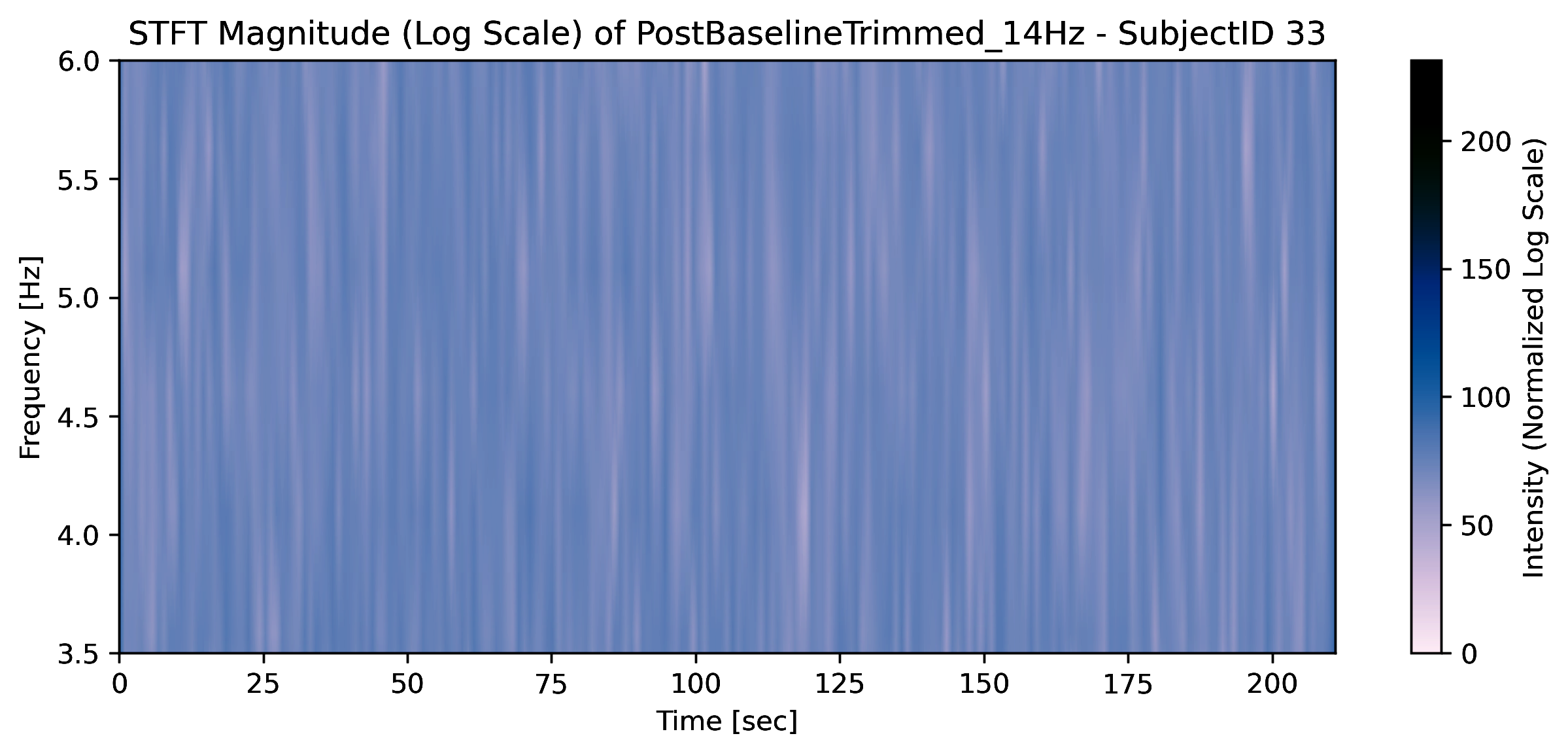

Supplement: Supplementary file 1 [file sensors-26-00157-s001.zip › STFT Images/RFG Images/PostBaseline Images/Figure S12C ID 33.png]

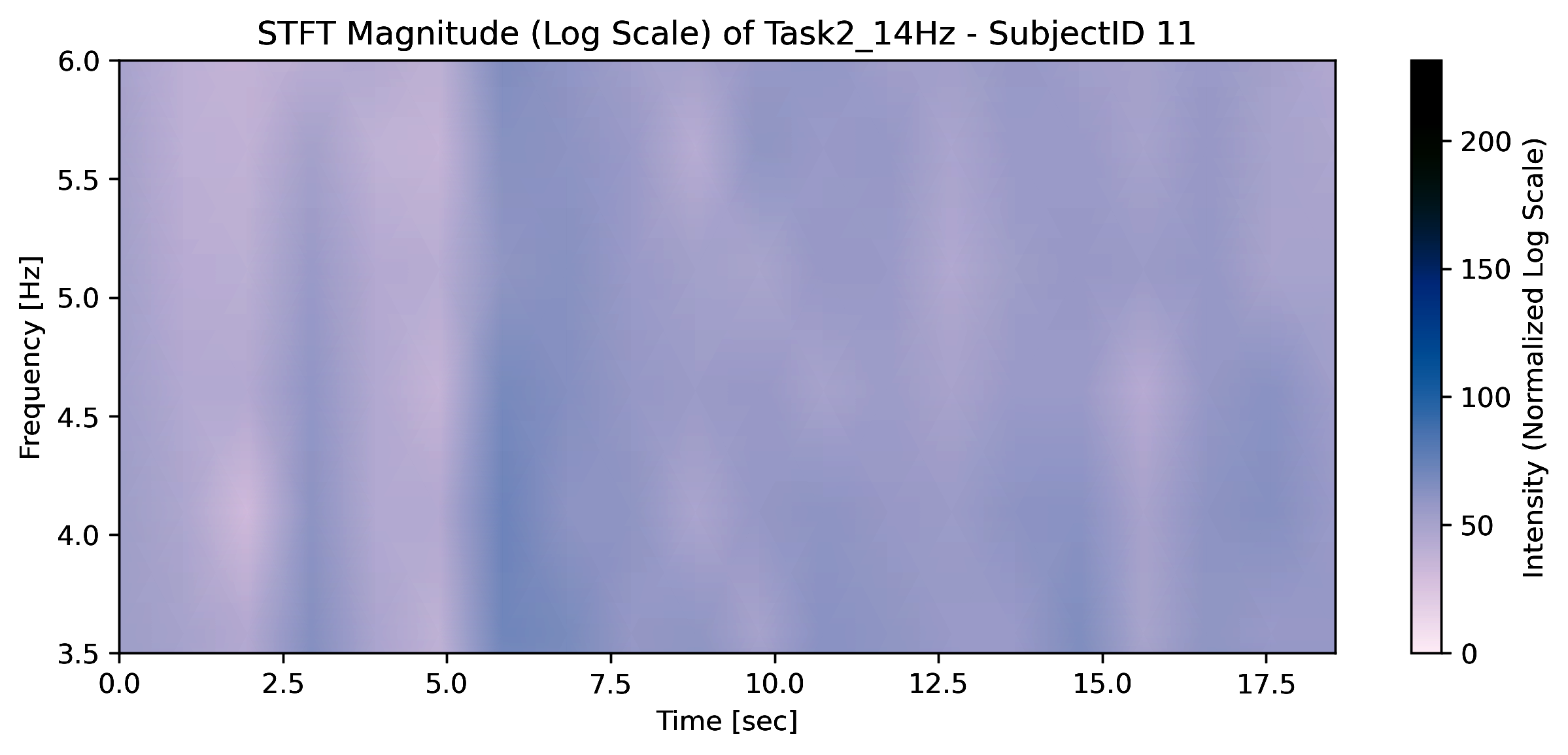

Supplement: Supplementary file 1 [file sensors-26-00157-s001.zip › STFT Images/RFG Images/Task 2 Images/Figure S13 ID 11.png]

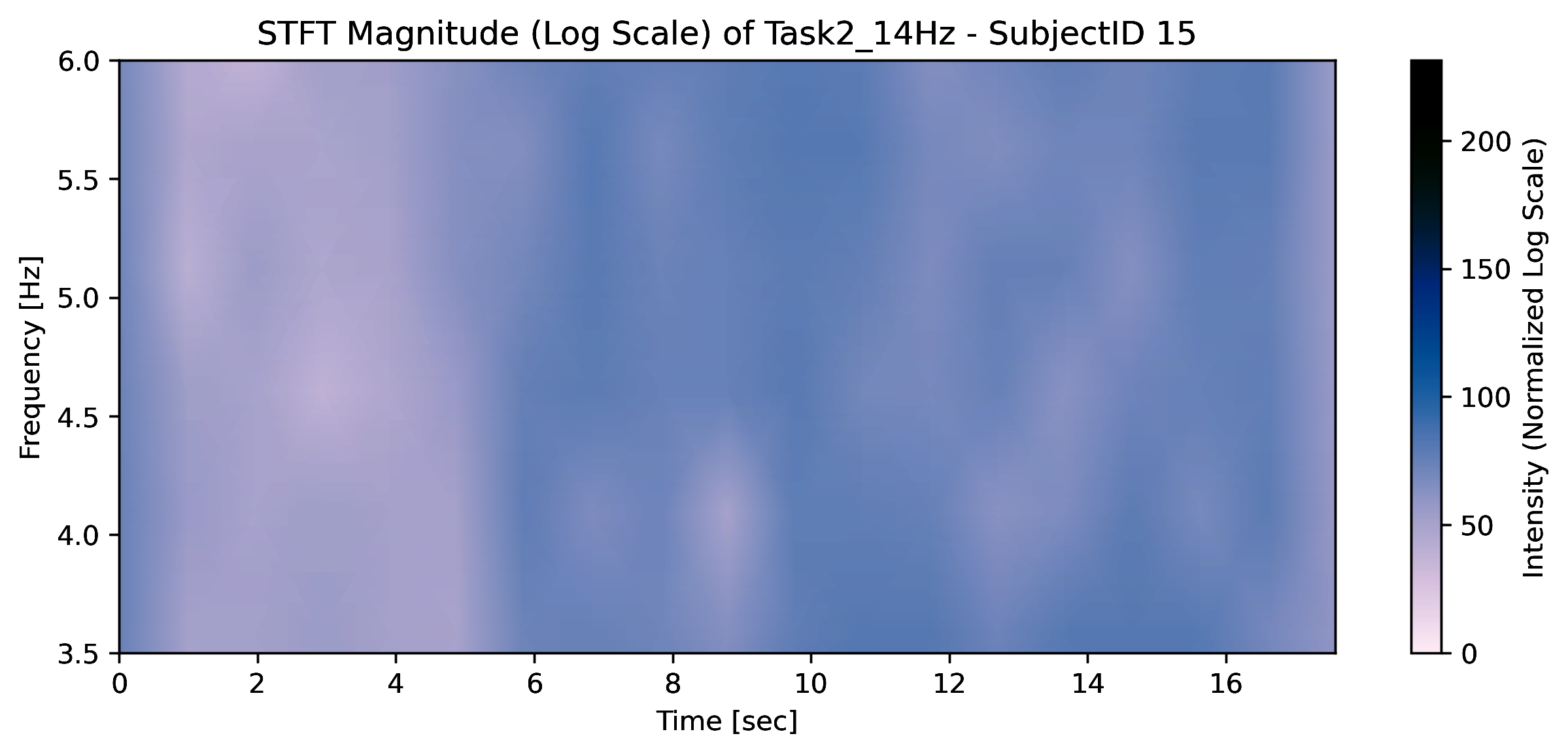

Supplement: Supplementary file 1 [file sensors-26-00157-s001.zip › STFT Images/RFG Images/Task 2 Images/Figure S13 ID 15.png]

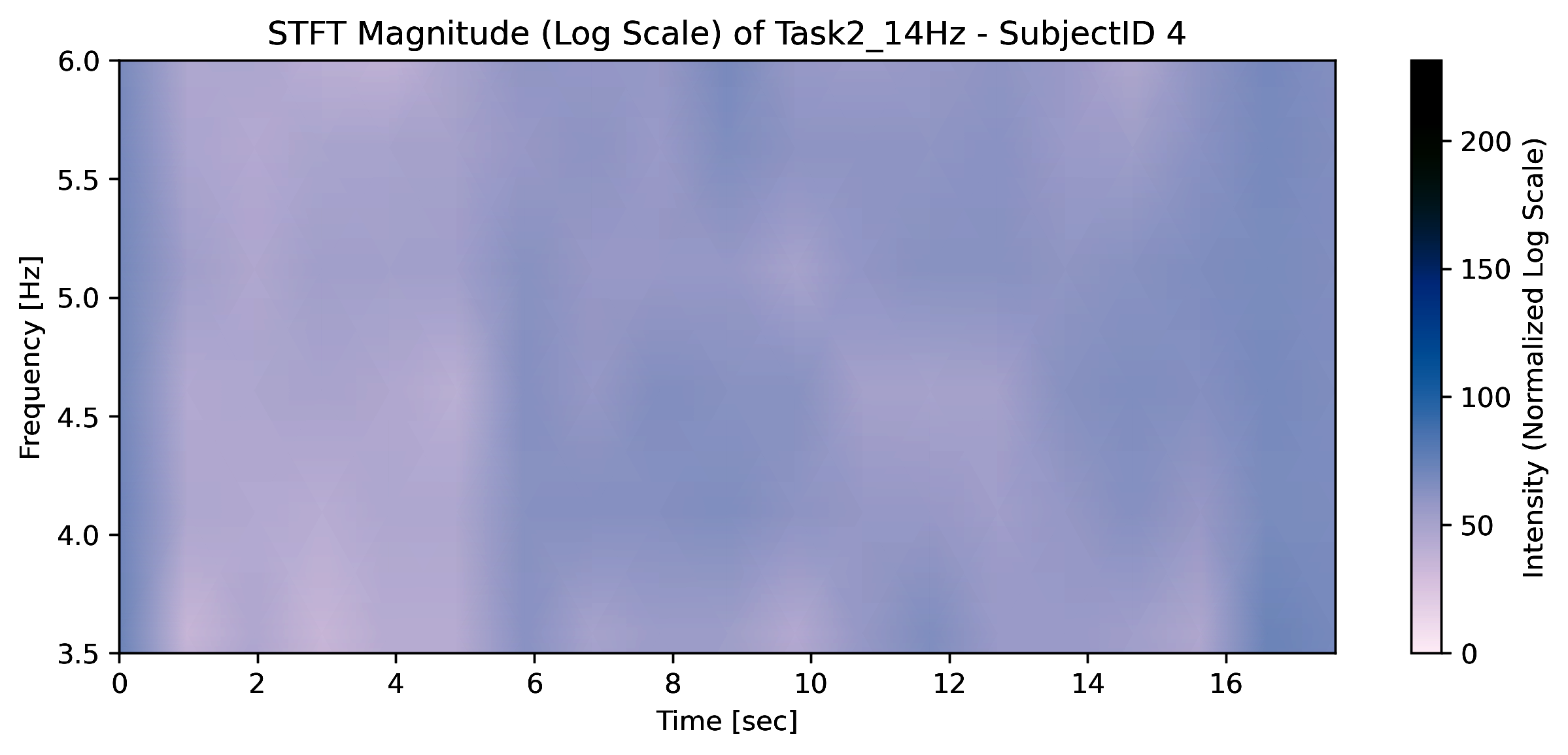

Supplement: Supplementary file 1 [file sensors-26-00157-s001.zip › STFT Images/RFG Images/Task 2 Images/Figure S13 ID 4.png]

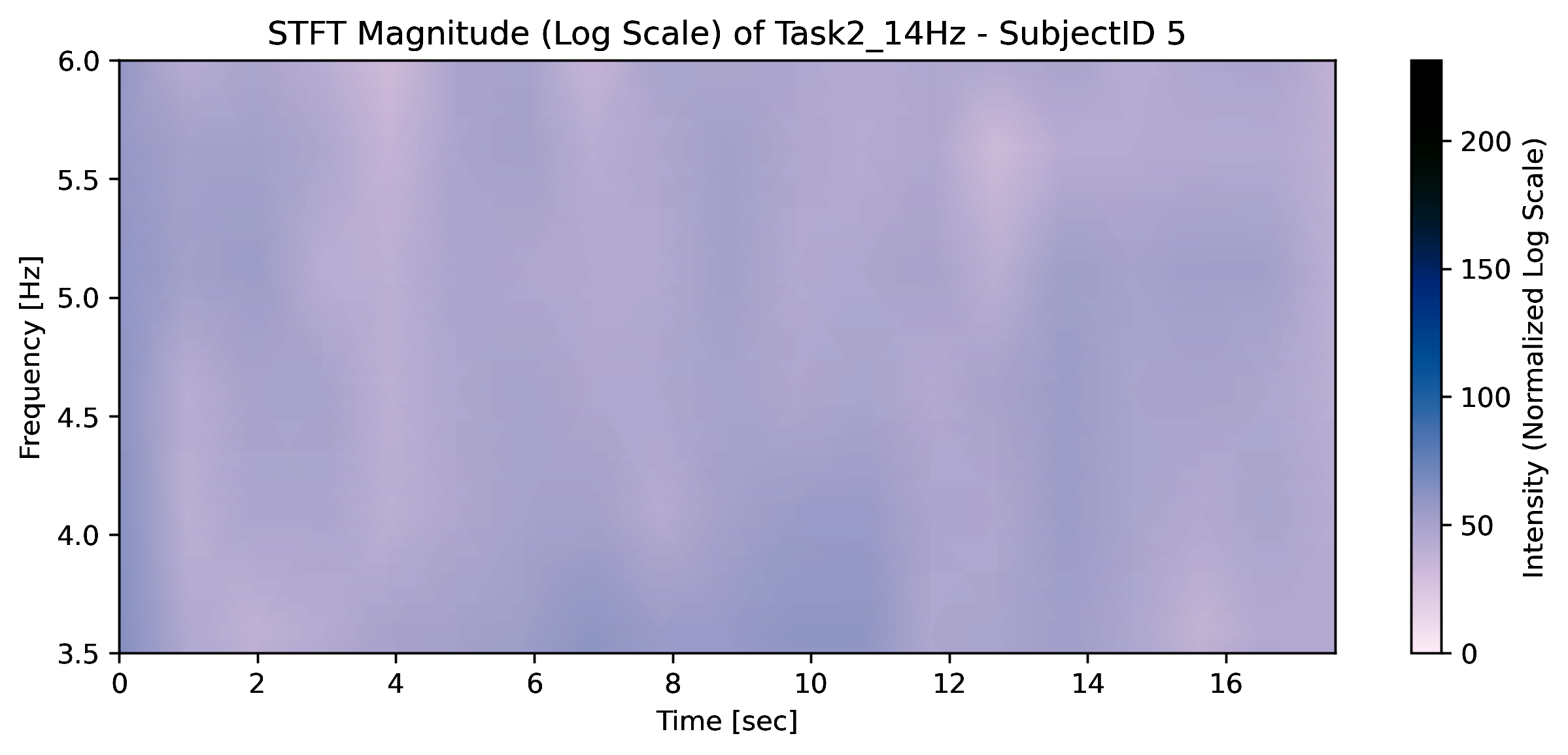

Supplement: Supplementary file 1 [file sensors-26-00157-s001.zip › STFT Images/RFG Images/Task 2 Images/Figure S13 ID 5.png]

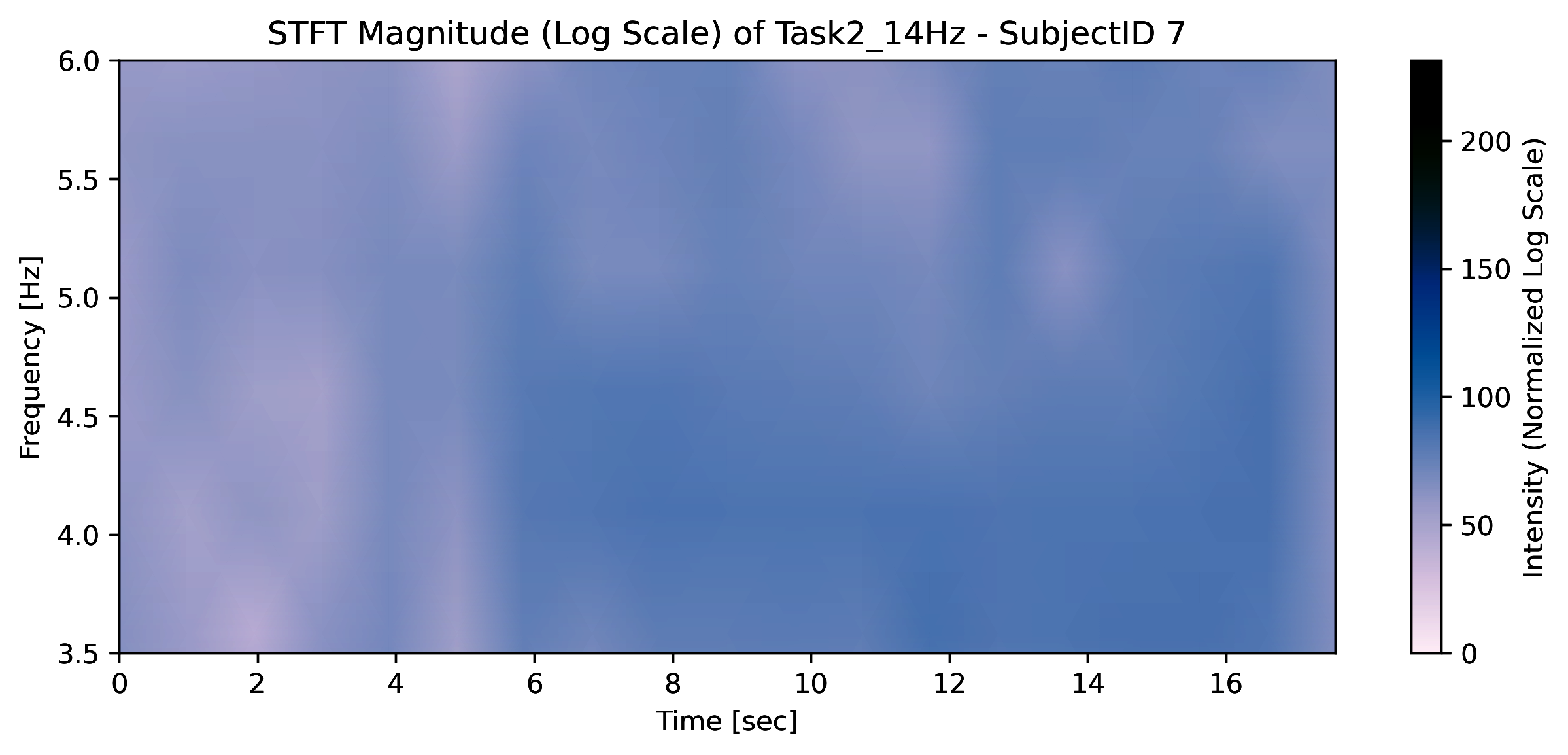

Supplement: Supplementary file 1 [file sensors-26-00157-s001.zip › STFT Images/RFG Images/Task 2 Images/Figure S13 ID 7.png]

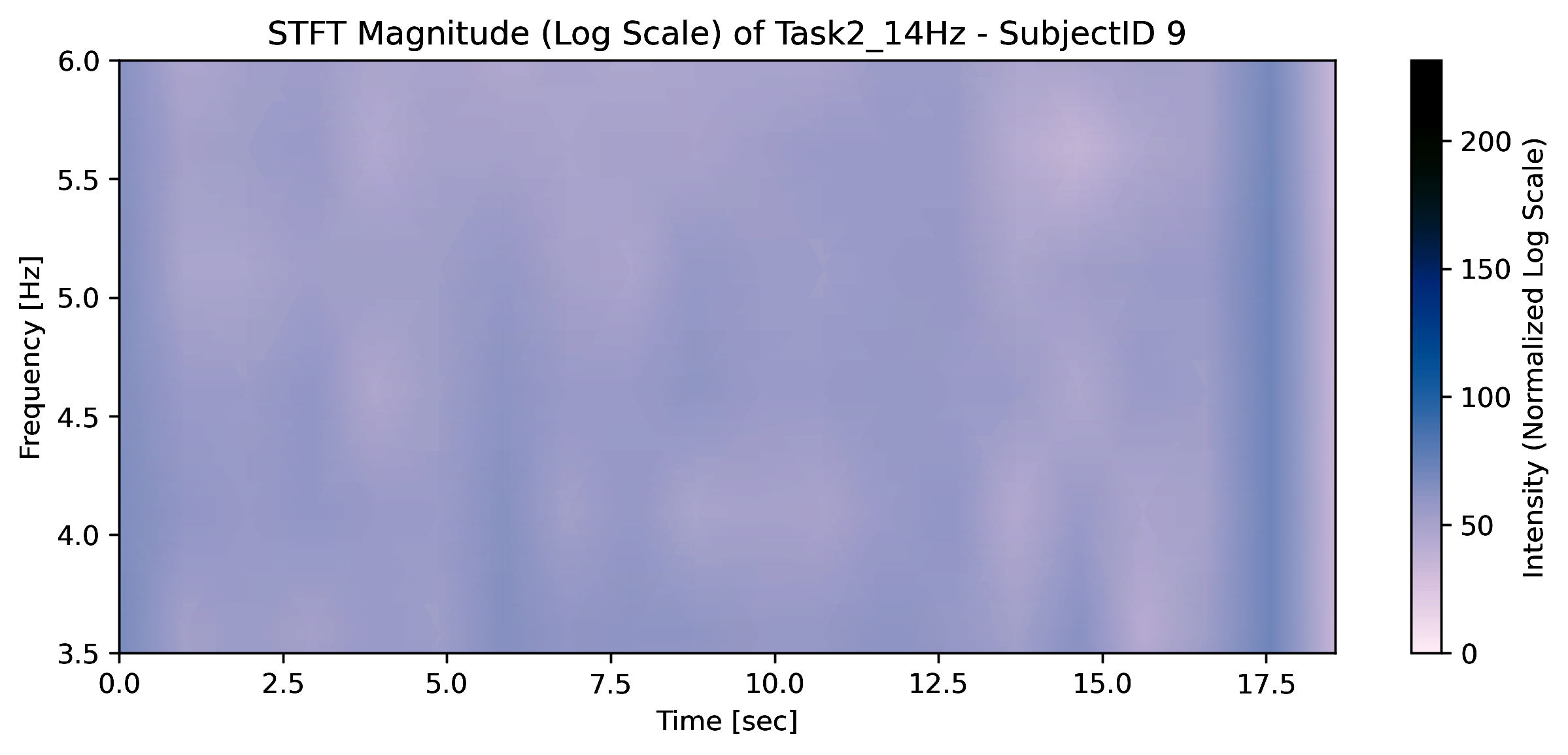

Supplement: Supplementary file 1 [file sensors-26-00157-s001.zip › STFT Images/RFG Images/Task 2 Images/Figure S13 ID 9.png]

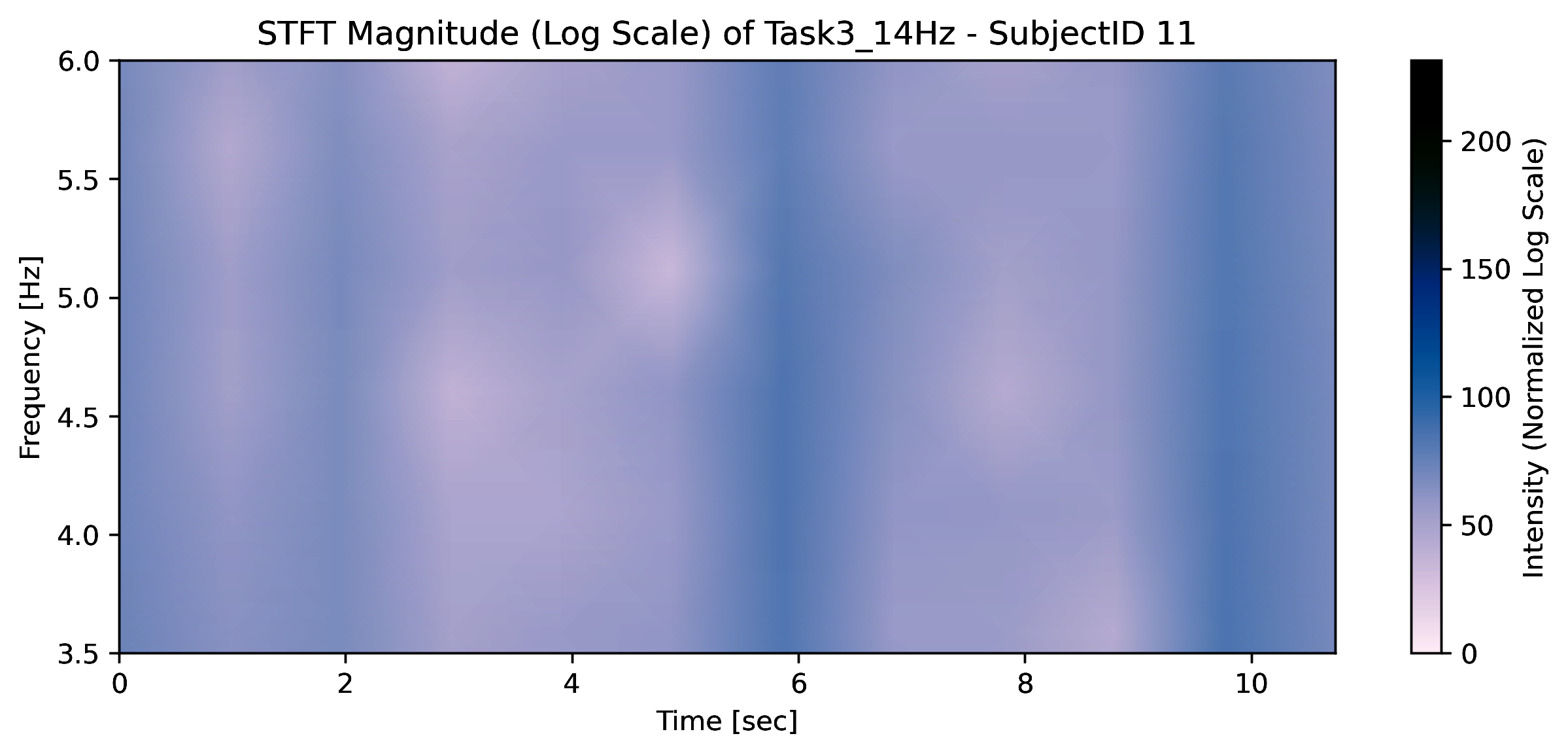

Supplement: Supplementary file 1 [file sensors-26-00157-s001.zip › STFT Images/RFG Images/Task 3 Images/Figure S14 ID 11.png]

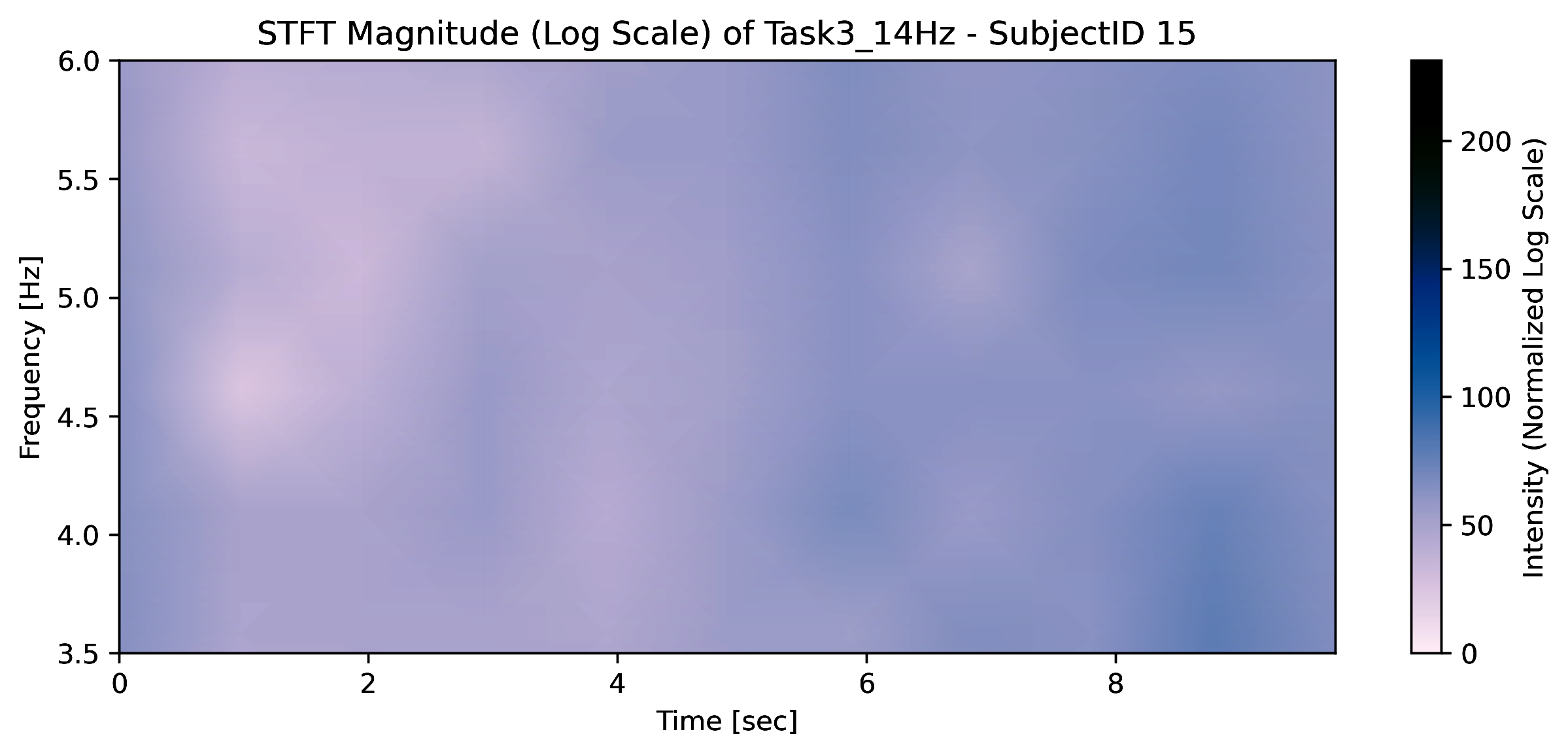

Supplement: Supplementary file 1 [file sensors-26-00157-s001.zip › STFT Images/RFG Images/Task 3 Images/Figure S14 ID 15.png]

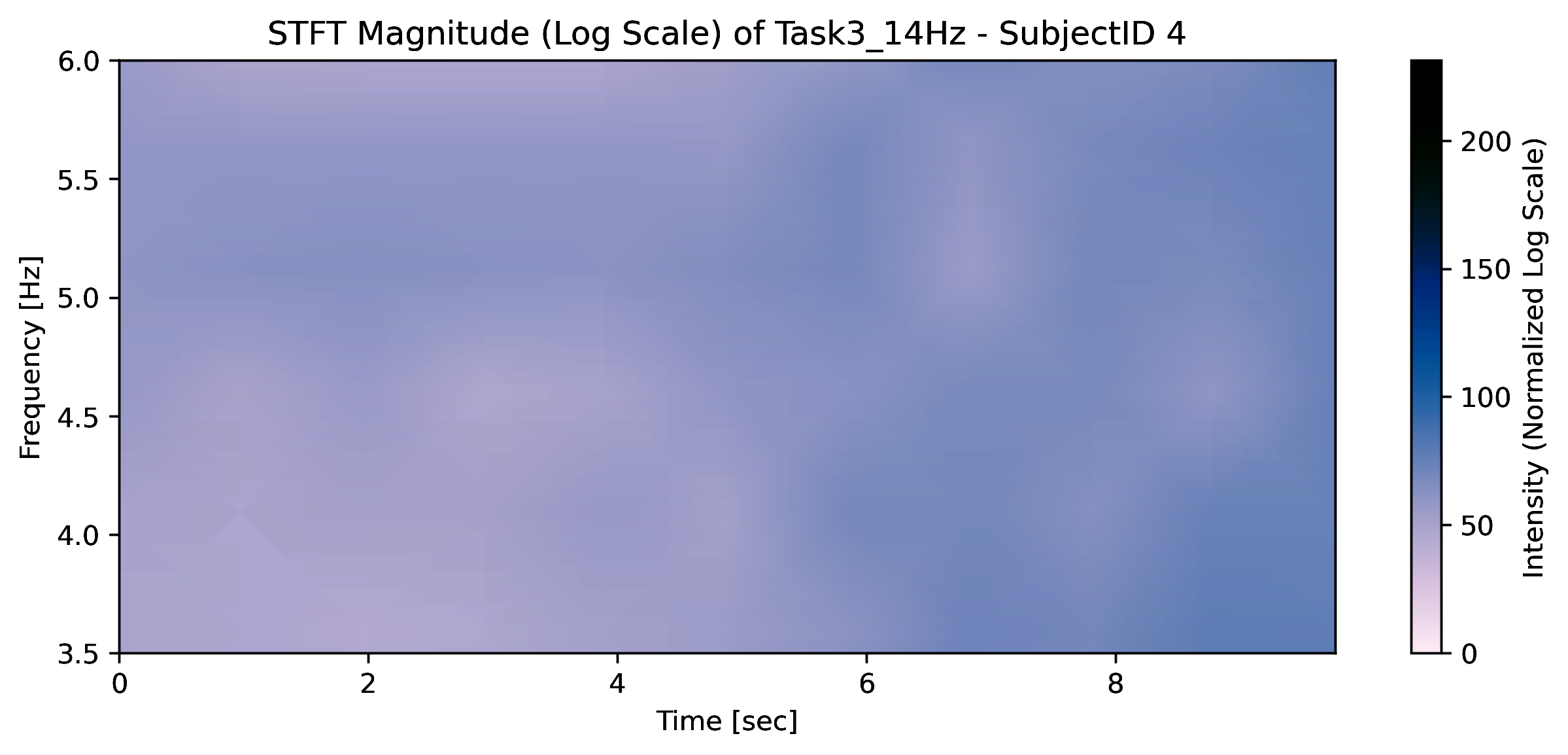

Supplement: Supplementary file 1 [file sensors-26-00157-s001.zip › STFT Images/RFG Images/Task 3 Images/Figure S14 ID 4.png]

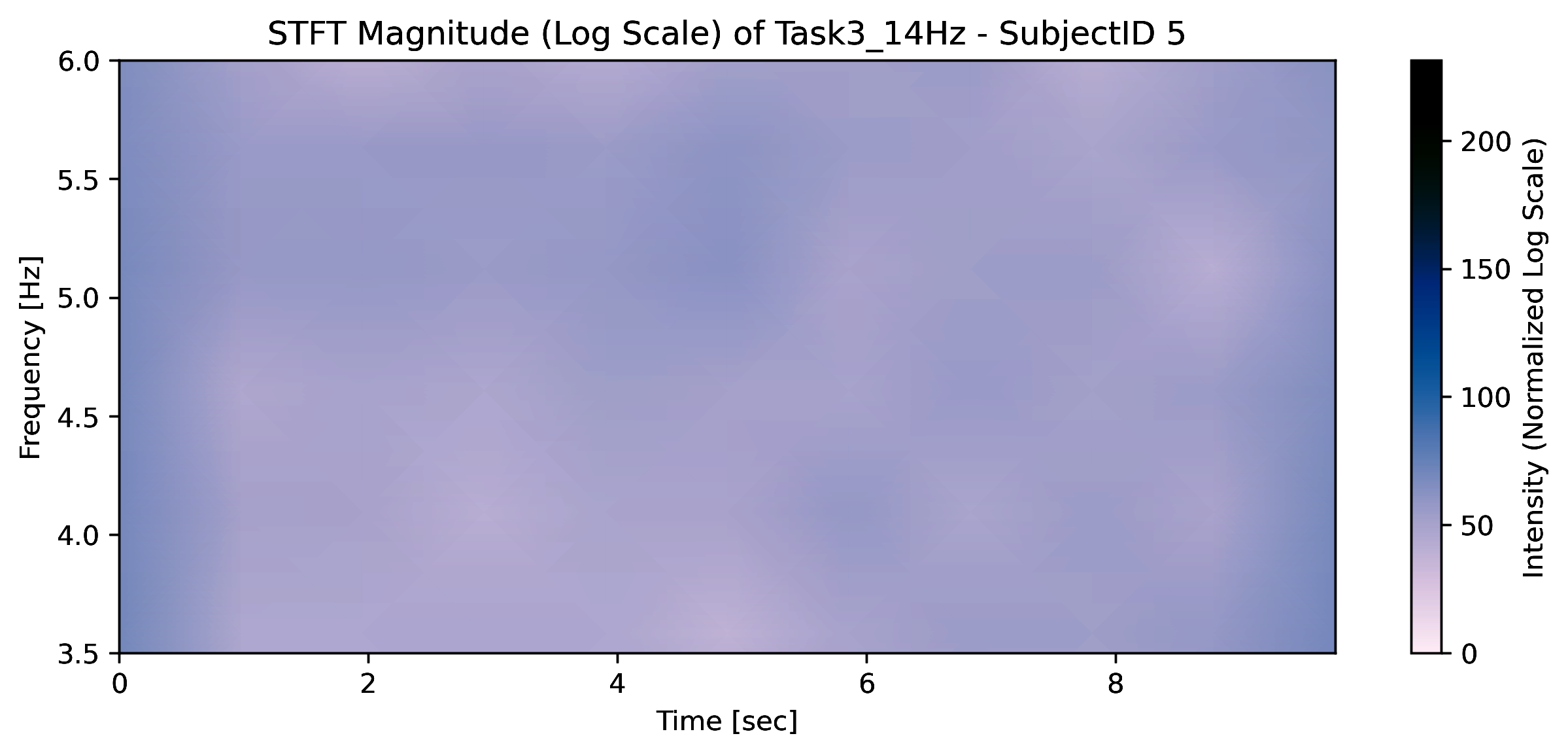

Supplement: Supplementary file 1 [file sensors-26-00157-s001.zip › STFT Images/RFG Images/Task 3 Images/Figure S14 ID 5.png]

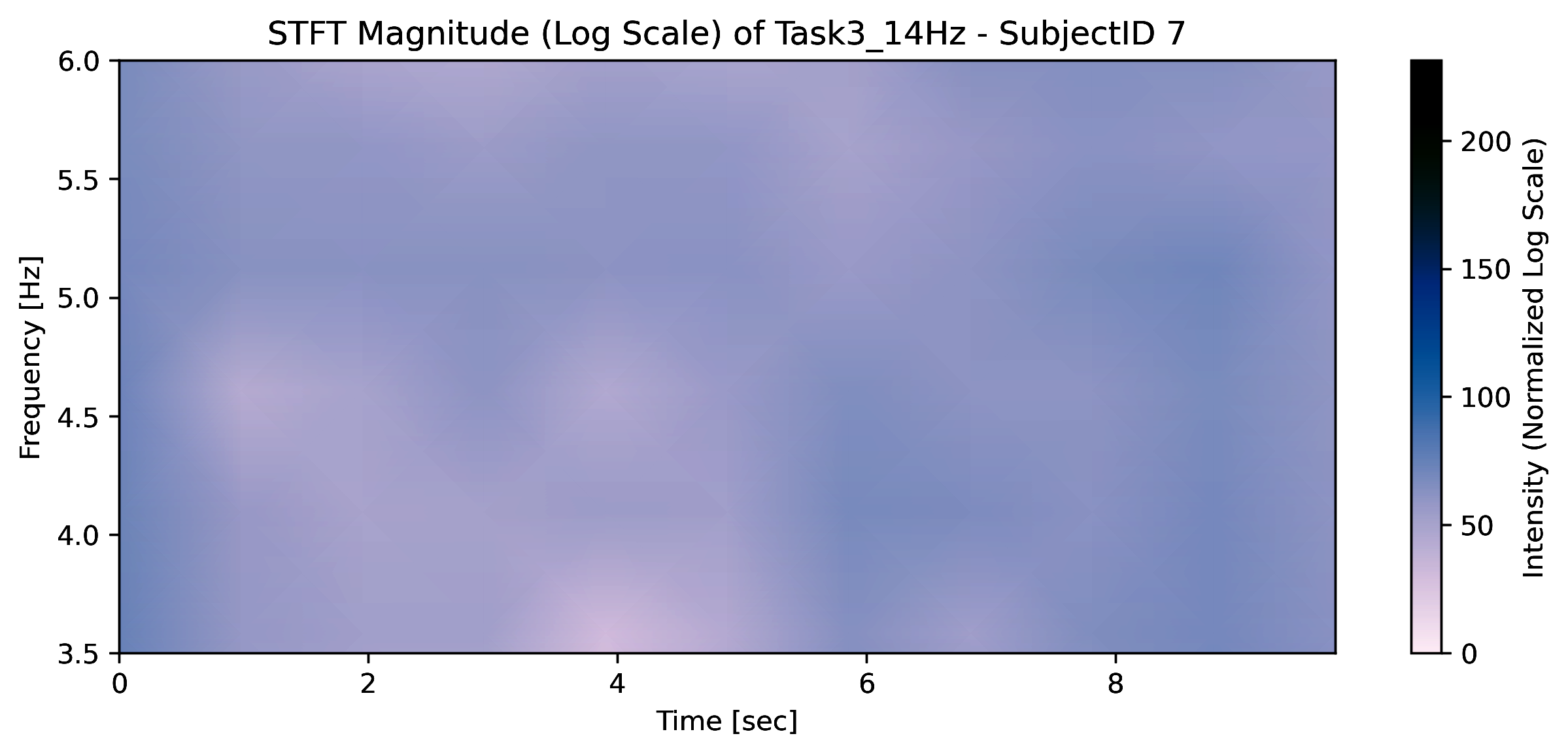

Supplement: Supplementary file 1 [file sensors-26-00157-s001.zip › STFT Images/RFG Images/Task 3 Images/Figure S14 ID 7.png]

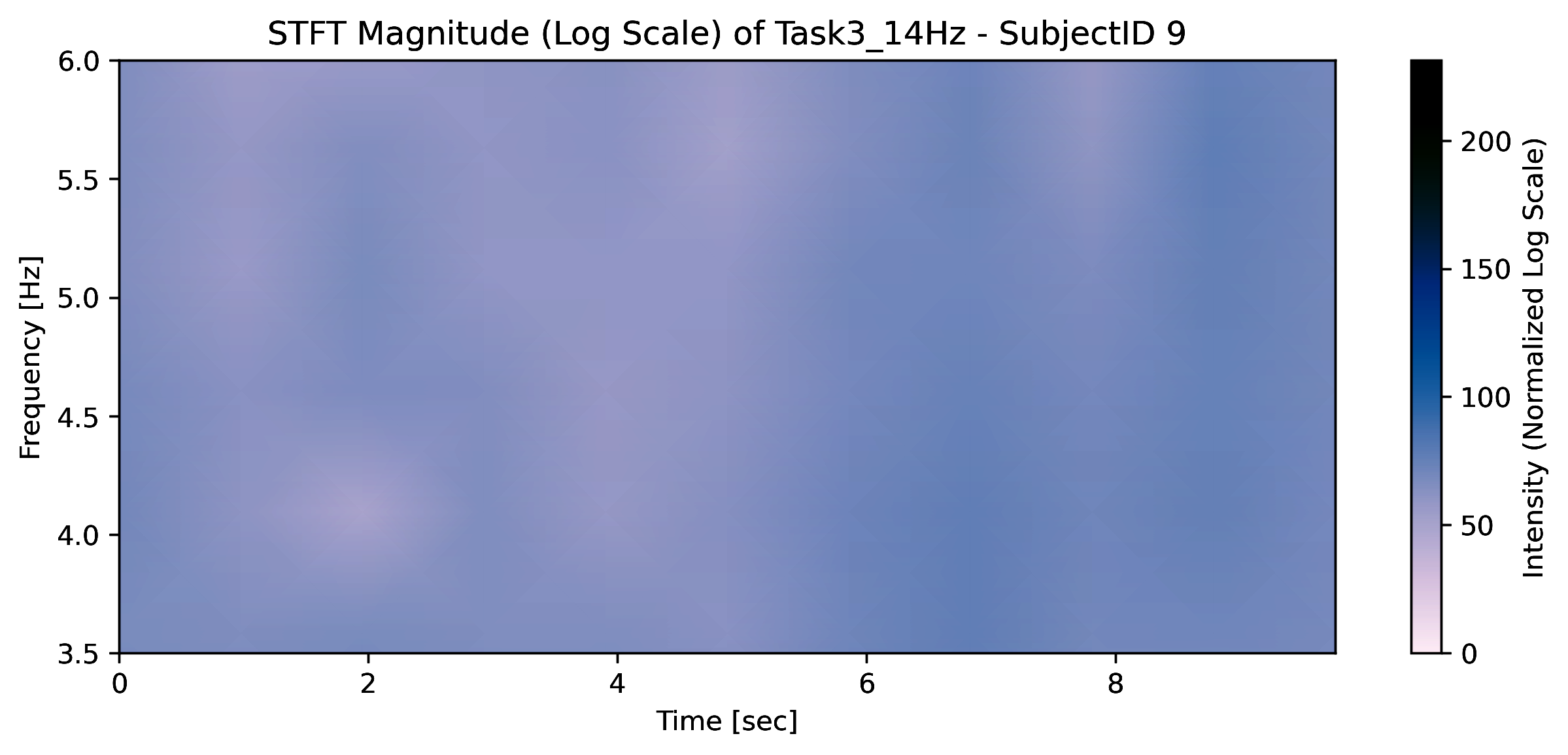

Supplement: Supplementary file 1 [file sensors-26-00157-s001.zip › STFT Images/RFG Images/Task 3 Images/Figure S14 ID 9.png]

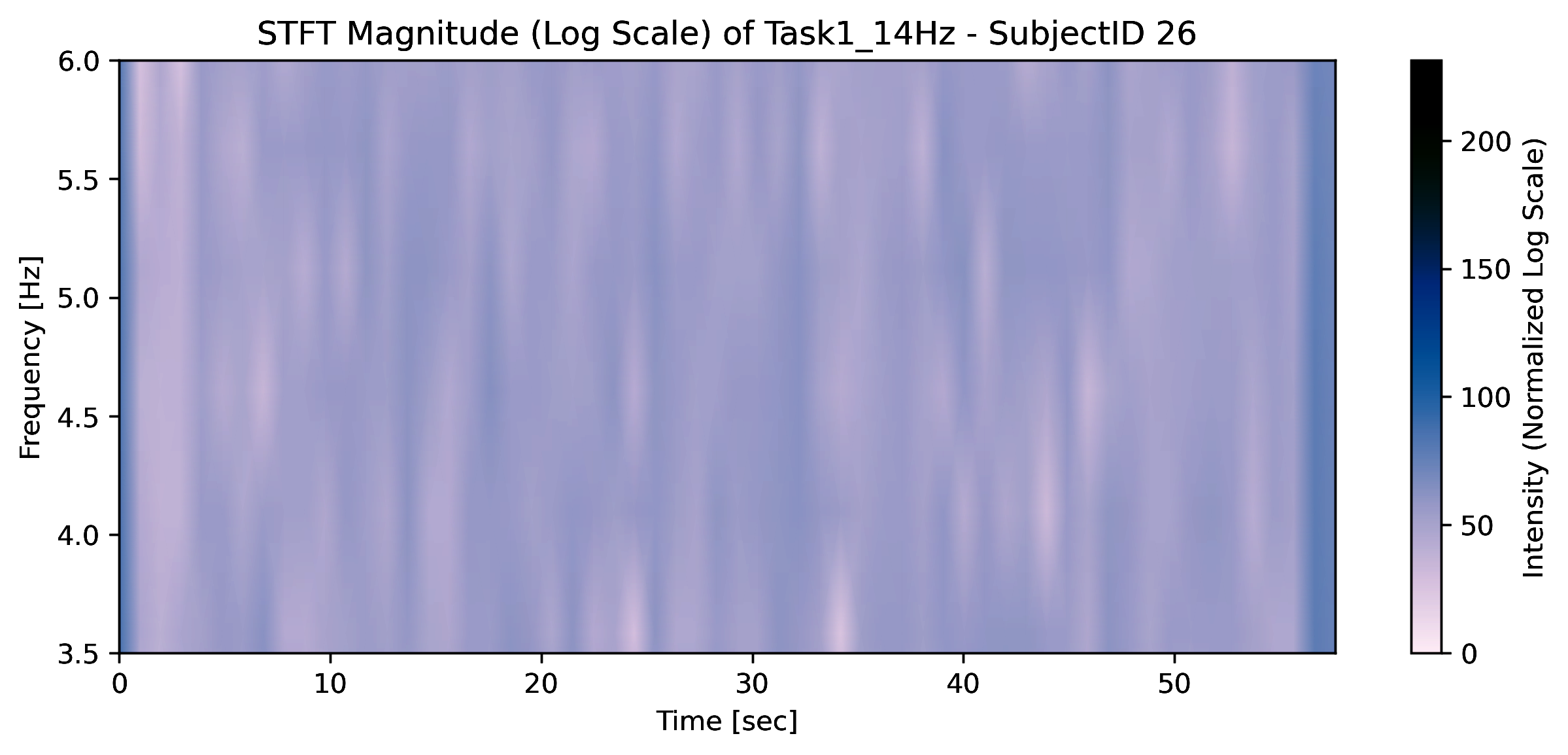

Supplement: Supplementary file 1 [file sensors-26-00157-s001.zip › STFT Images/RFG Images/Tasks 1-7 Images/Figure S15 Task 1 ID 26.png]

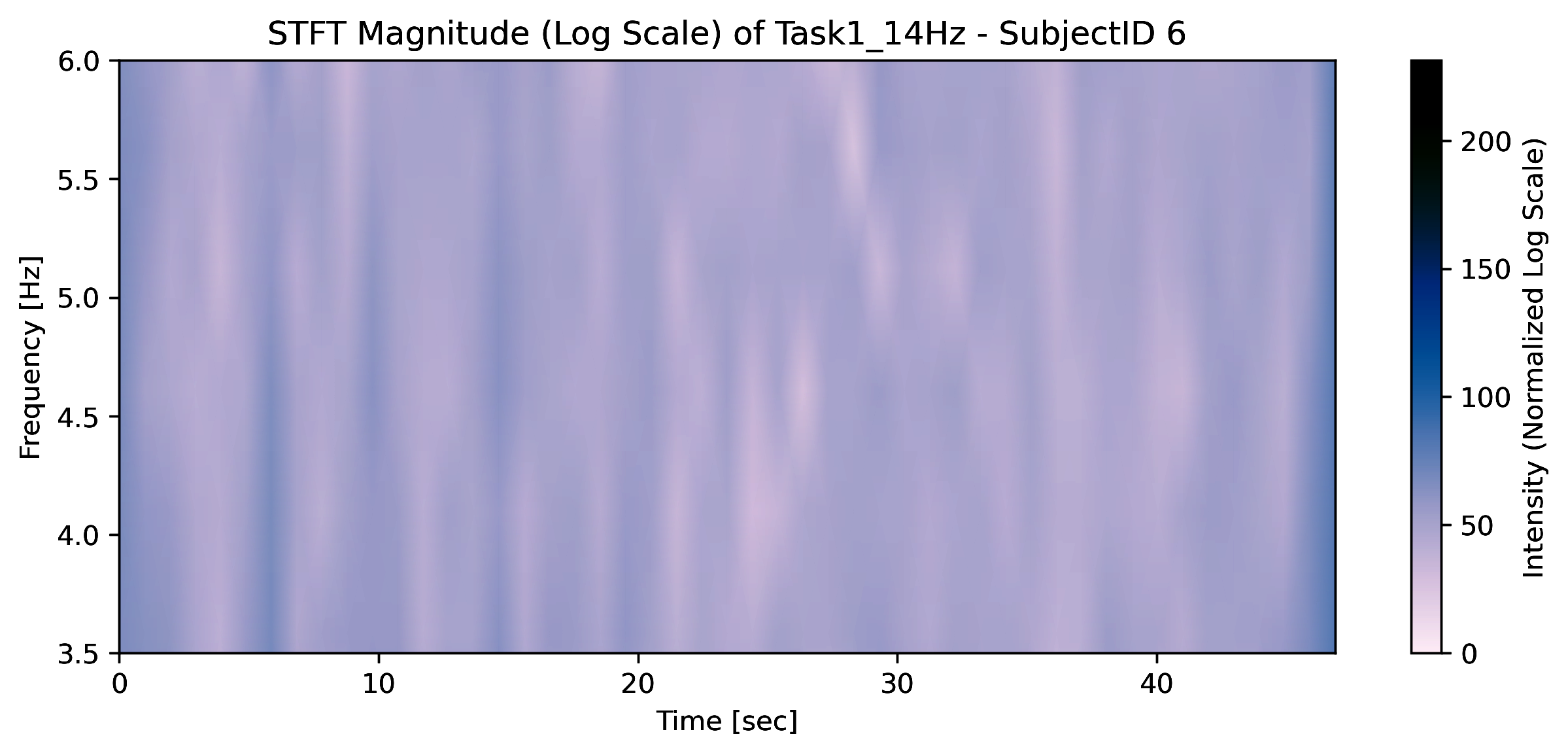

Supplement: Supplementary file 1 [file sensors-26-00157-s001.zip › STFT Images/RFG Images/Tasks 1-7 Images/Figure S15 Task 1 ID 6.png]

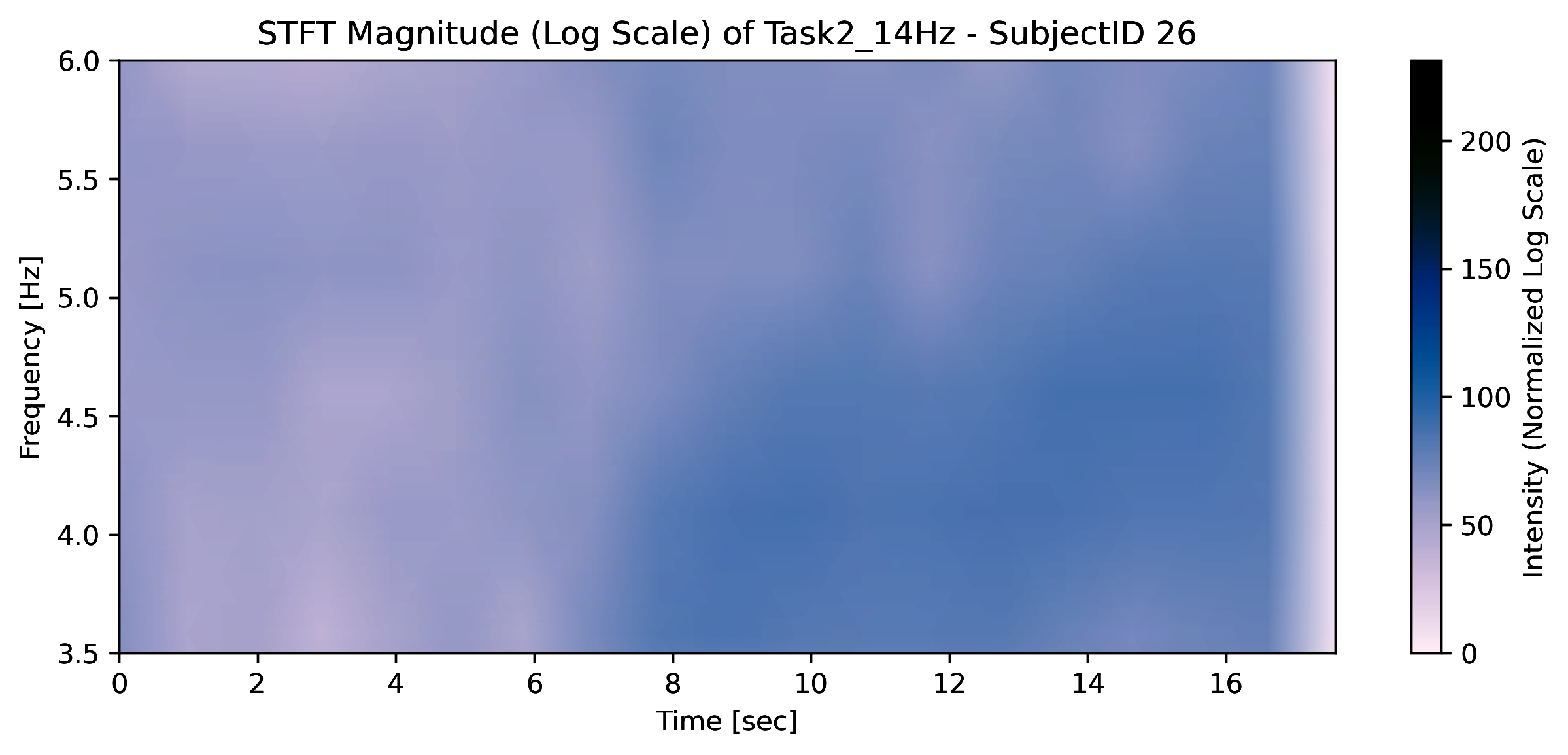

Supplement: Supplementary file 1 [file sensors-26-00157-s001.zip › STFT Images/RFG Images/Tasks 1-7 Images/Figure S15 Task 2 ID 26.png]

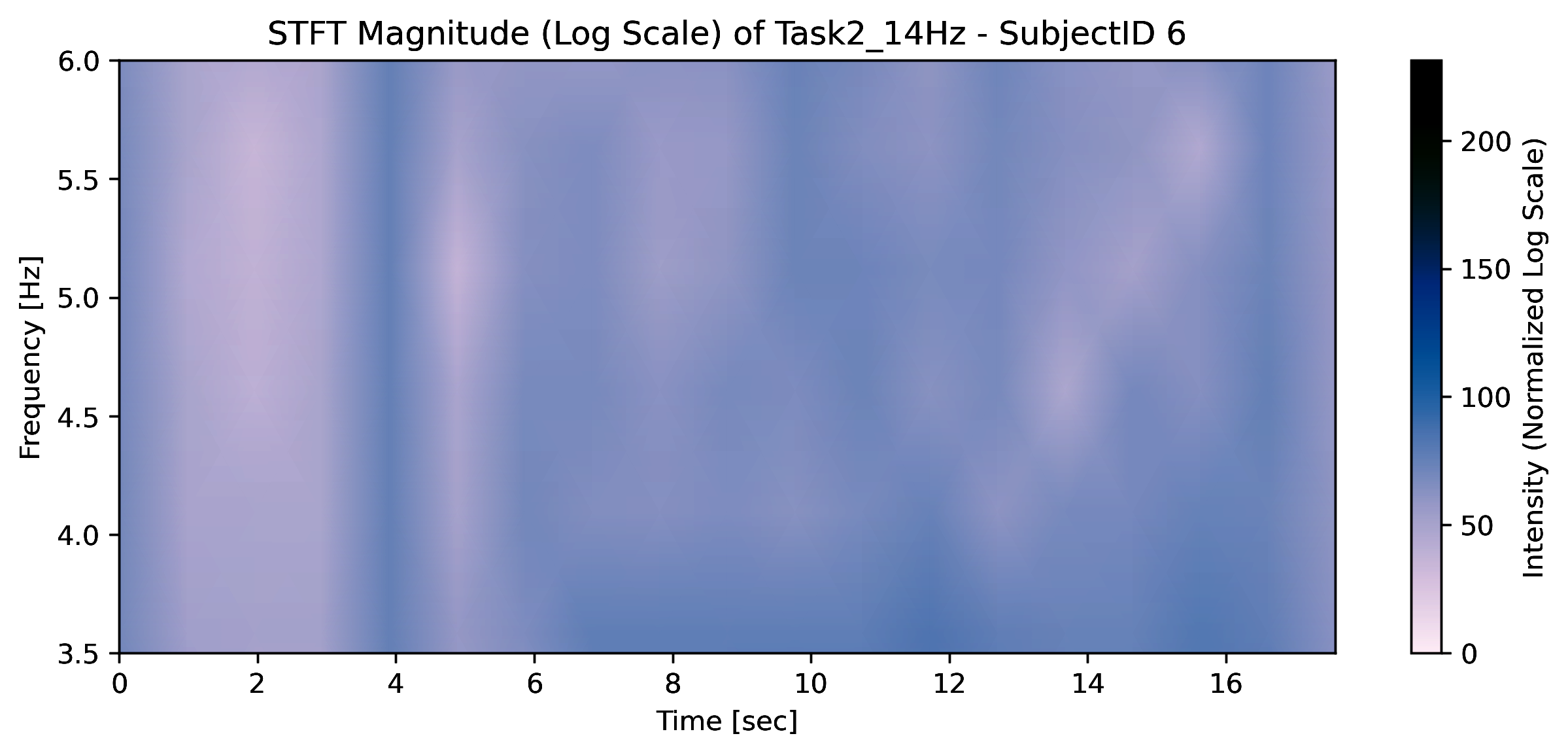

Supplement: Supplementary file 1 [file sensors-26-00157-s001.zip › STFT Images/RFG Images/Tasks 1-7 Images/Figure S15 Task 2 ID 6.png]

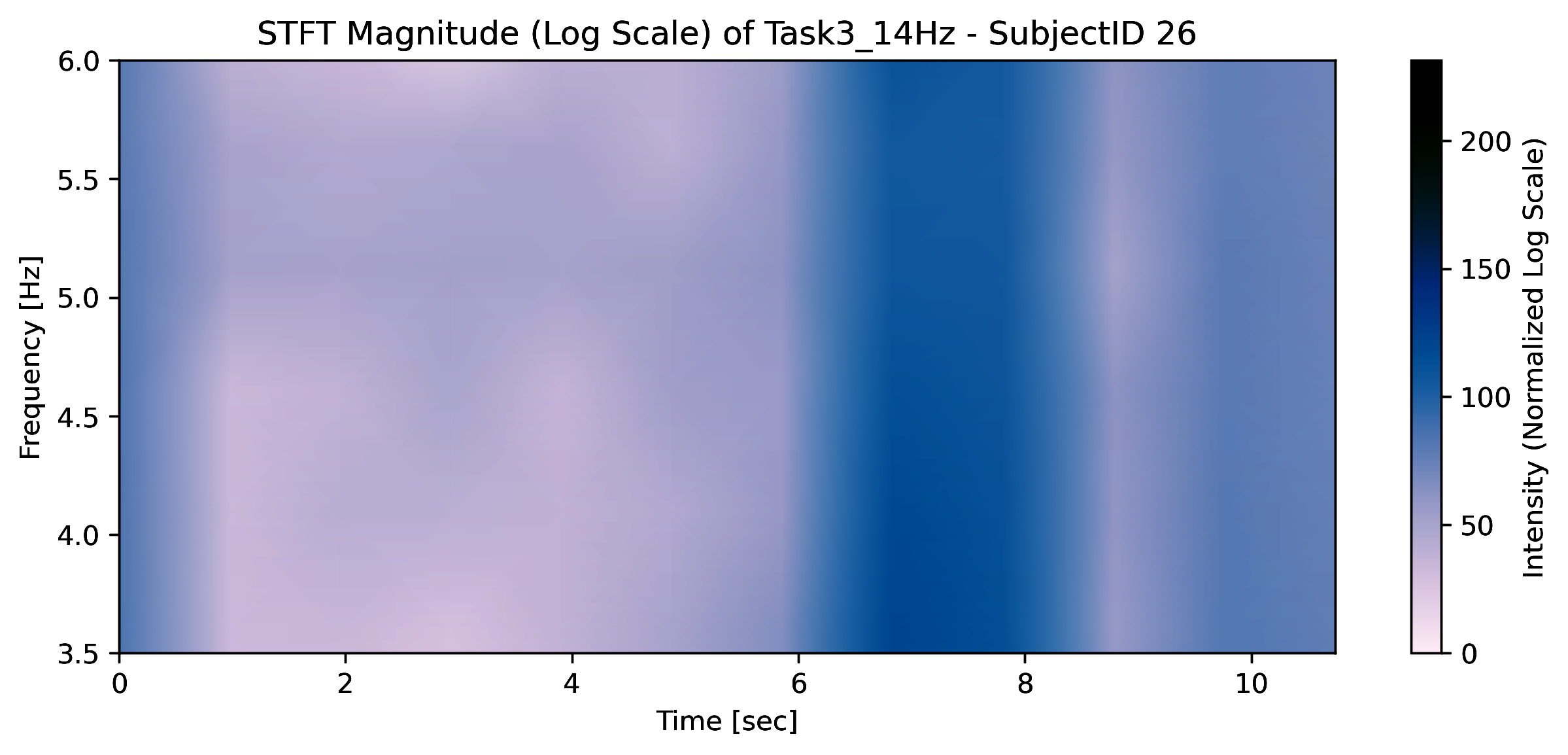

Supplement: Supplementary file 1 [file sensors-26-00157-s001.zip › STFT Images/RFG Images/Tasks 1-7 Images/Figure S15 Task 3 ID 26.png]

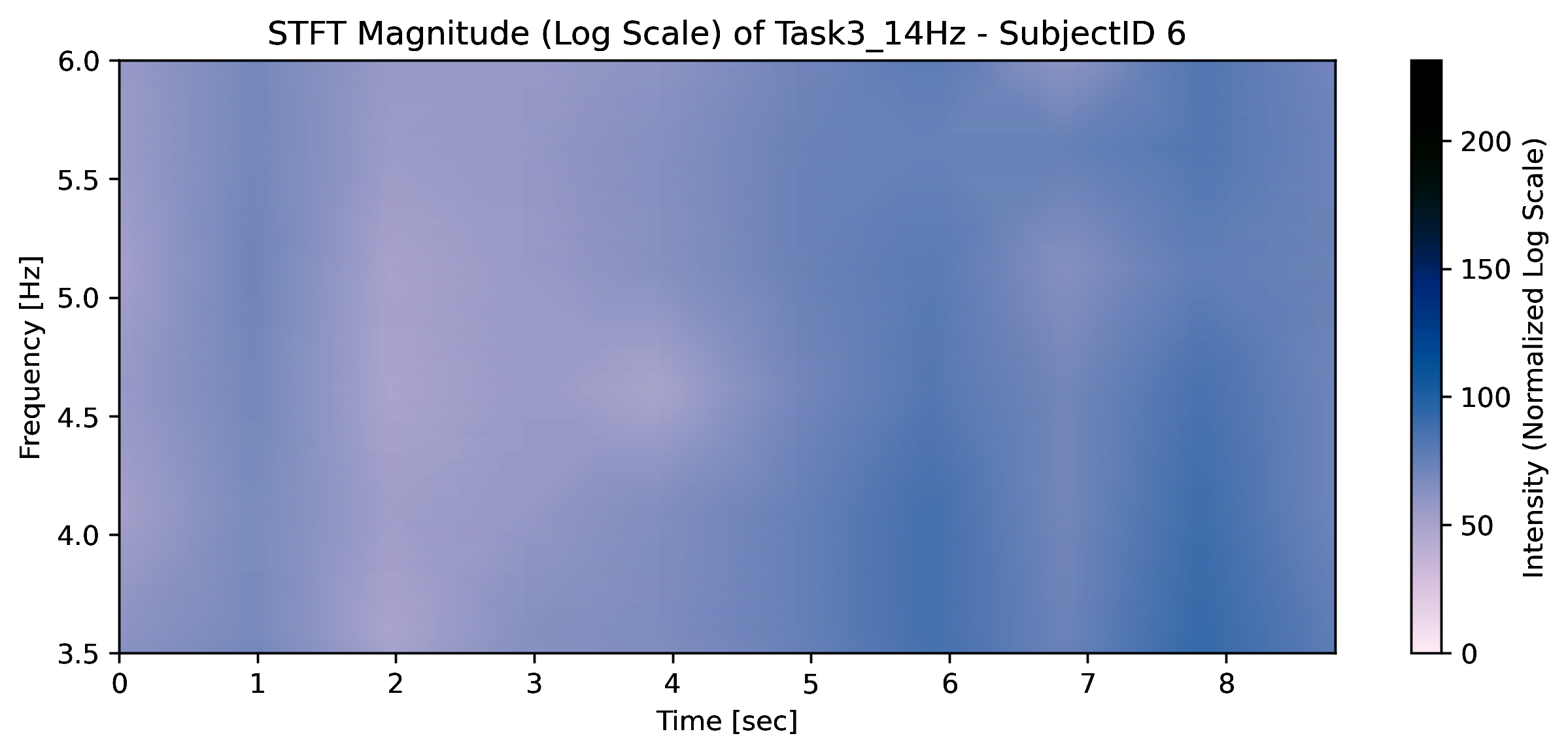

Supplement: Supplementary file 1 [file sensors-26-00157-s001.zip › STFT Images/RFG Images/Tasks 1-7 Images/Figure S15 Task 3 ID 6.png]

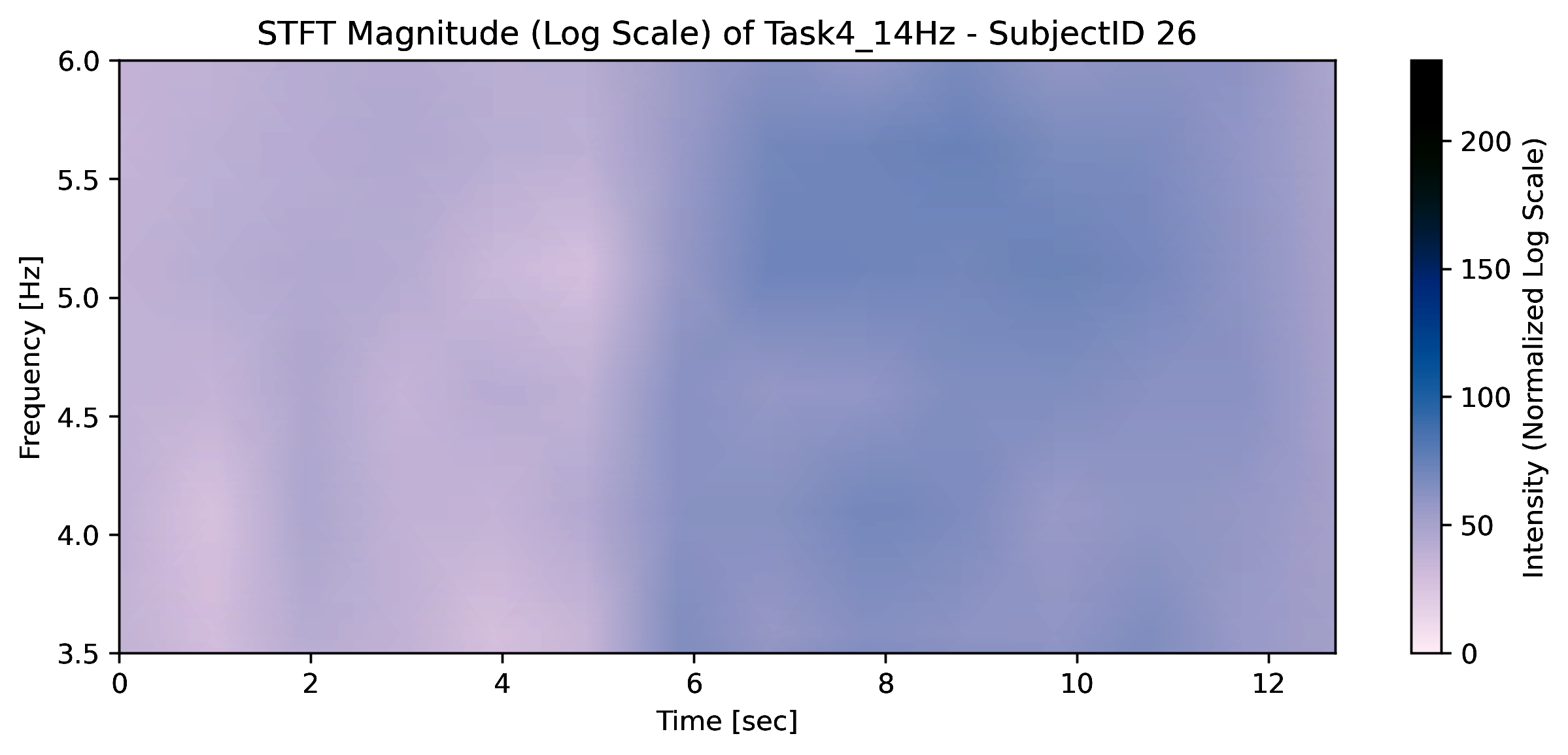

Supplement: Supplementary file 1 [file sensors-26-00157-s001.zip › STFT Images/RFG Images/Tasks 1-7 Images/Figure S15 Task 4 ID 26.png]

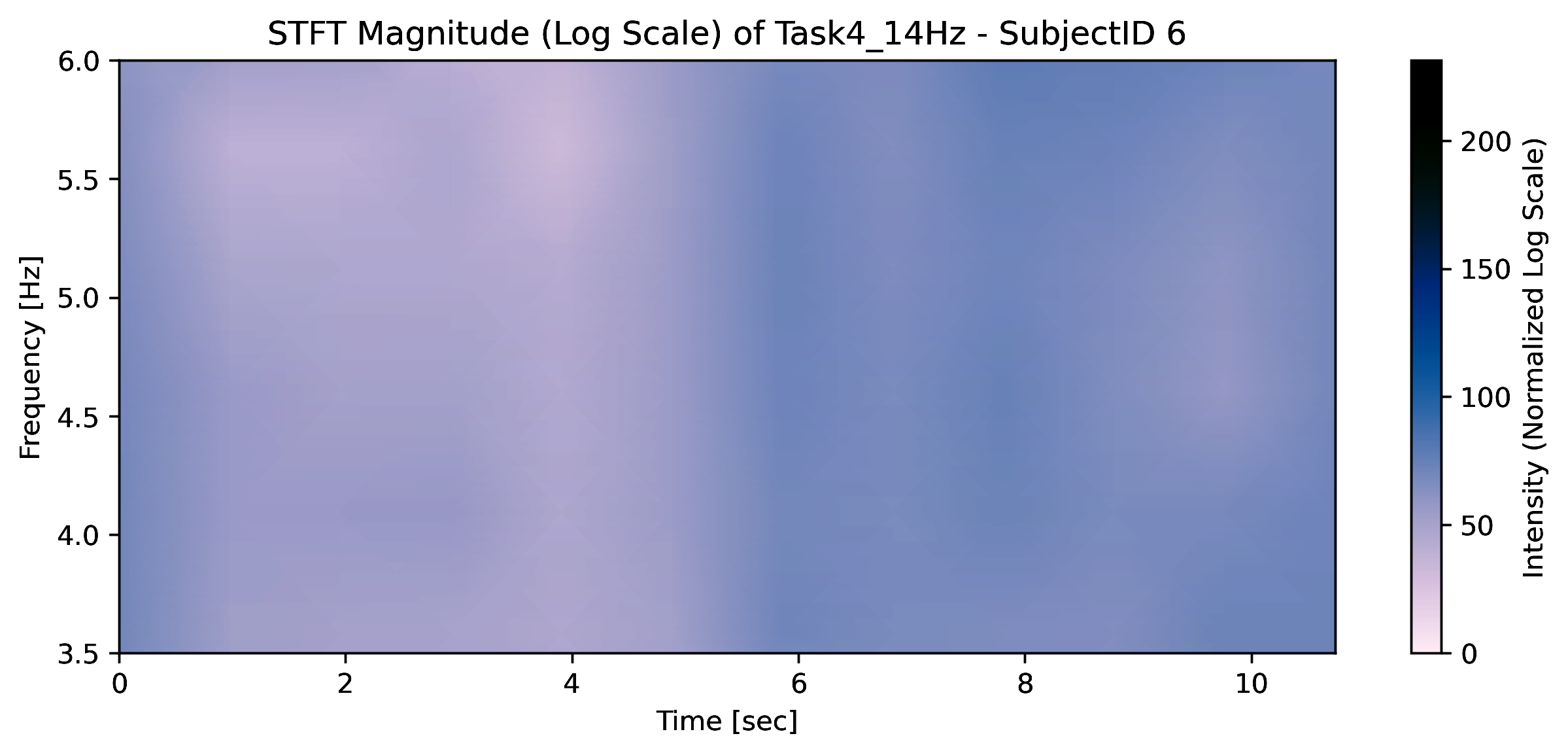

Supplement: Supplementary file 1 [file sensors-26-00157-s001.zip › STFT Images/RFG Images/Tasks 1-7 Images/Figure S15 Task 4 ID 6.png]

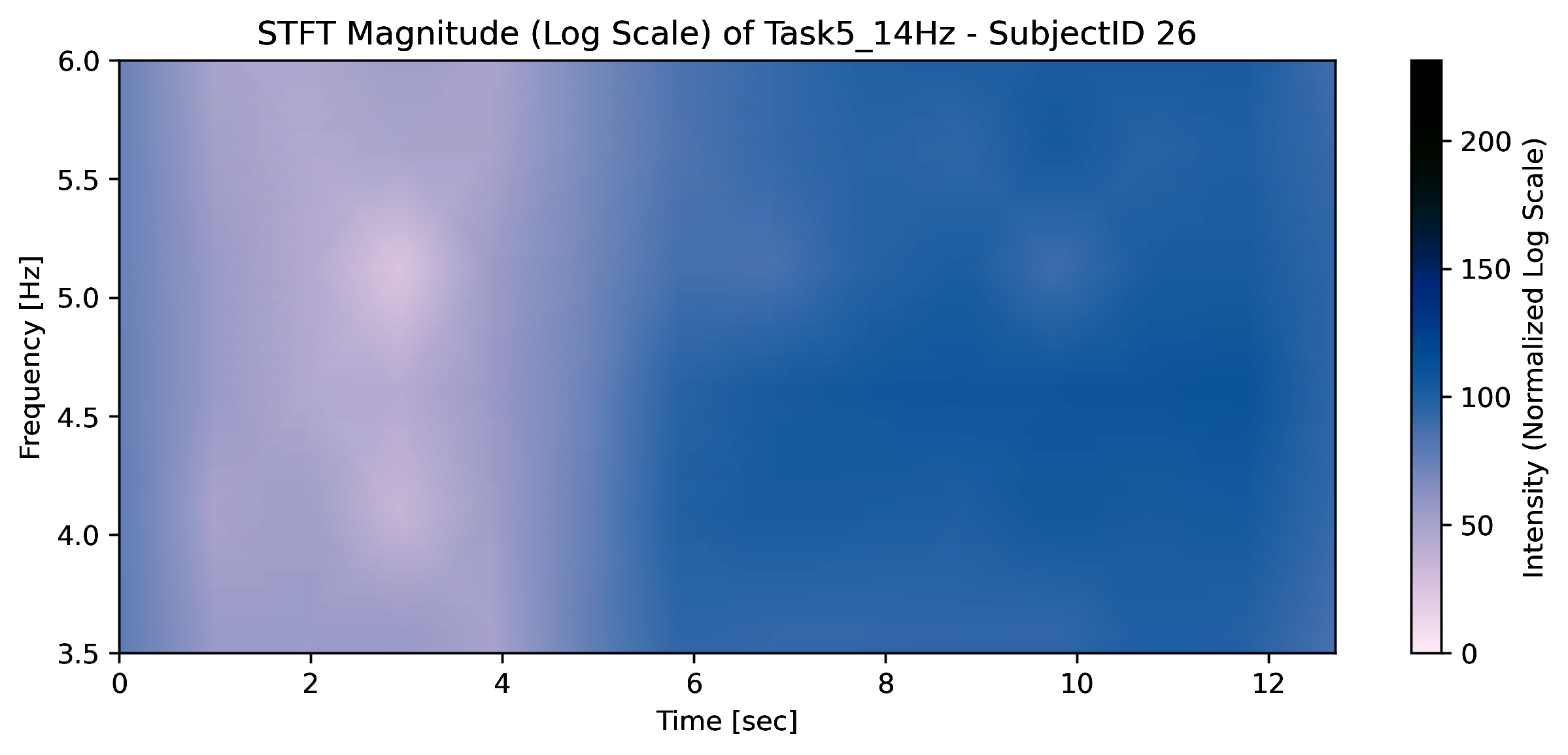

Supplement: Supplementary file 1 [file sensors-26-00157-s001.zip › STFT Images/RFG Images/Tasks 1-7 Images/Figure S15 Task 5 ID 26.png]

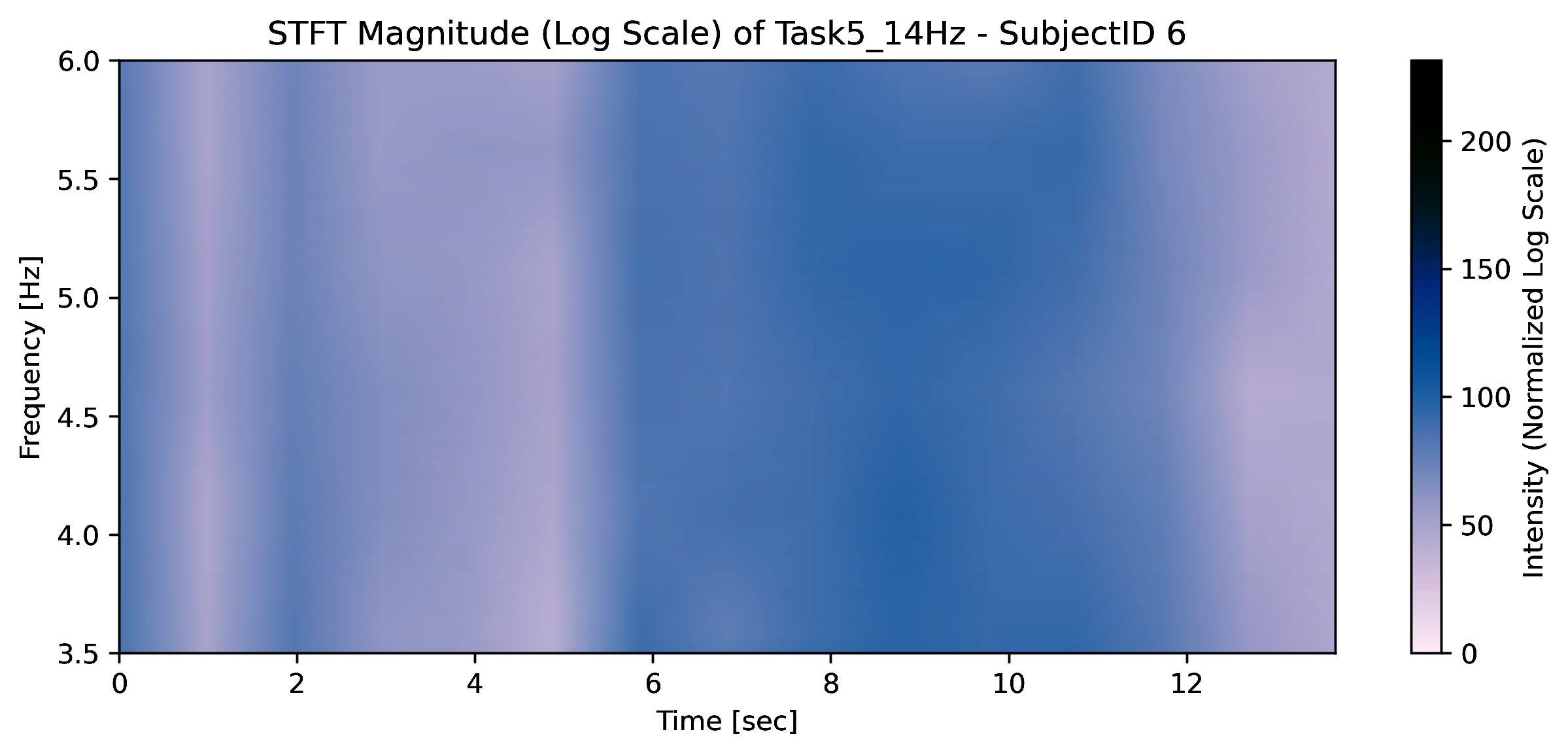

Supplement: Supplementary file 1 [file sensors-26-00157-s001.zip › STFT Images/RFG Images/Tasks 1-7 Images/Figure S15 Task 5 ID 6.png]

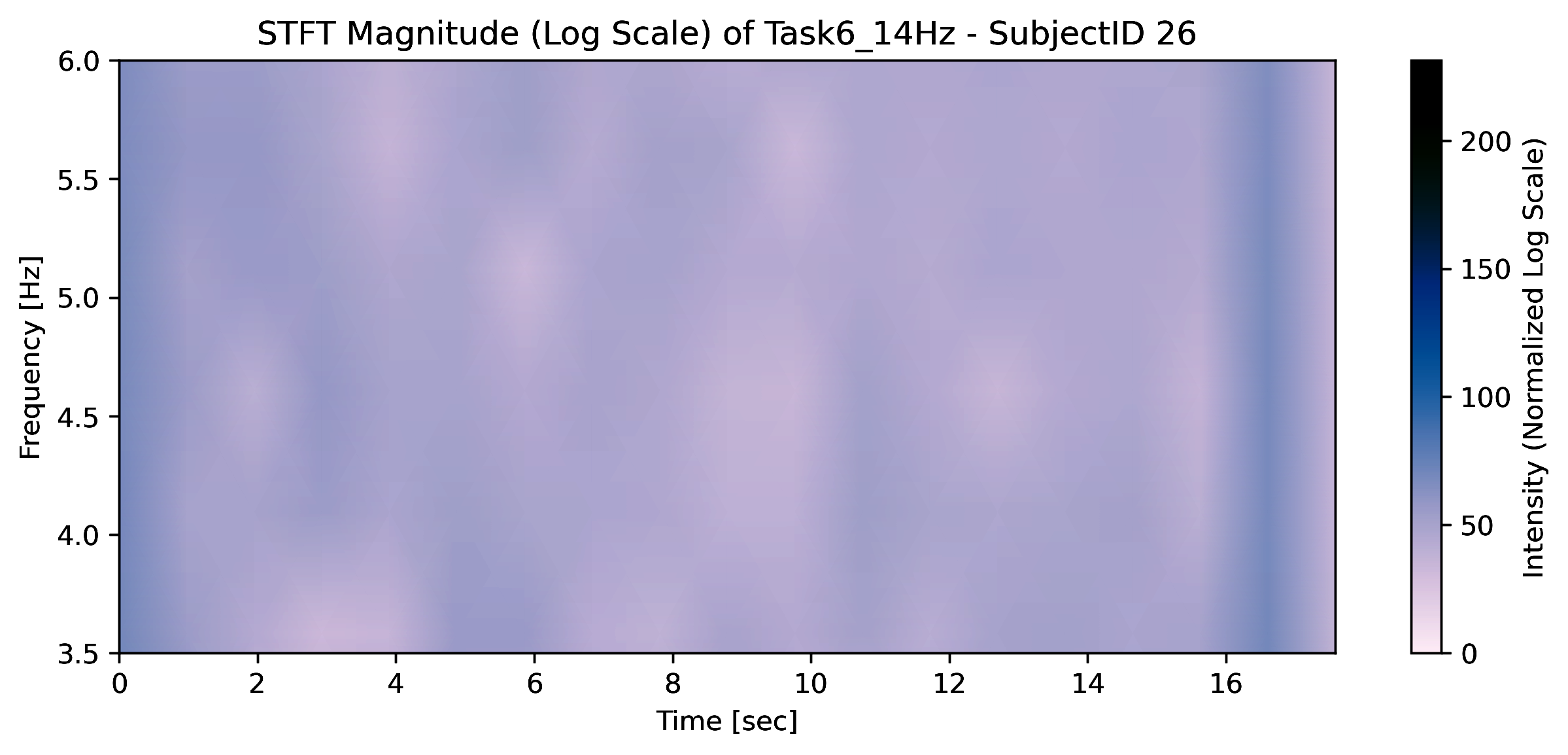

Supplement: Supplementary file 1 [file sensors-26-00157-s001.zip › STFT Images/RFG Images/Tasks 1-7 Images/Figure S15 Task 6 ID 26.png]

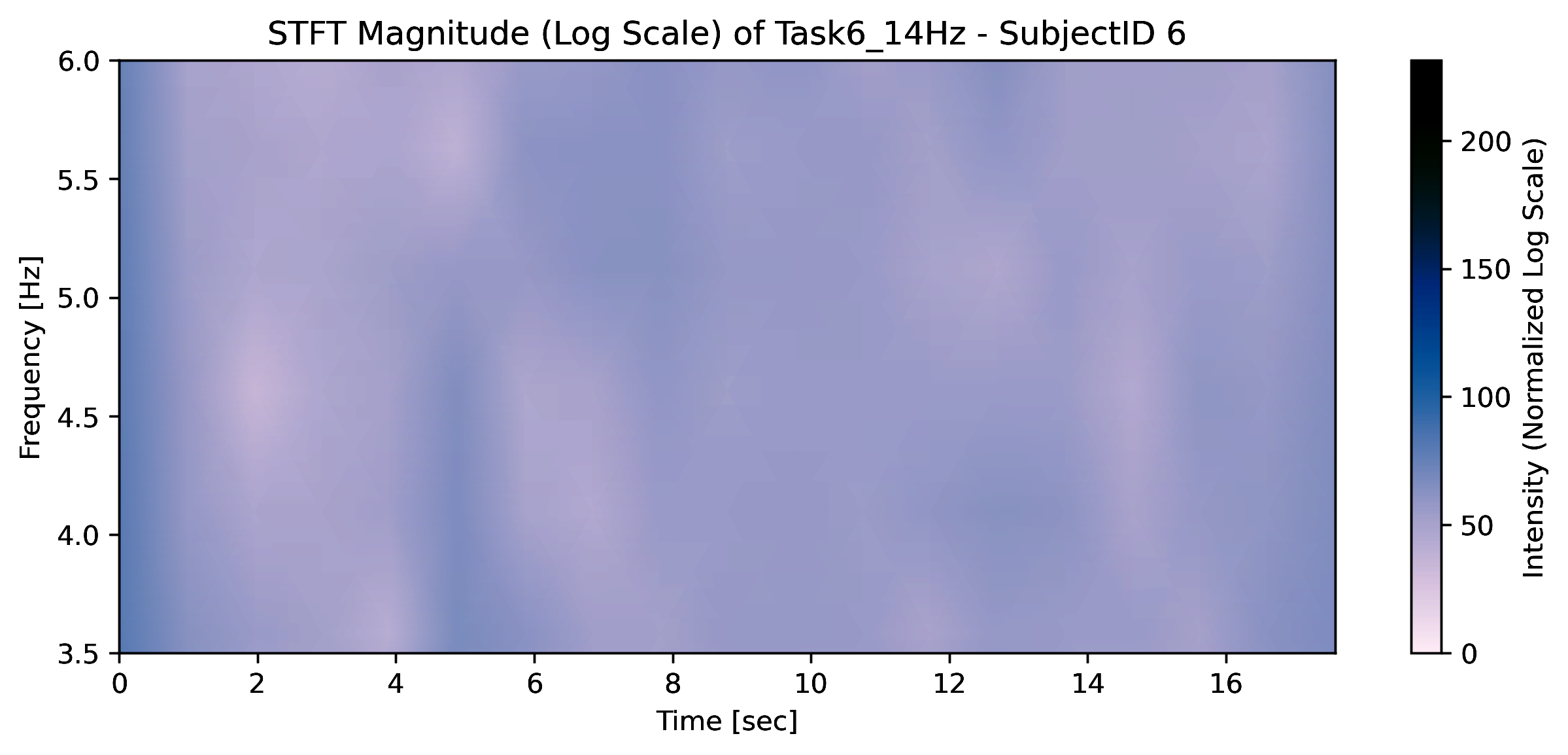

Supplement: Supplementary file 1 [file sensors-26-00157-s001.zip › STFT Images/RFG Images/Tasks 1-7 Images/Figure S15 Task 6 ID 6.png]

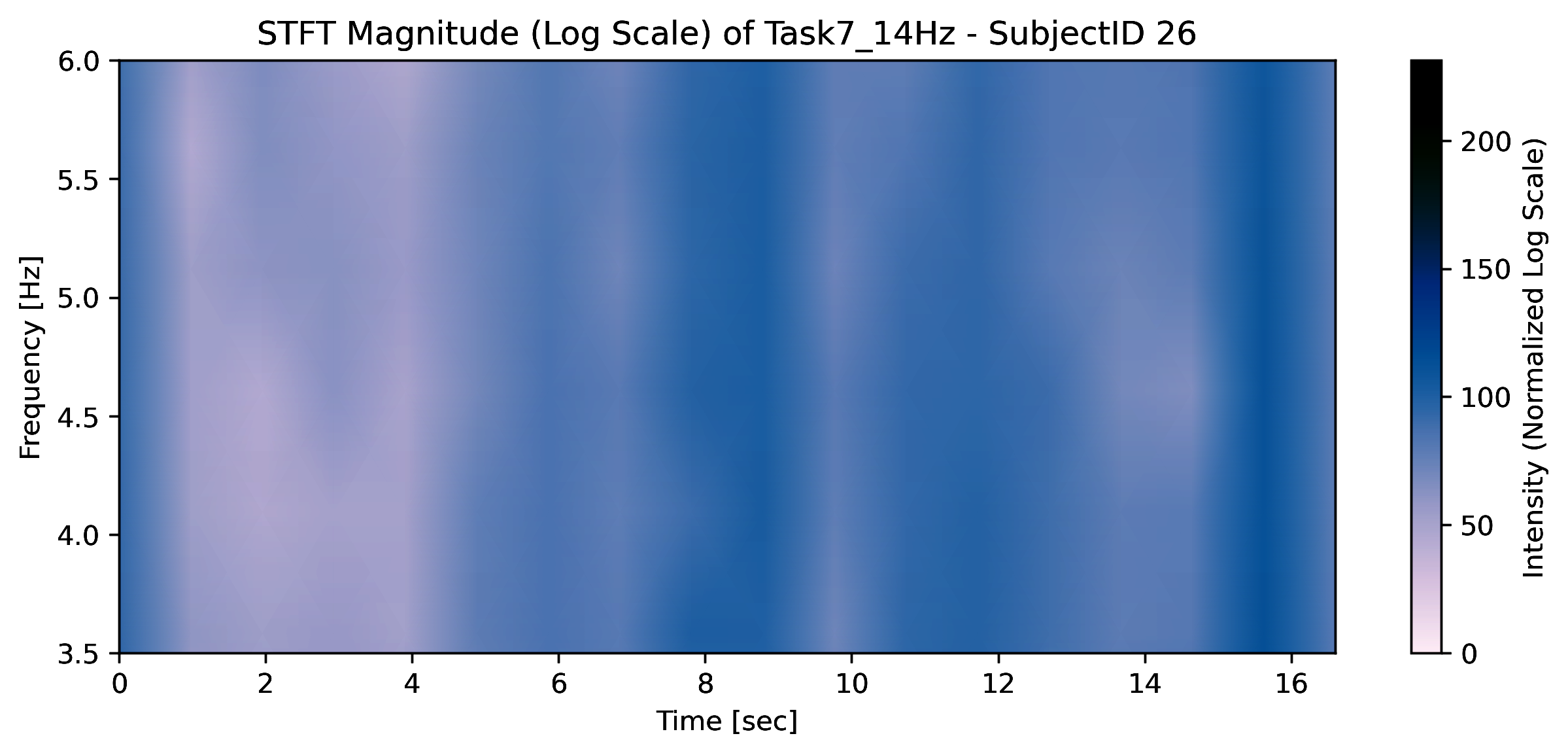

Supplement: Supplementary file 1 [file sensors-26-00157-s001.zip › STFT Images/RFG Images/Tasks 1-7 Images/Figure S15 Task 7 ID 26.png]

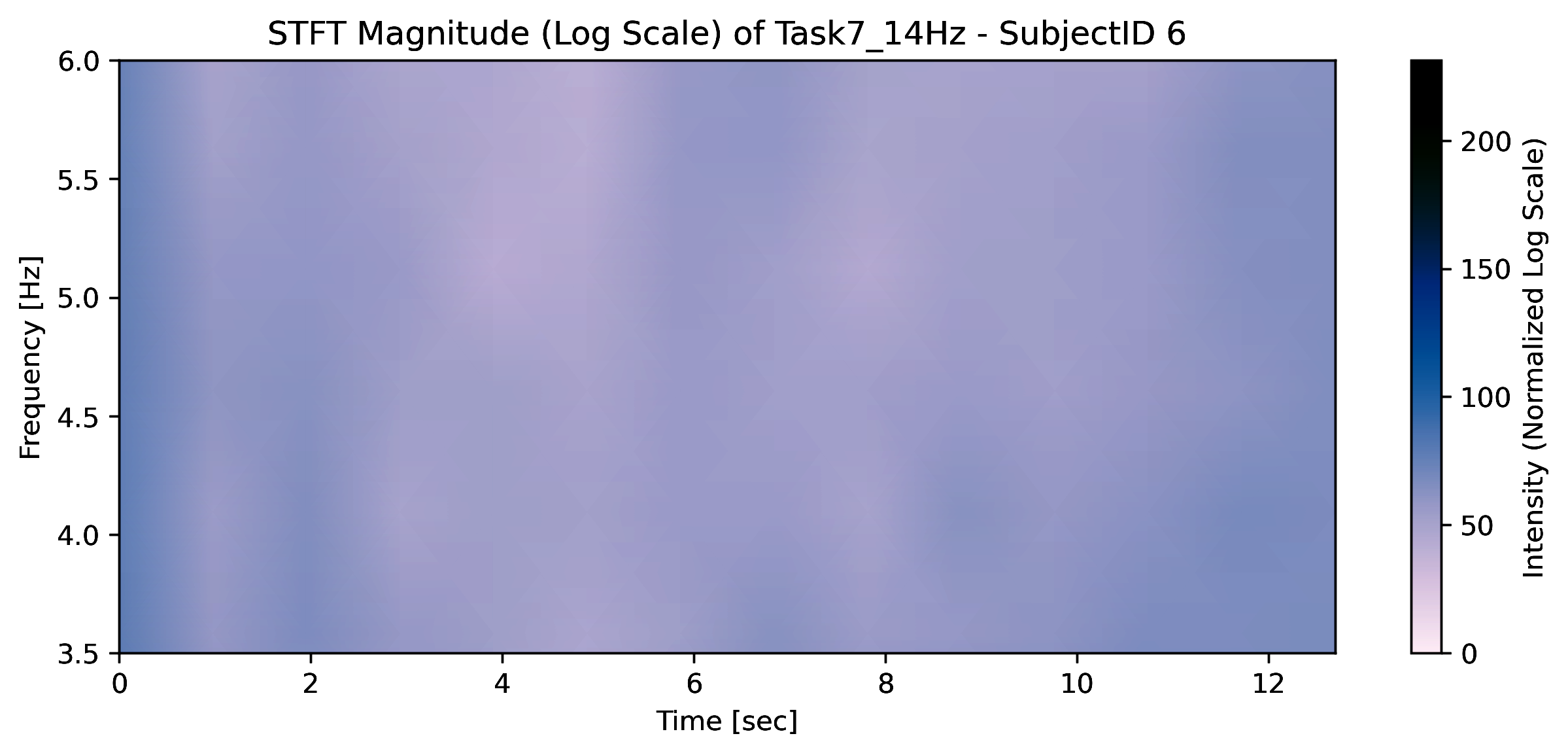

Supplement: Supplementary file 1 [file sensors-26-00157-s001.zip › STFT Images/RFG Images/Tasks 1-7 Images/Figure S15 Task 7 ID 6.png]
